# Demonstration of Green Solvent Performance on O,S,N-Heterocycles Synthesis: Metal-Free Click Chemistry and Buchwald—Hartwig Coupling

**DOI:** 10.3390/molecules26041074

**Published:** 2021-02-18

**Authors:** Joana F. Campos, Manon Cailler, Remi Claudel, Benjamin Prot, Thierry Besson, Sabine Berteina-Raboin

**Affiliations:** 1 Institut de Chimie Organique et Analytique (ICOA), Université d’Orléans UMR-CNRS 7311, BP 6759, Rue de Chartres, 45067 Orléans CEDEX 2, France; joana-filomena.mimoso-silva-de-campos@univ-orleans.fr (J.F.C.); manon.cailler@etu.univ-orleans.fr (M.C.); remi.claudel@etu.univ-orleans.fr (R.C.); benjamin.prot@etu.univ-orleans.fr (B.P.); 2 Normandie Univ., UNIROUEN, INSA Rouen, CNRS, COBRA UMR 6014, 76000 Rouen, France; thierry.besson@univ-rouen.fr

**Keywords:** eucalyptol, cyclopentyl methyl ether, 2-methyltetrahydrofuran, O,S,N-heterocycles, greener methodology, green solvent

## Abstract

The development of new and greener approaches to organic synthesis has been a trend in recent years. Continuing the latest publications of our team, in this work, we demonstrate the efficiency of three solvents: eucalyptol (1,8-cineole), cyclopentyl methyl ether (CPME), and 2-methyltetrahydrofuran (2-MeTHF) for the synthesis of O,S,N-heterocyclic compounds.

## 1. Introduction

**Figure 1:**
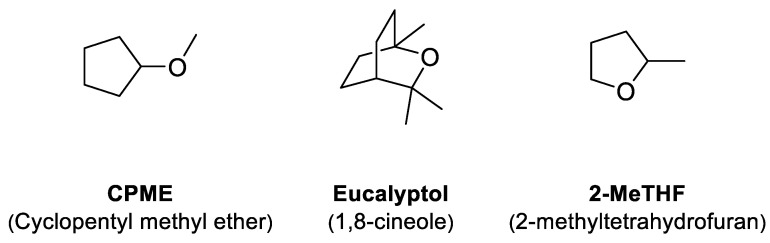
Green solvents used in this work.

The main goal of this work was to demonstrate the efficiency of new solvents as alternatives for the synthesis of O,S,N-heterocyclic compounds. In organic synthesis, the solvent is the component present in the greatest amount and is the basic element of the environmental performance of a process [1,2,3]. The search for alternative solvents with a lower environmental impact has increased in recent years, with several articles describing green solvents as effective alternatives to conventional petroleum solvents [4,5,6,7,8,9]. In our recent work, we have endeavored to show that eucalyptol could be an extremely interesting alternative as a solvent for various chemical transformations. Its use is all the more interesting as it contributes to the recycling of waste produced by the wood and paper industries. Eucalyptol is extracted from eucalyptus leaves, which are increasingly cultivated due to their rapid growth. Therefore it seemed relevant to compare it to other ether-type solvents deemed to be green. In order to continue to develop new and green methods to construct heterocyclic rings containing oxygen, sulfur, and nitrogen [10,11,12,13,14], we report here the use of eucalyptol (1,8-cineole), cyclopentyl methyl ether (CPME), and 2-methyltetrahydrofuran (2-MeTHF) as alternative solvents for metal-free click chemistry and Buchwald–Hartwig coupling (Figure 1). 2-methyltetrahydrofuran (2-MeTHF) is already well known to generate fewer peroxides than when tetrahydrofuran (THF) is obtained from renewable corncobs and bagasse resources. Cyclopentyl methyl ether (CPME) was also described to be a good alternative, more stable than THF and 2-MeTHF, and it has improved laboratory safety, minimizing the formation of peroxides.

**Figure 2:**
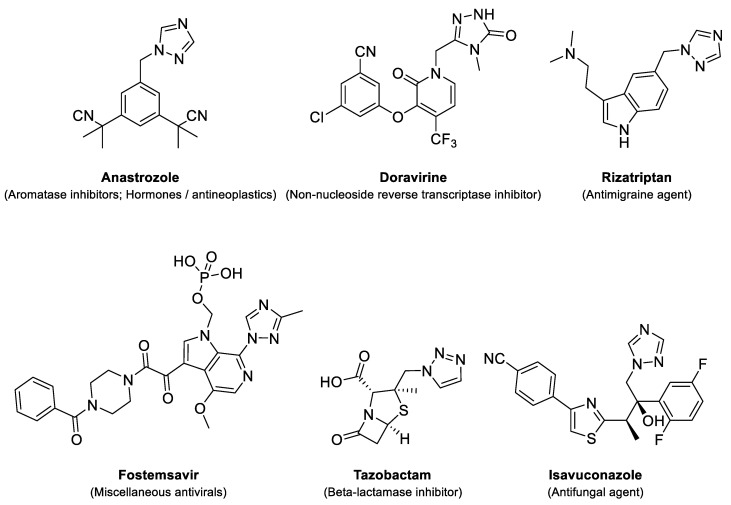
Structures of some Food and Drug Administration (FDA) approved drugs with heterocyclic scaffolds reported in this study.

Substituted triazole derivatives are an important class of nitrogen-fused heterocycles, which are abundant in many pharmaceutical compounds. Over the past decades, the Food and Drug Administration (FDA) has increasingly approved drugs with these skeletons for their important biological activities (Figure 2) [15,16].

**Figure 3:**
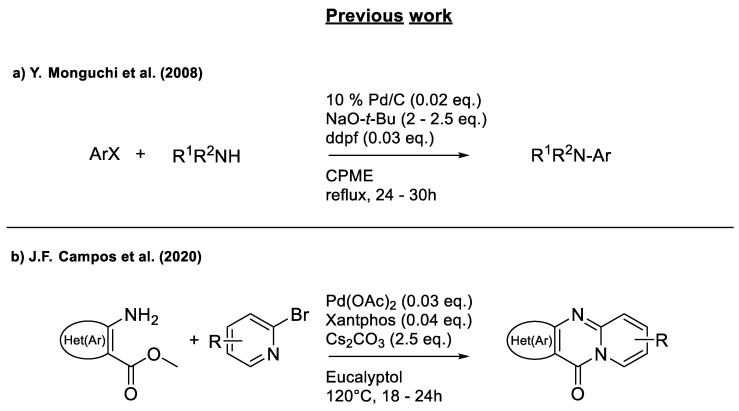
Study of scope and limitations of Buchwald–Hartwig Coupling/Pyridine Dearomatization Sequence from methyl 3-aminothiophene-2-carboxylate. (**a**) methodology developped by Y.Monguchi et al. (2008) (**b**) work reported by our team in 2020.

To the best of our knowledge, there is no report in the literature to date on the use of eucalyptol, CPME, and 2-MeTHF to construct O,S,N-heterocycles by metal-free click chemistry. Therefore it seemed relevant to study solvent alternatives for this methodology. A literature review on the Buchwald–Hartwig reaction, carried out by palladium catalysis, revealed two examples of the application of green solvents: CPME (Figure 3a) [17] and eucalyptol (Figure 3b) [10]. The objective of the present study was to further enhance the greener side and try to make its use compatible with microwave-assisted synthesis and, consequently, to reduce the duration of the reaction.

## 2. Results and Discussion

The study presented herein is divided into three parts, each of them corresponding to a type of reaction developed and evaluated in the different solvents investigated.

### 2.1. Metal-Free Click Chemistry

This part of the work was based on our previous conditions published in 2017 [18]. The intention was to make the reaction conditions more efficient and sustainable. A one-pot methodology was first successfully developed, using only toluene as solvent. The scope and limitations of the process were then analyzed using several propargyl derivatives. The reaction using toluene as a solvent served as a standard of comparison for the results obtained with the three green solvents. For this evaluation, several heterocyclic compounds containing oxygen, sulfur, and nitrogen were chosen as starting materials. The results obtained are given by the class of heterocycle.

#### 2.1.1. 7-Amino-2,3-dihydro-benzo[1,4]dioxine-6-carboxylic Acid Methylester

**Figure 4:**
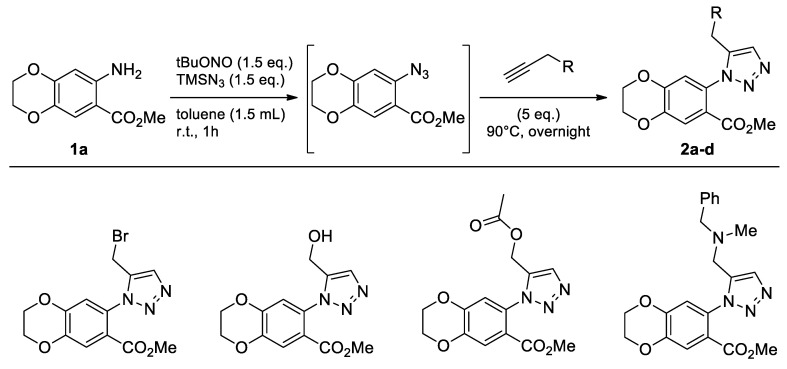
Study of scope and limitations of Metal-free Click Chemistry from 7-amino-2,3-dihydro-benzo[1,4]dioxine-6-carboxylic acid methyl ester.

The commercially available 7-amino-2,3-dihydro-benzo[1,4]dioxine-6-carboxylic acid methyl ester (**1a**) was reacted first with *t*BuONO and TMSN_3_ for 1 h, and then stirred with several propargyl derivatives in toluene without the use of any metal catalyst (Figure 4).

The new compounds **2a**–**d** were obtained in moderate to good yields. We focused on product **2a**, obtained from propargyl bromide because it can be functionalized at a later stage, which makes it possible to increase molecular diversity. Therefore, the next step was to test the yield of compound **2a** using these reaction conditions in the three green solvents selected (Table 1).

Compound **2a** was successfully obtained in the three experiments carried out in CPME, 2-MeTHF, and eucalyptol (Table 1) instead of toluene. The highest yield was obtained with 2-MeTHF (Table 1, Entry 2). Moreover, the yield increased from 57% with toluene to 70% when using a green solvent.

#### 2.1.2. Methyl 6-amino-1*H*-indazole-7-carboxylate

**Figure 5:**
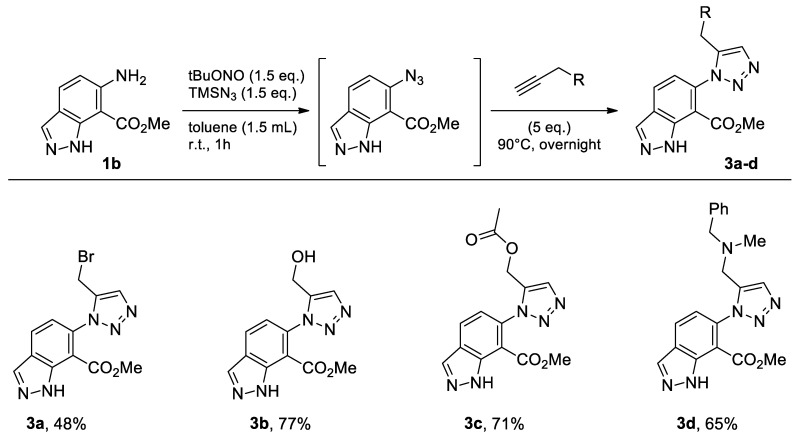
Study of scope and limitations of Metal-free Click Chemistry from methyl 6-amino-1*H*-indazole-7-carboxylate.

The commercially available methyl 6-amino-1*H*-indazole-7-carboxylate (**1b**) was submitted to the same conditions as 7-amino-2,3-dihydro-benzo[1,4]dioxine-6-carboxylic acid methyl ester (**1a**). In this case, also, we were able to synthesize the desired new compounds **3a**–**d** in moderate to good yields in toluene (Figure 5).

With this heterocycle derived from indazole, obtaining the brominated compound using CPME, 2-MeTHF and eucalyptol were only achieved with very average yields (Table 2, Entries 1–3). However, these results, although disappointing, were envisaged because the synthesis yield of this brominated derivative under conventional solvent (toluene) conditions was already low (48%) and not very far from that obtained with 2-MeTHF, or even with eucalyptol. 2-MeTHF was, in this case, also the solvent that achieved the best result (Table 1 and Table 2, Entry 2).

#### 2.1.3. Methyl 3-amino-5-phenylthiophene-2-carboxylate

**Figure 6:**
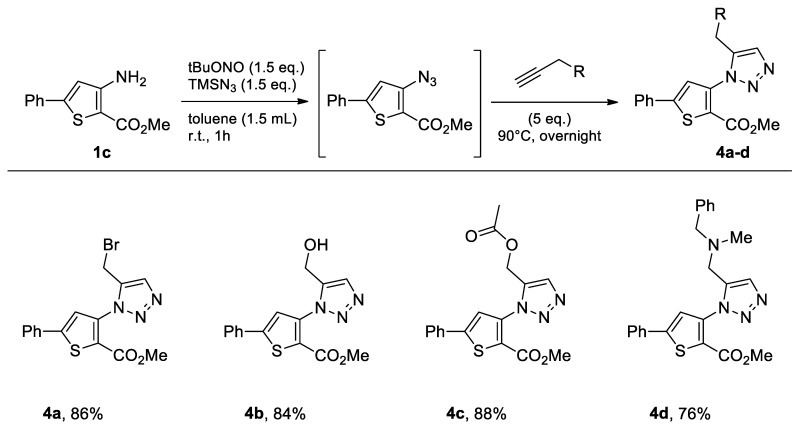
Study of scope and limitations of Metal-free Click Chemistry from methyl 3-amino-5-phenylthiophene-2-carboxylate.

The commercially available methyl 3-amino-5-phenylthiophene-2-carboxylate (**1c**) underwent the same metal-free click chemistry under the same conditions in the presence of various propargyl derivatives, leading to the expected compounds in very good yields (Figure 6).

With the same objective as in the previous series, the functionalizable brominated compound **4a** was synthesized in the three solvents. The results obtained were excellent, and in all green solvents, the yield was higher than that obtained with toluene as solvent (Table 3, Entries 1–3).

In view of the good results obtained from methyl 3-amino-5-phenylthiophene-2-carboxylate (**1c**) as starting material, we decided to use the green solvent that performed best (i.e., CPME) to test a microwave-assisted methodology. In the first stage of the one-pot process, the same reaction conditions were used since the reaction was easily carried out in 1 h at room temperature. Then, for the second step, the reaction was stirred for 1 h under microwave irradiation at 140 °C in a Biotage microwave apparatus. The result obtained using CPME as solvent did not allow us to carry out the reaction efficiently under microwave irradiation (Table 3, Entry 4).

#### 2.1.4. Methyl 3-aminothiophene-2-carboxylate

**Figure 7:**
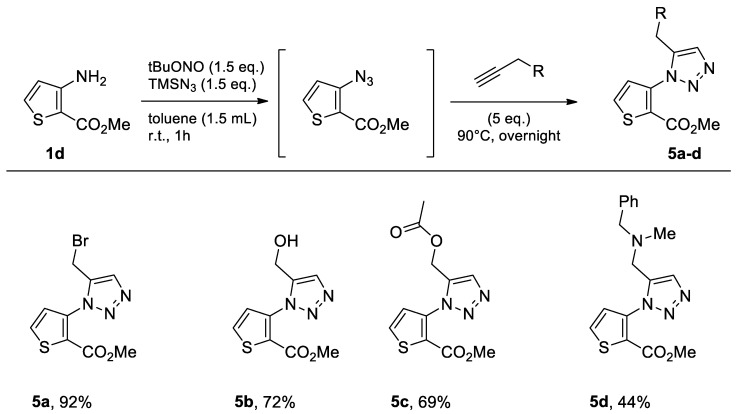
Study of scope and limitations of Metal-free Click Chemistry from methyl 3-aminothiophene-2-carboxylate.

Starting from commercially available methyl 3-aminothiophene-2-carboxylate (**1d**), we obtained the same results, and the desired compounds were synthesized in good to excellent yields (Figure 7).

As in the other series, the brominated product **5a** was tested using the three green solvents (Table 4, Entries 1–3). The highest yield was obtained with eucalyptol for this starting material. It gave an excellent result, higher than that presented by toluene. For this reason, we also tested a microwave-assisted methodology in eucalyptol, but again, the yield decreased considerably when, in step two of the one-pot process, the reaction was placed in the microwave at 140 °C for 1 h. The use of microwave irradiation did not seem effective under these conditions (Table 4, Entry 4).

#### 2.1.5. Methyl 4-amino-5-thiazolecarboxylate

**Figure 8:**
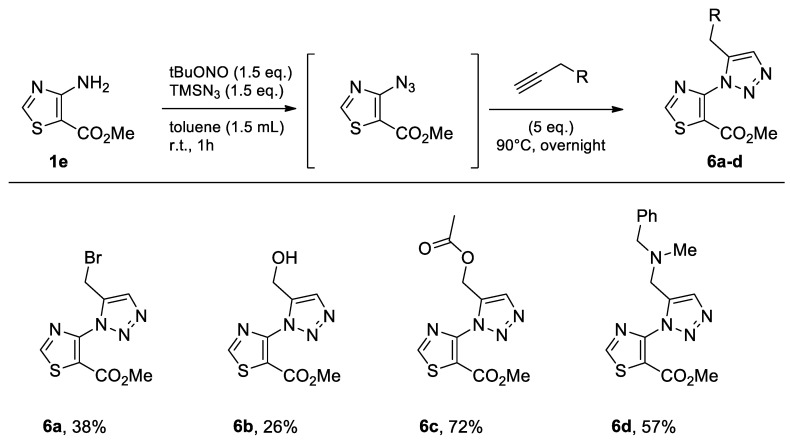
Study of scope and limitations of Metal-free Click Chemistry from methyl 4-amino-5-thiazolecarboxylate.

The commercially available methyl 4-amino-5-thiazolecarboxylate (**1e**) was submitted to the same protocol as the other classes of heterocycles in toluene (Figure 8). The reactivity of methyl 4-amino-5-thiazolecarboxylate (**1e**) was found to be lower than that of the other classes of heterocycles tested (Figure 8). One reason may be the lower solubility of the compound in the medium since even when the medium was diluted, no complete dissolution was ever observed.

Although the yield of the brominated compound **6a** in this series was only 38%, the same tests using the different solvents were carried out (Table 5).

Compound **6a** was obtained with the three solvents, but as expected, the yields were very low when compared to the other series of heterocycles performed earlier in this study (Table 5). In this case, the best yield was obtained with CPME.

To optimize conditions for the synthesis of heterocycles in green solvents using microwave irradiation as an activation method, we decided to use the conditions previously published by our team [10] for the synthesis of 10*H*-pyrido[1,2-*a*]thieno[3,2-*d*]pyrimidin-10-one.

### 2.2. Buchwald-Hartwig Coupling/Pyridine Dearomatization Sequence

**Figure 9:**
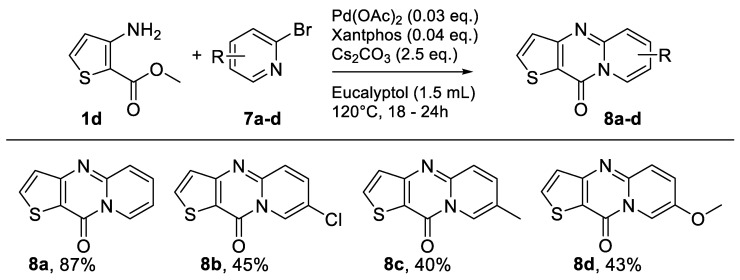
Study of scope and limitations of Buchwald–Hartwig Coupling/Pyridine Dearomatization Sequence from methyl 3-aminothiophene-2-carboxylate **1d.**

**Figure 10:**
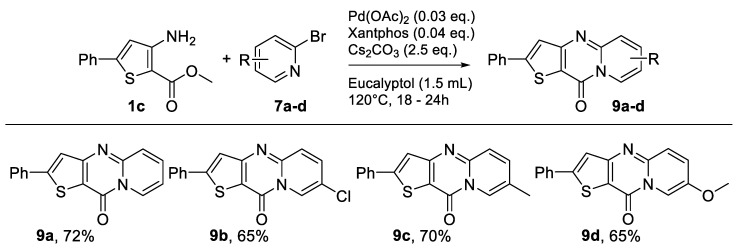
Study of scope and limitations of Buchwald–Hartwig Coupling/Pyridine Dearomatization Sequence from methyl 3-amino-5-phenylthiophene-2-carboxylate.

The purpose was to accomplish a microwave-assisted synthesis using green solvents to obtain 10*H*-pyrido[1,2-*a*]thieno[3,2-*d*]pyrimidin-10-one derivatives. For this examination, the prior methyl 3-aminothiophene-2-carboxylate (**1d**) and 3-amino-5-phenylthiophene-2-carboxylate (**1c**) were used as starting material. As demonstrated in [10], we knew that the results obtained in Microwave with eucalyptol were unsatisfactory, so we only tested toluene, CPME, and 2-MeTHF. In order to have standard results to serve as a reference for green solvents, the products were synthesized using eucalyptol (Figure 9 and Figure 10).

Starting from methyl 3-aminothiophene-2-carboxylate (**1d**) or methyl 3-amino-5-phenylthiophene-2-carboxylate (**1c**), eight new compounds were obtained in acceptable yields (Figure 9 and Figure 10).

As explained earlier, the objective in this section was to find a methodology that combined the use of a green solvent with the use of microwave irradiation. For this optimization process (Table 6), we chose compound **8a** as it showed the highest yield when obtained through classical heating with eucalyptol as an alternative solvent.

The optimization protocol started by using the same reaction conditions (Figure 8), changing only the solvent of the reaction mixture (Table 6, Entries 1–3). The desired product was obtained in good yield. Changing the solvent did not interfere with the reaction performance, and using 2-MeTHF led to a better yield than with eucalyptol (Figure 9; Table 6, Entry 2). Tests were next performed to estimate the compatibility of the two green solvents (CPME and 2-MeTHF) in a microwave-assisted reaction (Table 6, Entries 4 and 5). Toluene was used as a reference for a conventional solvent (Table 6, Entry 6). From methyl 3-aminothiophene-2-carboxylate using Pd(OAc)_2_ (3mol%), Xantphos (4 mol%), and Cs_2_CO_3_ (2.5 equiv) at 140 °C for 1 h under microwave irradiation, the product was obtained in all solvents (Table 6, Entries 4–6). However, yields were slightly lower than reactions stirred in a classical heating system (Table 6, Entries 1–6). It should be noted that the reaction with CPME and 2-MeTHF was not complete, unlike that observed in toluene, which may explain this drop in yield (Table 6, Entries 4 and 5). To check this and try to complete the reactions in green solvents, the microwave irradiation was increased to 2 h (Table 6, Entries 7 and 8). With a longer reaction time, the conversion was total (no traces of starting materials), but there were signs of degradation. After purification of the desired product, the yields remained unchanged (Table 6, Entries 4–5 and 7–8). As the increase in the reaction time did not allow a significant improvement, we kept the initial reaction time to limit possible degradation of the medium while increasing the temperature of the reaction to 160 °C so as to enhance the reaction speed. The results were satisfactory (Table 6, Entries 9 and 10). CPME showed an improved yield (Table 6, Entry 9) with 1 h of microwave irradiation at 160 °C. With2-MeTHF, the yield obtained was similar (Table 6, Entry 10) but in a shorter reaction time at 160° compared to 140 °C, where the reaction was not complete.

With this optimization study (Table 6), we can conclude that CPME and 2-MeTHF are valid options for this reaction and allow a significant reduction in the reaction time, even if it is at the expense of a slight decrease in the yield of the desired final product.

The same optimization process was used from 3-amino-5-phenylthiophene-2-carboxylate (**1c**) under the same reaction conditions (Figure 10), just changing the solvent of the reaction mixture. The desired product was obtained in good to excellent yields (Table 7, Entries 1–3). The change of solvent improved the yield, and for all tests, the results were higher than those obtained with eucalyptol (Figure 10; Table 7, Entries 1–3). After performing the method with classical heating, we tested for each solvent the best conditions found in the study carried out from 3-aminothiophene-2-carboxylate (Table 6, Entries 6, 8 and 9).

The desired product **9a** was obtained, but the reaction was not complete; the presence of starting product was still observed (Table 7, Entries 4, 6, and 8). Under microwave irradiation, for the three solvents, it was necessary to double the reaction time to obtain a total conversion (Table 7, Entries 5, 7, and 9). In this case, among all the solvents tested in the reaction involving 3-amino-5-phenylthiophene-2-carboxylate, CPME showed by far the highest yields in both heating systems (Table 7, Entries 1 and 9).

### 2.3. Buchwald–Hartwig Amination of Bromo Derivatives *(**2**–**6a**)*

In the last part of our study, the proposed objective was to functionalize the brominated products synthesized previously. For this purpose, the reaction was first optimized using the compound **4a** and aniline. The choice of the catalytic system, base, and temperature was based on our previous work [12]. Once again, toluene was used as a conventional solvent reference (Table 8, Entry 1).

**Figure 11:**
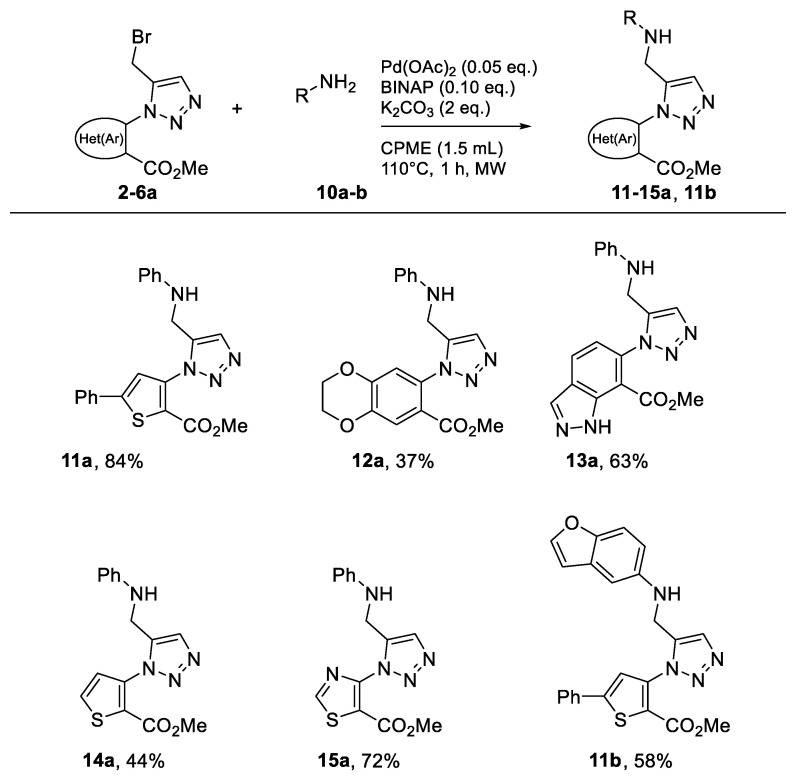
Study of scope and limitations of Buchwald–Hartwig Coupling.

The desired product **11a** was obtained, but the yield was below expectations (Table 8, Entry 1). A brief literature review showed that in some cases, it might be beneficial to carry out the reaction at room temperature [19,20]. To test the effectiveness of this aspect and to be able to apply it in our study, the reaction was launched with the solvent commonly used in these cases, *N,N*-dimethylformamide. The base selected was K_2_CO_3_, reported to be the right choice in these reaction systems [19]. After these changes in the reaction conditions, the yield improved satisfactorily (Table 8, Entry 2). To confirm the influence of the base, a test was performed replacing K_2_CO_3_ with Cs_2_CO_3_ (Table 8, Entry 3). The yield decreased, so K_2_CO_3_ was kept as the base. Finally and after having all the conditions in hand, we proceeded to determine the performance of the three solvents. The product was obtained in good yield in toluene and CPME (Table 8, Entries 4 and 6). The reaction stirred in 2-MeTHF led to the lowest yield and the longest reaction time, five days (Table 8, Entry 6). CPME was therefore chosen for the tests under MW. The aim was to see if it would be possible to significantly reduce the reaction time found in classic heating, i.e., three days. Surprisingly, stirring the reaction mixture for 1 h at 110 °C with microwave irradiation resulted in a complete reaction and consequently a good yield of the desired product (Table 8, Entry 7). The initial goal had been successfully achieved. Based on our results, the scope and limitations of this procedure were assessed using aniline and the brominated compounds synthesized earlier in this study (Figure 11).

Several methyl-{[(hetero)aryl-methyl]-1*H*-1,2,3-triazol-1-yl}-(hetero)aryl-carboxylate derivatives (**11**–**15a** and **11b**) were synthesized in moderate to good yields, demonstrating the generalizability of this method (Figure 11). We also used 4-aminobenzofuran (**10b**) to diversify the type of amine in our series and demonstrate the potential of the methodology.

## 3. Materials and Methods

### 3.1. General Methods

All reagents were purchased from commercial suppliers Sigma Aldrich, St Quentin Fallavier CEDEX, France; Fluorochem, Derbyshire, SK131QH, UK, and were used without further purification. The reactions were monitored by thin-layer chromatography (TLC) analysis using silica gel (60 F254) plates. Compounds were visualized by UV irradiation (Merck, St Quentin Fallavier CEDEX, France). Flash column chromatography was performed on silica gel 60 (230–400 mesh, 0.040–0.063 mm). Melting points (mp (°C)) were taken on samples in open capillary tubes and are uncorrected. ^1^H- and ^13^C- nuclear magnetic resonance (NMR) spectra were recorded on a Bruker AVANCE II spectrometer (Bruker, Wissembourg, France) at 250 MHz (^13^C, 62.9 MHz) and on a Bruker AVANCE III HD nanobay (Bruker, Wissembourg, France) 400 MHz (^13^C 100.62 MHz). Chemical shifts are given in parts per million from tetramethylsilane (TMS) or deuterated solvent (MeOH-d4, Chloroform-d) as an internal standard. The following abbreviations are used for the proton spectra multiplicities: b: broad, s: singlet, d: doublet, t: triplet, q: quartet, p: pentuplet, and m: multiplet. Coupling constants (J) are reported in Hertz (Hz). Multiplicities were determined by the DEPT 135 sequence. High-resolution mass spectra (HRMS) were performed on a Maxis UHR-q-TOF mass spectrometer (Bruker, Wissembourg, France) Bruker 4G with an electrospray ionisation (ESI) mode (Bruker, Wissembourg, France).

### 3.2. General Procedure for the Synthesis of Compounds ***2**–**6a**–**d***

A solution of methyl anthranilate (50 mg, 1 equiv.) in toluene (50 mL) was cooled to 0 °C, and *t*-BuONO (1.5 equiv.) followed by TMSN_3_ (1.5 equiv.) were added dropwise. The resulting solution was stirred at r.t. for 1 h. Then, the respective alkyne derivative (5 equiv.) was added, and the reaction mixture was heated at 90 °C overnight. After completion, the mixture was concentrated under vacuum. The solid obtained was purified by flash chromatography. The solvent polarity was increased via a gradient from neat petroleum ether to a mixture of ethyl acetate/petroleum ether (Supplementary Materials).

*Methyl 7-(5-(bromomethyl)-1H-1,2,3-triazol-1-yl)-2,3-dihydrobenzo[b][1,4]dioxine-6-carboxylate***2a***:* white solid (48 mg, 57%), m.p. 133–135 °C. ^1^H-NMR (400 MHz, CDCl_3_) δ 3.65 (s, 3H), 4.32–4.35 (m, 4H), 4.65 (s, 2H), 6.97 (s, 1H), 7.57 (s, 1H), 7.77 (s, 1H) ppm. ^13^C-NMR (101 MHz, CDCl_3_) δ 21.7 (CH), 52.4 (CH), 64.3 (CH), 64.7 (CH), 116.8 (CH), 120.1 (C), 120.6 (CH), 125.5 (CH), 130.1 (C), 143.9 (C), 144.5 (C), 146.9 (C), and 164.5 (C) ppm. HRMS: calculated (calcd) for C_13_H_13_BrN_3_O_4_ [M + H]^+^ 354.0084, found 354.0082.

*Methyl 7-(5-(hydroxymethyl)-1H-1,2,3-triazol-1-yl)-2,3-dihydrobenzo[b][1,4]dioxine-6-carboxylate***2b***:* white solid (61 mg, 88%), m.p. 204–206 °C.^1^H-NMR (400 MHz, CDCl_3_) δ 7.73 (s, 1H), 3.64 (s, 3H), 4.33 (s, 4H), 4.85 (s, 2H), 6.93 (d, *J* = 10.7 Hz, 1H), 7.57 (d, *J* = 9.2 Hz, 1H) ppm. ^13^C-NMR (101 MHz, CDCl_3_) δ 52.4 (CH), 56.4 (CH), 64.3 (CH), 64.7 (CH), 116.7 (CH), 120.0 (C), 120.4 (CH), 124.3 (CH), 130.5 (C), 144.3 (C), 146.8 (C), 147.1 (C), and 164.6 (C) ppm. HRMS: calcd for C_13_H_14_N_3_O_5_ [M + H]^+^ 292.0928, found 292.0933.

*Methyl 7-(5-(acetoxymethyl)-1H-1,2,3-triazol-1-yl)-2,3-dihydrobenzo[b][1,4]dioxine-6-carboxylate***2c***:* white solid (41 mg, 51%), m.p. 141–143 °C.^1^H-NMR (400 MHz, CDCl_3_) δ 2.08 (s, 3H), 3.64 (s, 3H), 4.33 (s, 4H), 5.27 (s, 2H), 6.96 (s, 1H), 7.57 (s, 1H), 7.79 (s, 1H) ppm. ^13^C-NMR (101 MHz, CDCl_3_) δ 20.9 (CH), 52.3 (CH), 57.6 (CH), 64.3 (CH), 64.7 (CH), 116.8 (CH), 120.0 (C), 120.5 (CH), 126.3 (CH), 130.3 (C), 142.2 (C), 144.4 (C), 146.8 (C), 164.5 (C), and 170.8 (C) ppm. HRMS: calcd for C_15_H_16_N_3_O_6_ [M + H]^+^ 334.1034, found 334.1033.

*Methyl 7-(5-((benzyl(methyl)amino)methyl)-1H-1,2,3-triazol-1-yl)-2,3-dihydrobenzo[b][1,4]dioxine-6-carboxylate***2d***:* yellow oil (47 mg, 50%).^1^H-NMR (400 MHz, CDCl_3_) δ 2.29 (s, 3H), 3.60 (s, 2H), 3.62 (s, 3H), 3.80 (s, 2H), 4.31–4.34 (m, 4H), 6.96 (s, 1H), 7.21–7.37 (m, 5H), 7.56 (s, 1H), 7.67 (s, 1H) ppm.^13^C-NMR (101 MHz, CDCl_3_) δ 42.1 (CH), 52.0 (CH), 52.3 (CH), 61.3 (CH), 64.3 (CH), 64.6 (CH), 116.7 (CH), 120.2 (C), 120.4 (CH), 125.0 (CH), 127.0 (CH), 128.3 (2 × CH), 129.0 (2 × CH), 130.6 (C), 138.8 (C), 144.2 (C), 144.6 (C), 146.7 (C), and 164.7 (C) ppm. HRMS: calcd for C_21_H_23_N_4_O_4_ [M + H]^+^ 395.1714, found 395.1712.

*Methyl 6-(5-(bromomethyl)-1H-1,2,3-triazol-1-yl)-1H-indazole-7-carboxylate***3a***:* white solid (42mg, 48%), m.p. 287–289 °C.^1^H-NMR (400 MHz, CDCl_3_) δ 3.77 (s, 3H), 4.71 (s, 2H), 7.28 (d, *J* = 8.3 Hz, 1H), 7.88 (s, 1H), 8.10 (d, *J* = 8.3 Hz, 1H), 8.26 (s, 1H), 11.50 (s, 1H) ppm. ^13^C-NMR (101 MHz, CDCl_3_) δ 21.4 (CH), 52.9 (CH), 109.1 (C), 120.7 (CH), 125.2 (C), 125.5 (CH), 126.5 (CH), 135.4 (CH), 135.6 (C), 139.3 (C), 144.4 (C), and 164.7 (C) ppm. HRMS: calcd for C_12_H_11_BrN_5_O_2_ [M + H]^+^ 336.0091, found 336.0088.

*Methyl 6-(5-(hydroxymethyl)-1H-1,2,3-triazol-1-yl)-1H-indazole-7-carboxylate***3b***:* white solid (55 mg, 77%), m.p. 177–179 °C. ^1^H-NMR (400 MHz, CDCl_3_) δ 3.80 (d, *J* = 3.5 Hz, 3H), 7.28 (d, *J* = 1.8 Hz, 2H), 7.86 (s, 1H), 8.13 (dd, *J* = 10.8, 8.3 Hz, 1H), 8.29 (d, *J* = 5.7 Hz, 1H), 11.45 (s, 1H) ppm. ^13^C-NMR (101 MHz, CDCl_3_) δ 21.4 (CH), 52.9 (CH), 109.1 (C), 120.7 (CH), 125.2 (C), 125.5 (CH), 126.5 (CH), 135.4 (CH), 135.6 (C), 139.3 (C), 144.4 (C), and 164.7 (C) ppm. HRMS: calcd for C_12_H_12_N_5_O_3_ [M + H]^+^ 274.0935, found 274.0938.

*Methyl 6-(5-(acetoxymethyl)-1H-1,2,3-triazol-1-yl)-1H-indazole-7-carboxylate***3c***:* white solid (66 mg, 71%), m.p. 132–134 °C. ^1^H-NMR (400 MHz, CDCl_3_) δ 2.09 (s, 3H), 3.73 (s, 3H), 5.32 (s, 2H), 7.23 (d, *J* = 8.3 Hz, 1H), 7.91 (s, 1H), 8.05 (d, *J* = 8.3 Hz, 1H), 8.23 (s, 1H), 11.79 (s, 1H) ppm. ^13^C-NMR (101 MHz, CDCl_3_) δ 20.9 (CH), 52.8 (CH), 57.6 (CH), 120.6 (CH), 109.0 (C), 125.1 (C), 126.4 (CH), 126.4 (CH), 135.3 (CH), 135.7 (C), 139.2 (C), 142.5 (C), 164.7 (C), and 170.9 (C) ppm. HRMS: calcd for C_14_H_14_N_5_O_4_ [M + H]^+^ 316.1040, found 316.1040.

*Methyl 6-(5-((benzyl(methyl)amino)methyl)-1H-1,2,3-triazol-1-yl)-1H-indazole-7-carboxylate***3d***:* yellow oil (64 mg, 65%). ^1^H-NMR (400 MHz, CDCl_3_) δ 2.35 (s, 3H), 3.66 (s, 2H), 3.74 (s, 3H), 3.89 (s, 2H), 7.27–7.41 (m, 6H), 7.82 (s, 1H), 8.08 (d, *J* = 8.3 Hz, 1H), 8.27 (s, 1H), 11.78 (s, 1H) ppm.^13^C-NMR (101 MHz, CDCl_3_) δ 42.2 (CH), 52.0 (CH), 52.8 (CH), 61.3 (CH), 109.0 (C), 120.6 (CH), 125.0 (C), 125.1 (CH), 126.3 (CH), 127.1 (CH), 128.3 (2 × CH), 129.0 (2 × CH), 135.3 (CH), 136.1 (C), 138.7 (C), 139.3 (C), 145.1 (C), and 164.9 (C) ppm. HRMS: calcd for C_20_H_21_N_6_O_2_ [M + H]^+^ 377.1721, found 377.1722.

*Methyl 3-(5-(bromomethyl)-1H-1,2,3-triazol-1-yl)-5-phenylthiophene-2-carboxylate***4a***:* white solid (70mg, 86%), m.p. 138–140 °C. ^1^H-NMR (400 MHz, CDCl_3_) δ 3.86 (s, 3H), 4.67 (s, 2H), 7.41–7.46 (m, 3H), 7.64 (dd, *J* = 7.7, 1.7 Hz, 2H), 7.73 (s, 1H), 8.52 (s, 1H) ppm.^13^C-NMR (101 MHz, CDCl_3_) δ 21.5 (CH), 52.7 (CH), 119.6 (C), 121.8 (CH), 125.7 (CH), 126.0 (2 × CH), 129.3 (2 × CH), 129.8 (CH), 132.0 (C), 138.4 (C), 143.8 (C), 149.4 (C), and 160.7 (C) ppm. HRMS: calcd for C_15_H_13_BrN_3_O_2_S [M + H]^+^ 377.9906, found 377.9906.

*Methyl 3-(5-(hydroxymethyl)-1H-1,2,3-triazol-1-yl)-5-phenylthiophene-2-carboxylate***4b***:* white solid (57mg, 84%), m.p. 161–163°C. ^1^H-NMR (400 MHz, CDCl_3_) δ 3.87 (s, 3H), 4.90 (s, 2H), 7.44 (td, *J* = 5.5, 2.8 Hz, 3H), 7.66 (dd, *J* = 7.9, 1.6 Hz, 2H), 7.73 (s, 1H), 8.43 (s, 1H) ppm. ^13^C-NMR (101 MHz, CDCl_3_) δ 52.6 (CH), 56.6 (CH), 119.6 (C), 122.0 (CH), 124.6 (CH), 126.1 (2 × CH), 129.3 (2 × CH), 129.8 (CH), 132.1 (C), 138.7 (C), 146.8 (C), 149.3 (C), and 160.7 (C) ppm. HRMS: calcd for C_15_H_14_N_3_O_3_S [M + H]^+^ 316.0750, found 316.0744.

*Methyl 3-(5-(acetoxymethyl)-1H-1,2,3-triazol-1-yl)-5-phenylthiophene-2-carboxylate***4c:** white solid (67 mg, 88%), m.p. 147–149°C. ^1^H-NMR (400 MHz, CDCl_3_) δ 2.09 (s, 3H), 3.85 (s, 3H), 5.30 (s, 2H), 7.40–7.45 (m, 3H), 7.62–7.65 (m, 2H), 7.70 (s, 1H), 8.49 (s, 1H) ppm. ^13^C-NMR (101 MHz, CDCl_3_) δ 20.9 (CH), 52.6 (CH), 57.5 (CH), 119.7 (C), 121.9 (CH), 126.0 (2 × CH), 126.6 (CH), 129.3 (2 × CH), 129.8 (CH), 132.0 (C), 138.5 (C), 142.0 (C), 149.3 (C), 160.6 (C), and 170.8 (C) ppm. HRMS: calcd for C_17_H_16_N_3_O_4_S [M + H]^+^ 358.0856, found 358.0850.

*Methyl 3-(5-((benzyl(methyl)amino)methyl)-1H-1,2,3-triazol-1-yl)-5-phenylthiophene-2-carboxylate***4d**: yellow oil (68 mg, 76%). ^1^H-NMR (400 MHz, CDCl_3_) δ 2.35 (s, 3H), 3.65 (s, 2H), 3.88 (s, 5H), 7.27 –7.31 (m, 1H), 7.35 (t, *J* = 7.3 Hz, 2H), 7.45 (ddd, *J* = 13.4, 8.7, 6.5 Hz, 5H), 7.67–7.70 (m, 2H), 7.77 (s, 1H), 8.40 (s, 1H) ppm. ^13^C-NMR (101 MHz, CDCl_3_) δ 42.1 (CH), 51.9 (CH), 52.5 (CH), 61.2 (CH), 119.5 (C), 122.0 (CH), 125.6 (CH), 126.1 (2xCH), 127.1 (CH), 128.3 (2 × CH), 129.1 (2 × CH), 129.3 (2 × CH), 129.7 (CH), 132.1 (C), 138.8 (C), 138.9 (C), 144.3 (C), 149.2 (C), and 160.7 (C) ppm. HRMS: calcd for C_23_H_23_N_4_O_2_S [M + H]^+^ 419.1536, found 419.1540.

*Methyl 3-(5-(bromomethyl)-1H-1,2,3-triazol-1-yl)thiophene-2-carboxylate***5a***:* yellow solid (89mg, 92%), m.p. 121–123 °C. ^1^H-NMR (400 MHz, CDCl_3_) δ 3.81 (s, 3H), 4.62 (s, 2H), 7.47 (d, *J* = 5.4 Hz, 1H), 7.60 (d, *J* = 5.4 Hz, 1H), 8.42 (s, 1H) ppm. ^13^C-NMR (101 MHz, CDCl_3_) δ 21.5 (CH), 52.7 (CH), 122.0 (C), 125.7 (CH), 126.4 (CH), 131.3 (CH), 138.0 (C), 143.7 (C), 160.6 (C) ppm (Chemical Abstracts Service Registry Number: 1203476-09-8).

*Methyl 3-(5-(hydroxymethyl)-1H-1,2,3-triazol-1-yl)thiophene-2-carboxylate***5b***:* white solid (55mg, 72%), m.p. 94–96 °C. ^1^H-NMR (400 MHz, CDCl_3_) δ 3.84 (s, 3H), 4.88 (d, *J* = 5.0 Hz, 2H), 7.49 (d, *J* = 5.4 Hz, 1H), 7.61 (d, *J* = 5.4 Hz, 1H), 8.34 (s, 1H) ppm. ^13^C-NMR (101 MHz, CDCl_3_) δ 52.6 (CH), 56.4 (CH), 122.0 (C), 124.6 (CH), 126.6 (CH), 131.1 (CH), 138.4 (C), 146.9 (C), and 160.6 (C) ppm (CAS: 1203476-11-2).

*Methyl 3-(5-(acetoxymethyl)-1H-1,2,3-triazol-1-yl)thiophene-2-carboxylate***5c***:* white solid (62 mg, 69%), m.p. 163–165 °C. ^1^H-NMR (400 MHz, CDCl_3_) δ 2.06 (s, 3H), 3.82 (s, 3H), 5.26 (s, 2H), 7.47 (d, *J* = 5.4 Hz, 1H), 7.60 (d, *J* = 5.4 Hz, 1H), 8.40 (s, 1H) ppm. ^13^C-NMR (101 MHz, CDCl_3_) δ 20.9 (CH), 52.6 (CH), 57.5 (CH), 122.0 (C), 126.5 (CH), 126.6 (CH), 131.2 (CH), 138.1 (C), 142.0 (C), 160.5 (C), and 170.7 (C) ppm. HRMS: calcd for C_11_H_12_N_3_O_4_S [M + H]^+^ 282.0543, found 282.0540.

*Methyl 3-(5-((benzyl(methyl)amino)methyl)-1H-1,2,3-triazol-1-yl)thiophene-2-carboxylate***5d***:* white solid (48 mg, 44%), m.p. 181–183 °C. ^1^H-NMR (400 MHz, CDCl_3_) δ 2.34 (s, 3H), 3.65 (s, 2H), 3.87 (s, 5H), 7.26–7.30 (m, 1H), 7.35 (t, *J* = 7.4 Hz, 2H), 7.40–7.43 (m, 2H), 7.56 (d, *J* = 5.4 Hz, 1H), 7.64 (d, *J* = 5.4 Hz, 1H), 8.34 (s, 1H) ppm. ^13^C-NMR (101 MHz, CDCl_3_) δ 42.1 (CH), 51.9 (CH), 52.5 (CH), 61.2 (CH), 121.8 (C), 125.5 (CH), 126.6 (CH), 127.0 (CH), 128.3 (2 × CH), 129.1 (2 × CH), 131.0 (CH), 138.6 (C), 138.8 (C), 144.3 (C), and 160.6 (C) ppm. HRMS: calcd for C_17_H_19_N_4_O_2_S [M + H]^+^ 343.1223, found 343.1216.

*Methyl 4-(5-(bromomethyl)-1H-1,2,3-triazol-1-yl)thiazole-5-carboxylate***6a***:* white solid (36 mg, 38%), m.p. 123–125 °C. ^1^H-NMR (400 MHz, CDCl_3_) δ 3.87 (s, 3H), 4.67 (s, 2H), 8.27 (s, 1H), 8.95 (s, 1H) ppm. ^13^C-NMR (101 MHz, CDCl_3_) δ 159.6 (C), 155.9 (CH), 147.4 (C), 144.1 (C), 124.8 (CH), 119.6 (C), 53.2 (CH), and 21.1 (CH) ppm. HRMS: calcd for C_8_H_8_BrN_4_O_2_S [M + H]^+^ 302.9546, found 302.9546.

*Methyl 4-(5-(hydroxymethyl)-1H-1,2,3-triazol-1-yl)thiazole-5-carboxylate***6b***:* white solid (20mg, 26%), m.p. 95–97 °C. ^1^H-NMR (400 MHz, CDCl_3_) δ 3.86 (s, 3H), 4.91 (d, *J* = 5.7 Hz, 2H), 8.20 (s, 1H), 8.93 (s, 1H) ppm. ^13^C-NMR (101 MHz, CDCl_3_) δ 53.2 (CH), 56.5 (CH), 119.3 (C), 123.6 (CH), 147.2 (C), 147.7 (C), 155.7 (CH), and 159.7 (C) ppm. HRMS: calcd for C_8_H_9_N_4_O_3_S [M + H]^+^ 241.0390, found 241.0390.

*Methyl 4-(5-(acetoxymethyl)-1H-1,2,3-triazol-1-yl)thiazole-5-carboxylate***6c***:* yellow oil (64 mg, 72%). ^1^H-NMR (400 MHz, CDCl_3_) δ 2.08 (s, 3H), 3.84 (s, 3H), 5.29 (s, 2H), 8.25 (s, 1H), 8.93 (s, 1H) ppm. ^13^C-NMR (101 MHz, CDCl_3_) δ 20.9 (CH), 53.2 (CH), 57.4 (CH), 119.5 (C), 125.7 (CH), 142.3 (C), 147.5 (C), 155.9 (CH), 159.6 (C), and 170.8 (C) ppm. HRMS: calcd for C_10_H_11_N_4_O_4_S [M + H]^+^ 283.0496, found 283.0495.

*Methyl 4-(5-((benzyl(methyl)amino)methyl)-1H-1,2,3-triazol-1-yl)thiazole-5-carboxylate***6d***:* yellow oil (62 mg, 57%). ^1^H-NMR (400 MHz, CDCl_3_) δ 2.34 (s, 3H), 3.65 (s, 2H), 3.88 (d, *J* = 5.3 Hz, 5H), 7.27–7.42 (m, 5H), 8.18 (s, 1H), 8.97 (s, 1H), ppm. ^13^C-NMR (101 MHz, CDCl_3_) δ 42.1 (CH), 51.8 (CH), 53.1 (CH), 61.3 (CH), 119.3 (C), 124.5 (CH), 127.1 (CH), 128.3 (2 × CH), 129.0 (2 × CH), 138.7 (C), 144.8 (C), 147.9 (C), 155.7 (CH), and 159.7 (C) ppm. HRMS: calcd for C_16_H_18_N_5_O_2_S [M + H]^+^ 344.1176, found 344.1172.

### 3.3. General Procedure for the Synthesis of Compounds ***8*** and ***9a**–**d***

A mixture of methyl anthranilate (50 mg, 1 equiv.), 2-bromopyridine (2 equiv.), Pd(OAc)_2_ (0.03 equiv.), Xantphos (0.04 equiv.), and Cs_2_CO_3_ (2.5 equiv.) in eucalyptol (1.5 mL) was stirred at 120 °C for 18–24 h. The reaction was followed by TLC. After completion, the reaction was then cooled to room temperature, and the mixture was concentrated under vacuum. The solid obtained was purified by flash chromatography using a mixture of ethyl acetate/petroleum ether.

*10H-pyrido[1,2-a]thieno[3,2-d]pyrimidin-10-one***8a**: Yellow solid (56 mg, 87%), m.p. 208–210 °C. ^1^H-NMR (400 MHz, CDCl_3_) δ 6.99 (ddd, *J* = 7.6, 5.3, 2.6 Hz, 1H), 7.35 (d, *J* = 5.3 Hz, 1H), 7.56–7.61 (m, 2H), 7.91 (d, *J* = 5.3 Hz, 1H), 8.97 (d, *J* = 7.3 Hz, 1H) ppm. ^13^C-NMR (101 MHz, CDCl_3_) δ 113.6 (CH), 115.4 (C), 124.9 (CH), 126.0 (CH), 126.3 (CH), 134.3 (CH), 136.6 (CH), 149.0 (C), 154.3 (C), and 157.8 (C) ppm (CAS: 1934255-23-8).

*7-Chloro-10H-pyrido[1,2-a]thieno[3,2-d]pyrimidin-10-one***8b**: Yellow solid (34 mg, 45%), m.p. 195–197 °C. ^1^H-NMR (400 MHz, CDCl_3_) δ 7.39 (d, *J* = 5.3 Hz, 1H), 7.55 (dd, *J* = 2.9, 1.5 Hz, 2H), 7.96 (d, *J* = 5.3 Hz, 1H), 9.00–9.05 (m, 1H) ppm. ^13^C-NMR (101 MHz, CDCl_3_) δ 115.9 (C), 122.2 (C), 124.0 (CH), 125.0 (CH), 127.1 (CH), 135.6 (CH), 136.9 (CH), 147.3 (C), and 153.4 (C), 157.6 (C) ppm. HRMS: calcd for C_10_H_6_ClN_2_OS [M + H]^+^ 236.9884, found 236.9885.

*7-Methyl-10H-pyrido[1,2-a]thieno[3,2-d]pyrimidin-10-one***8c**: White solid (28 mg, 40%), m.p. 177–179 °C. ^1^H-NMR (400 MHz, CDCl_3_) δ 2.42 (s, 3H), 7.38 (d, *J* = 5.4 Hz, 1H), 7.49 (dd, *J* = 9.2, 2.0 Hz, 1H), 7.56 (d, *J* = 9.2 Hz, 1H), 7.92 (d, *J* = 5.4 Hz, 1H), 8.83 (s, 1H) ppm.^13^C-NMR (101 MHz, CDCl_3_) δ 18.4 (CH), 115.4 (C), 123.5 (CH), 123.8 (C), 124.8 (CH), 125.5 (CH), 136.3 (CH), 137.5 (CH), 148.2 (C), 154.3 (C), and 157.7 (C) ppm. HRMS: calcd for C_11_H_9_N_2_OS [M + H]^+^ 217.0430, found 217.0435.

*7-Methoxy-10H-pyrido[1,2-a]thieno[3,2-d]pyrimidin-10-one***8d**: Yellow solid (32 mg, 43%), m.p. 183–185 °C. ^1^H-NMR (400 MHz, CDCl_3_) δ 3.94 (s, 3H), 7.39 (d, *J* = 5.4 Hz, 1H), 7.43 (dd, *J* = 9.7, 2.7 Hz, 1H), 7.57 (d, *J* = 9.7 Hz, 1H), 7.91 (d, *J* = 5.4 Hz, 1H), 8.49 (d, *J* = 2.7 Hz, 1H) ppm. ^13^C-NMR (101 MHz, CDCl_3_) δ 56.4 (CH), 105.4 (CH), 115.3 (C), 124.8 (CH), 126.8 (CH), 130.5 (CH), 135.9 (CH), 146.5 (C), 149.5 (C), 154.2 (C), and 157.2 (C) ppm. HRMS: calcd for C_11_H_9_N_2_O_2_S [M + H]^+^ 233.0379, found 233.0381.

*2-Phenyl-10H-pyrido[1,2-a]thieno[3,2-d]pyrimidin-10-one***9a**: White solid (40 mg, 67%), m.p. 210–212 °C. ^1^H-NMR (400 MHz, CDCl_3_) δ 7.00 (ddd, *J* = 7.6, 5.0, 2.8 Hz, 1H), 7.39–7.47 (m, 3H), 7.55 (s, 1H), 7.57–7.62 (m, 2H), 7.74 (d, *J* = 6.8 Hz, 2H), 9.00 (d, *J* = 7.3 Hz, 1H) ppm. ^13^C-NMR (101 MHz, CDCl_3_) δ 113.6 (CH), 114.8 (C), 120.0 (CH), 126.0 (CH), 126.4 (CH), 126.6 (2 × CH), 129.2 (2 × CH), 129.8 (CH), 133.1 (C), 134.3 (CH), 149.3 (C), 153.9 (C), 154.6 (C), and 158.5 (C) ppm. HRMS: calcd for C_16_H_11_N_2_OS [M + H]^+^ 279.0587, found 279.0587.

*7-Chloro-2-phenyl-10H-pyrido[1,2-a]thieno[3,2-d]pyrimidin-10-one***9b**: Yellow solid (44 mg, 65%), m.p. 230–232 °C. ^1^H-NMR (400 MHz, CDCl_3_) δ 7.43–7.49 (m, 3H), 7.54–7.56 (m, 3H), 7.75 (dd, *J* = 8.0, 1.4 Hz, 2H), 9.02–9.04 (m, 1H) ppm. ^13^C-NMR (101 MHz, CDCl_3_) δ 115.2 (C), 120.0 (CH), 122.2 (C), 124.1 (CH), 126.6 (2 × CH), 129.3 (2 × CH), 130.0 (CH), 133.0 (C), 135.6 (CH), 127.0 (CH), 147.6 (C), 153.0 (C), 155.0 (C), and 158.2 (C) ppm. HRMS: calcd for C_16_H_10_ClN_2_OS [M + H]^+^ 313.0197, found 313.0196.

*7-Methyl-2-phenyl-10H-pyrido[1,2-a]thieno[3,2-d]pyrimidin-10-one***9c**: White solid (44 mg, 70%), m.p. 197–199 °C. ^1^H-NMR (400 MHz, CDCl_3_) δ 2.42 (s, 3H), 7.42–7.51 (m, 4H), 7.55 (d, *J* = 7.5 Hz, 2H), 7.75–7.78 (m, 2H), 8.82–8.85 (m, 1H) ppm. ^13^C-NMR (101 MHz, CDCl_3_) δ 18.4 (CH), 114.8 (C), 120.0 (CH), 123.6 (CH), 123.7 (C), 125.5 (CH), 126.6 (2 × CH), 129.2 (2 × CH), 129.7 (CH), 133.3 (C), 137.5 (CH), 148.5 (C), 153.9 (C), 154.3 (C), and 158.4 (C) ppm. HRMS: calcd for C_17_H_13_N_2_OS [M + H]^+^ 293.0743, found 293.0745.

*7-Methoxy-2-phenyl-10H-pyrido[1,2-a]thieno[3,2-d]pyrimidin-10-one***9d**: Yellow solid (43 mg, 65%), m.p. 184–186 °C. ^1^H-NMR (400 MHz, CDCl_3_) δ 3.95 (s, 3H), 7.42–7.50 (m, 4H), 7.55–7.59 (m, 2H), 7.75–7.78 (m, 2H), 8.51 (d, *J* = 2.7 Hz, 1H) ppm.^13^C-NMR (101 MHz, CDCl_3_) δ 56.4 (CH), 105.6 (CH), 114.7 (C), 119.9 (CH), 126.6 (2 × CH), 126.8 (CH), 129.2 (2 × CH), 129.7 (CH), 130.5 (CH), 133.3 (C), 146.8 (C), 149.6 (C), 153.8 (C), 153.9 (C), and 157.8 (C) ppm. HRMS: calcd for C_17_H_13_N_2_O_2_S [M + H]^+^ 309.0692, found 309.0692.

### 3.4. General Procedure for the Synthesis of Compounds ***11**–**15a***, and ***11b***

A mixture of bromo compound (50 mg), amino derivative (2 eq.), Pd(OAc)_2_ (0.05 eq.), (±)-BINAP, 2,2′-Bis(diphenylphosphino)-1,1′-binaphthalene (0.1 eq.), K_2_CO_3_ (2 eq.) in CPME (1.5 mL) was stirred in MW at 110 °C. After completion the reaction was then cooled to room temperature and the mixture was concentrated under vacuum. The solid obtained was purified by flash chromatography using a mixture of ethyl acetate/petroleum ether

*Methyl 5-phenyl-3-(5-((phenylamino)methyl)-1H-1,2,3-triazol-1-yl)thiophene-2-carboxylate***11a***:* yellow oil (43 mg, 84%). ^1^H-NMR (400 MHz, CDCl_3_) δ 8.32 (s, 1H), 7.71 (s, 1H), 7.67–7.64 (m, 2H), 7.45 (d, *J* = 7.4 Hz, 3H), 7.20 (t, *J* = 7.8 Hz, 2H), 6.73 (t, *J* = 7.1 Hz, 3H), 4.57 (s, 2H), 3.81 (s, 3H) ppm. ^13^C-NMR (101 MHz, CDCl_3_) δ 160.7 (C), 149.3 (C), 147.5 (C), 145.4 (C), 138.7 (C), 132.1 (C), 129.8 (CH), 129.3 (2 × CH), 129.3 (2 × CH), 126.1 (2 × CH), 124.5 (CH), 122.0 (CH), 118.1 (CH), 113.2 (2 × CH), 100.0 (C), 52.5 (CH), and 39.8 (CH) ppm. HRMS: calcd for C_21_H_19_N_4_O_2_S [M + H]^+^ 391.1223, found 391.1224.

*Methyl 7-(5-((phenylamino)methyl)-1H-1,2,3-triazol-1-yl)-2,3-dihydrobenzo[b][1,4]dio-xine-6-carboxylate***12a**: orange oil (19 mg, 37%). ^1^H-NMR (400 MHz, CDCl_3_) δ 7.64 (s, 1H), 7.56 (s, 1H), 7.18 (t, *J* = 7.9 Hz, 2H), 6.94 (s, 1H), 6.71 (t, *J* = 8.1 Hz, 3H), 4.54 (s, 2H), 4.34 (d, *J* = 5.7 Hz, 5H), 3.57 (s, 3H) ppm.^13^C-NMR (101 MHz, CDCl_3_) δ 164.6 (C), 147.6 (C), 146.8 (C), 145.6 (C), 144.3 (C), 130.5 (C), 129.3 (2 × CH), 124.5 (CH), 120.5 (CH), 120.1 (C), 118.0 (CH), 116.7 (CH), 113.2 (2 × CH), 64.6 (CH), 64.3 (CH), 52.3 (CH), and 39.8 (CH) ppm. HRMS: calcd for C_19_H_19_N_4_O_4_ [M + H]^+^ 367.1401, found 367.1398.

*Methyl 6-(5-((phenylamino)methyl)-1H-1,2,3-triazol-1-yl)-1H-indazole-7-carboxylate***13a***:* orange solid (33 mg, 63%), m.p. 107–109 °C. ^1^H-NMR (400 MHz, CDCl_3_) δ 3.63 (s, 3H), 4.63 (s, 2H), 6.77 (dd, *J* = 12.0, 8.1 Hz, 3H), 7.27–7.20 (m, 3H), 7.76 (s, 1H), 8.08 (d, *J* = 8.2 Hz, 1H), 8.26 (s, 1H), 11.45 (s, 1H) ppm. ^13^C-NMR (101 MHz, CDCl_3_) δ 161.1 (C), 147.4 (C), 146.7 (C), 146.0 (C), 142.1 (C), 138.1 (C), 134.8 (CH), 130.2 (2 × CH), 126.4 (CH), 123.6 (CH), 119.6 (CH), 117.6 (CH), 116.3 (C), 113.3 (2 × CH), 52.2 (CH), and 40.4 (CH) ppm. HRMS: calcd for C_18_H_17_N_6_O_2_ [M + H]^+^ 349.1408, found 349.1405.

*Methyl 3-(5-((phenylamino)methyl)-1H-1,2,3-triazol-1-yl)thiophene-2-carboxylate***14a***:* yellow solid (23 mg, 44%), m.p. 99–101 °C. ^1^H-NMR (400 MHz, CDCl_3_) δ 8.26 (s, 1H), 7.60 (d, *J* = 5.4 Hz, 1H), 7.50 (d, *J* = 5.4 Hz, 1H), 7.19 (t, *J* = 7.9 Hz, 2H), 6.76–6.70 (m, 3H), 4.56 (s, 2H), 4.30 (s, 1H), 3.79 (s, 3H) ppm. ^13^C-NMR (101 MHz, CDCl_3_) δ 160.7 (C), 147.5 (C), 145.4 (C), 138.4 (C), 131.0 (CH), 129.3 (2 × CH), 126.7 (CH), 124.5 (CH), 122.0 (C), 118.1 (CH), 113.2 (2 × CH), 52.5 (CH), and 39.8 (CH) ppm. HRMS: calcd for C_15_H_15_N_4_O_2_S [M + H]^+^ 315.0910, found 315.0909.

*Methyl 4-(5-((phenylamino)methyl)-1H-1,2,3-triazol-1-yl)thiazole-5-carboxylate***15a***:* yellow oil (37 mg, 72%). ^1^H-NMR (400 MHz, CDCl_3_) δ 8.90 (s, 1H), 8.10 (s, 1H), 7.19 (t, *J* = 7.9 Hz, 2H), 6.72 (dd, *J* = 19.0, 7.9 Hz, 3H), 4.58 (s, 2H), 4.31 (s, 1H), 3.81 (s, 3H) ppm. ^13^C-NMR (101 MHz, CDCl_3_) δ 159.7 (C), 155.7 (CH), 147.7 (C), 147.4 (C), 145.8 (C), 129.3 (2 × CH), 123.5 (CH), 119.4 (C), 118.1 (CH), 113.2 (2 × CH), 53.1 (CH), and 39.8 (CH) ppm. HRMS: calcd for C_14_H_14_N_5_O_2_S [M + H]^+^ 316.0863, found 316.0862.

*Methyl3-(5-((benzofuran-5-ylamino)methyl)-1H-1,2,3-triazol-1-yl)-5-phenylthiophene-2-carboxylate***11b***:* orange oil (33 mg, 58%). ^1^H-NMR (400 MHz, CDCl_3_) δ 3.79 (s, 3H), 4.59 (s, 2H), 6.63 (s, 1H), 6.73 (dd, *J* = 8.8, 2.7 Hz, 1H), 6.88 (d, *J* = 2.7 Hz, 1H), 7.32 (d, *J* = 8.8 Hz, 1H), 7.43 (d, *J* = 7.2 Hz, 3H), 7.53 (s, 1H), 8.33 (s, 1H), 7.64 (d, *J* = 8.1 Hz, 2H), 7.71 (s, 1H) ppm. ^13^C-NMR (101 MHz, CDCl_3_) δ 40.8 (CH), 52.5 (CH), 103.5 (CH), 106.4 (CH), 111.8 (CH), 112.9 (CH), 119.7 (C), 122.0 (CH), 124.6 (CH), 126.1 (2 × CH), 128.2 (C), 129.3 (2 × CH), 129.8 (CH), 132.1 (C), 138.7 (C), 143.7 (C), 145.3 (CH), 145.5 (C), 149.3 (C), 149.3 (C), and 160.7 (C) ppm. HRMS: calcd for C_23_H_19_N_4_O_3_S [M + H]^+^ 431.1172, found 431.1170.

## 4. Conclusions

In summary, we have disclosed a synthesis of triazole scaffolds using greener alternatives as solvents. The conditions reported for all classes of heterocycles studied make this methodology an interesting alternative to conventional strategies, as it avoids the use of metal catalysts, several-step reactions, and conventional petroleum solvents.

We have also developed an efficient, environmentally sound microwave-assisted method for a Buchwald–Hartwig Coupling/Pyridine Dearomatization Sequence. Applying CPME and 2-MeTHF as solvents, from methyl 3-aminothiophene-2-carboxylate and 3-amino-5-phenylthiophene-2-carboxylate, the final compounds were obtained with interesting yields.

In the last part of this manuscript, we demonstrated a Buchwald–Hartwig amination of bromo derivatives synthesized at the beginning of this work. Once again, we were able to successfully find an approach making use of microwave heating that was compatible with a green solvent. In this case, this achievement allowed a threefold improvement: shortening of the usual reaction time, CPME as an alternative to the solvents generally used in this type of reaction (DMF, for example), and good yields.

## Figures and Tables

**Table 1:** Synthesis of **2a** using several green solvents.

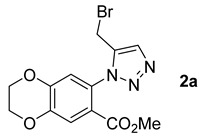		
Entry	Solvent	Yield ^a^ (%)
1	CPME	40
2	2-MeTHF	70
3	Eucalyptol	54

^a^ Isolated yield after purification by flash chromatography.

**Table 2:** Synthesis of **3a** using several green solvents.

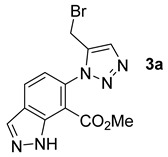		
Entry	Solvent	Yield ^a^ (%)
1	CPME	33
2	2-MeTHF	41
3	Eucalyptol	37

^a^ Isolated yield after purification by flash chromatography.

**Table 3:** Synthesis of **4a** using several green solvents.

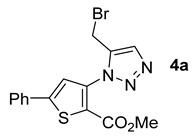		
Entry	Solvent	Yield ^a^ (%)
1	CPME	95
2	2-MeTHF	89
3	Eucalyptol	87
4 ^b^	CPME	28

^a^ Isolated yield after purification by flash chromatography. ^b^ Microwave, 140 °C, 1 h.

**Table 4:** Synthesis of **5a** using several green solvents.

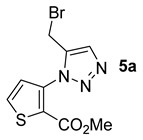		
Entry	Solvent	Yield ^a^ (%)
1	CPME	74
2	2-MeTHF	71
3	Eucalyptol	95
4 ^b^	Eucalyptol	40

^a^ Isolated yield after purification by flash chromatography. ^b^ MW, 140 °C, 1 h.

**Table 5:** Synthesis of **6a** using several green solvents.

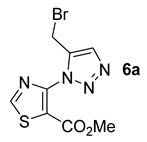		
Entry	Solvent	Yield ^a^ (%)
1	CPME	29
2	2-MeTHF	12
3	Eucalyptol	18

^a^ Isolated yield after purification by flash chromatography.

**Table 6:** Synthesis of **8a** varying the solvent and heating system.

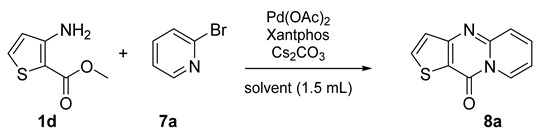						
Entry	Solvent	T (°C)	t (h)	Catalyst% Pd(OAc)_2_/Xantphos	Heating System	Yield ^a^ (%)
1	CPME	120	overnight	3/4	Classical	82
2	2-MeTHF	120	overnight	3/4	Classical	89
3	Toluene	120	overnight	3/4	Classical	86
4	CPME	140	1	3/4	MW	55
5	2-MeTHF	140	1	3/4	MW	65
6	Toluene	140	1	3/4	MW	72
7	CPME	140	2	3/4	MW	55
8	2-MeTHF	140	2	3/4	MW	65
9	CPME	160	1	3/4	MW	71
10	2-MeTHF	160	1	3/4	MW	64

^a^ Isolated yield after purification by flash chromatography.

**Table 7:** Synthesis of **9a** varying the solvent and heating system.

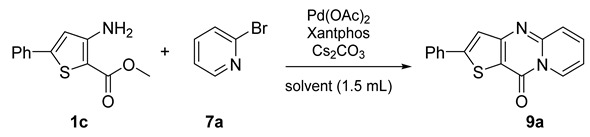						
Entry	Solvent	T (°C)	t (h)	Catalyst% Pd(OAc)_2_/Xantphos	Heating System	Yield ^a^ (%)
1	CPME	120	overnight	3/4	Classical	96
2	2-MeTHF	120	overnight	3/4	Classical	86
3	Toluene	120	overnight	3/4	Classical	86
4	Toluene	140	1	3/4	MW	39
5	Toluene	140	2	3/4	MW	43
6	2-MeTHF	140	2	3/4	MW	19
7	2-MeTHF	140	4	3/4	MW	35
8	CPME	160	1	3/4	MW	53
9	CPME	160	2	3/4	MW	87

^a^ Isolated yield after purification by flash chromatography.

**Table 8:** Synthesis optimization of **11a** by varying the solvent, base, and heating system.

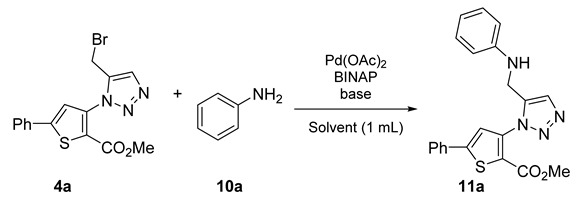						
Entry	Solvent	T (°C)	t	Catalyst% Pd(OAc)_2_/BINAP	Base	Yield ^a^ (%)
1	Toluene	110	22 h	5/10	Cs_2_CO_3_	45
2	DMF	r.t	17 h	5/10	K_2_CO_3_	84
3	DMF	r.t	17 h	5/10	Cs_2_CO_3_	54
4	Toluene	r.t	18 h	5/10	K_2_CO_3_	76
5	2-MeTHF	r.t	5 days	5/10	K_2_CO_3_	46
6	CPME	r.t	3 days	5/10	K_2_CO_3_	70
7	CPME ^b^	110	1 h	5/10	K_2_CO_3_	84

^a^ Isolated yield after purification by flash chromatography. ^b^ MW. BINAP: (±)-BINAP, 2,2′-Bis(diphenylphosphino)-1,1′-binaphthalene; DMF: *N,N*-Dimethylformamide.

## Data Availability

The data presented in this study are available in Supplementary Materials.

## Supplementary Materials

The following are available online: ^1^H-NMR and ^13^C-NMR Spectra of all Products.

## Sample Availability

Samples of all compounds used are available from the authors.

## References

[1] Clarke C.J., Tu W.-C., Levers O., Bröhl A., Hallett J.P. (2018). Green and Sustainable Solvents in Chemical Processes. Chem. Rev..

[2] Laird T. (2012). Green Chemistry is Good Process Chemistry. Org. Process Res. Dev..

[3] Henderson R.K., Jiménez-González C., Constable D.J.C., Alston S.R., Inglis G.G.A., Fisher G., Sherwood J., Binks S.P., Curzons A.D. (2011). Expanding GSK’s solvent selection guide—embedding sustainability into solvent selection starting at medicinal chemistry. Green Chem..

[4] Capello C., Fischer U., Hungerbühler K. (2007). What is a green solvent? A comprehensive framework for the environmental assessment of solvents. Green Chem..

[5] Alfonsi K., Colberg J., Dunn P.J., Fevig T., Jennings S., Johnson T.A., Kleine H.P., Knight C., Nagy M.A., Perry D.A. (2008). Green chemistry tools to influence a medicinal chemistry and research chemistry based organization. Green Chem..

[6] Prat D., Pardigon O., Flemming H.W., Letestu S., Ducandas V., Isnard P., Guntrum E., Senac T., Ruisseau S., Cruciani P. (2013). Sanofi’s Solvent Selection Guide: A Step Toward More Sustainable Processes. Org. Process Res. Dev..

[7] Diorazio L.J., Hose D.R.J., Adlington N.K. (2016). Toward a More Holistic Framework for Solvent Selection. Org. Process Res. Dev..

[8] Prat D., Wells A., Hayler J., Sneddon H., McElroy C.R., Abou-Shehada S., Dunn P.J. (2016). CHEM21 selection guide of classical- and less classical-solvents. Green Chem..

[9] Alder C.M., Hayler J.D., Henderson R.K., Redman A.M., Shukla L., Shuster L.E., Sneddon H.F. (2016). Updating and further expanding GSK’s solvent sustainability guide. Green Chem..

[10] Campos J.F., Pacheco-Benichou A., Fruit C., Besson T., Berteina-Raboin S. (2020). Synthesis of Benzo-Fused 11H-Pyrido[2,1-b]quinazolin-11-ones by a Buchwald–Hartwig Coupling/Pyridine Dearomatization Sequence in Eucalyptol. Synthesis.

[11] Campos J.F., Berteina-Raboin S. (2020). Eucalyptol as bio-based solvent for Migita-Kosugi-Stille coupling reaction on O,S,N-Heterocycles. Catal. Today.

[12] Campos J.F., Berteina-Raboin S. (2019). Eucalyptol as a Bio-Based Solvent for Buchwald-Hartwig Reaction on O,S,N-Heterocycles. Catalysts.

[13] Campos J.F., Scherrmann M.-C., Berteina-Raboin S. (2019). Eucalyptol: A new solvent for the synthesis of heterocycles containing oxygen, sulfur and nitrogen. Green Chem..

[14] Campos J.F., Loubidi M., Scherrmann M.-C., Berteina-Raboin S. (2018). A greener and efficient method for nucleophilic aromatic substitution of nitrogen-containing fused heterocycles. Molecules.

[15] Taylor R.D., MacCoss M., Lawson A.D.G. (2014). Rings in Drugs. J. Med. Chem..

[16] Vitaku E., Smith D.T., Njardarson J.T. (2014). Analysis of the Structural Diversity, Substitution Patterns, and Frequency of Nitrogen Heterocycles among U.S. FDA Approved Pharmaceuticals. J. Med. Chem..

[17] Monguchi Y., Kitamoto K., Ikawa T., Maegawa T., Sajiki H. (2008). Evaluation of Aromatic Amination Catalyzed by Palladium on Carbon: A Practical Synthesis of Triarylamines. Adv. Synth. Catal..

[18] Campos J.F., Queiroz M.-J.R.P., Berteina-Raboin S. (2018). Synthesis of New Annulated Pyrazinothienotriazolopyrimidinones and Triazolylthienopyrazines. Synthesis.

[19] Kannadasan S., Srinivasan P.C. (2004). A Facile Synthesis of 3,4-Benzo-β-carbolines. Synth Commun..

[20] Pippione A.C., Sainas S., Goyal P., Fritzson I., Cassiano G.C., Giraudo A., Giorgis M., Tavella T.A., Bagnati R., Rolando B. (2019). Hydroxyazole scaffold-based Plasmodium falciparum dihydroorotate dehydrogenase inhibitors: Synthesis, biological evaluation and X-ray structural studies. Eur. J. Med. Chem..

