# Supplementary material for: Demonstration of Green Solvent Performance on O,S,N-Heterocycles Synthesis: Metal-Free Click Chemistry and Buchwald—Hartwig Coupling
Source: Molecules. 2021 Feb 18;26(4):1074. doi: 10.3390/molecules26041074 (PMC7922032; doi:10.3390/molecules26041074)
Supplement: Supplementary file 1 [file molecules-26-01074-s001.pdf]

molecules

MDPI

Article

Demonstration of Green Solvent Performance on O,S,N-Heterocycles Synthesis: Metal-Free Click Chemistry and Buchwald–Hartwig Coupling

Joana F. Campos [1], Manon Cailler [1], Remi Claudel [1], Benjamin Prot [1], Thierry Besson [2] and Sabine Berteina-Raboin [1,]*

[1] Institut de Chimie Organique et Analytique (ICOA), Université d'Orléans UMR-CNRS 7311, BP 6759, rue de Chartres, 45067 Orléans CEDEX 2, France; joana-filomena.mimoso-silva-de-campos@univ-orleans.fr (J.F.C.); manon.cailler@etu.univ-orleans.fr (M.C.); remi.claudel@etu.univ-orleans.fr (R.C.); benjamin.prot@etu.univ-orleans.fr (B.P.)

[2] Normandie Univ, UNIROUEN, INSA Rouen, CNRS, COBRA UMR 6014, 76000 Rouen, France; thierry.besson@univ-rouen.fr (T.B.)

* Correspondence: sabine.berteina-raboin@univ-orleans.fr; Tel.: +33-238-494-856

Supplementary Material

^{1}H NMR and ^{13}C NMR Spectra of all Products

methyl 7-(5-(bromomethyl)-1H-1,2,3-triazol-1-yl)-2,3-dihydrobenzo[b][1,4]dioxine-6-carboxylate

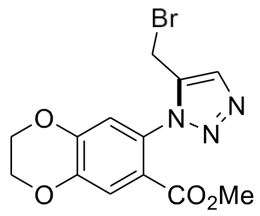

2a

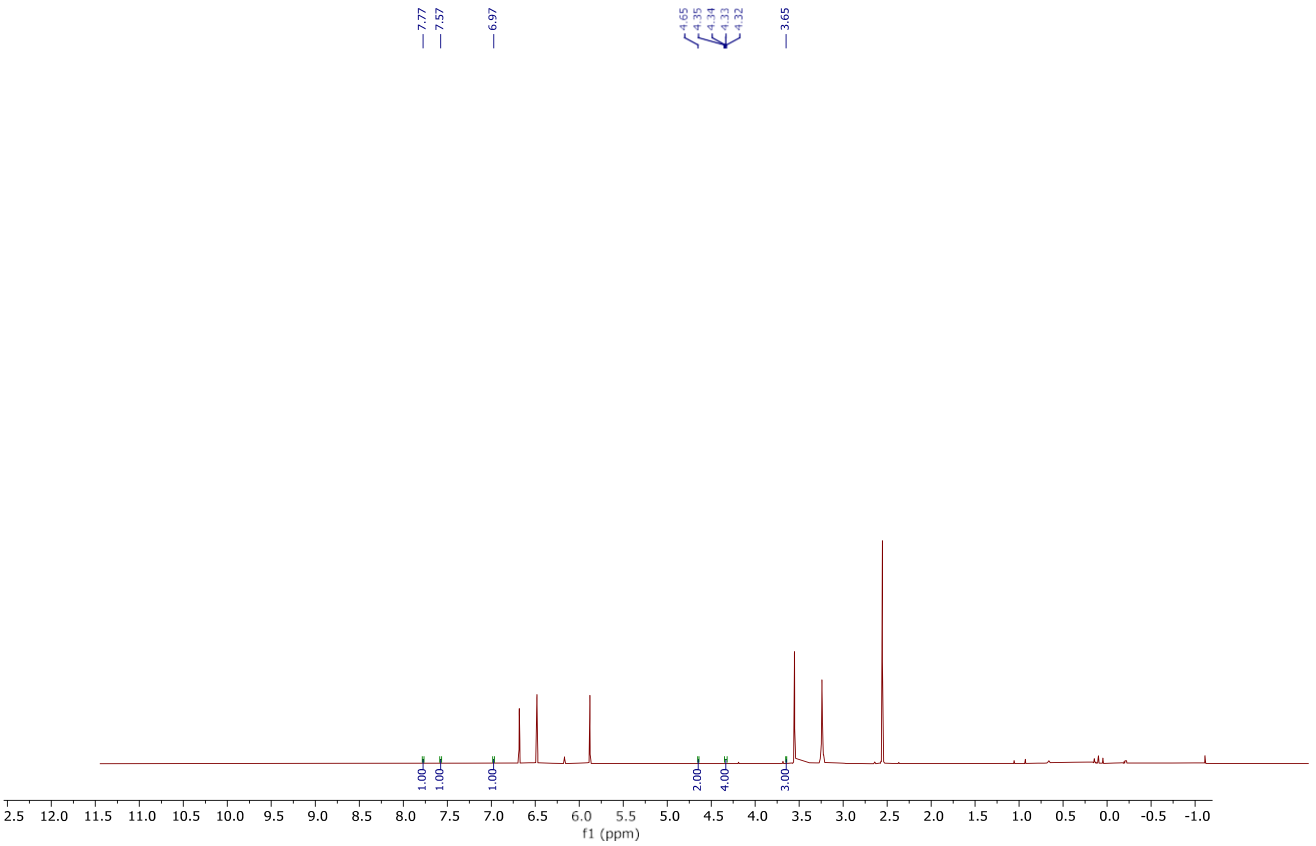

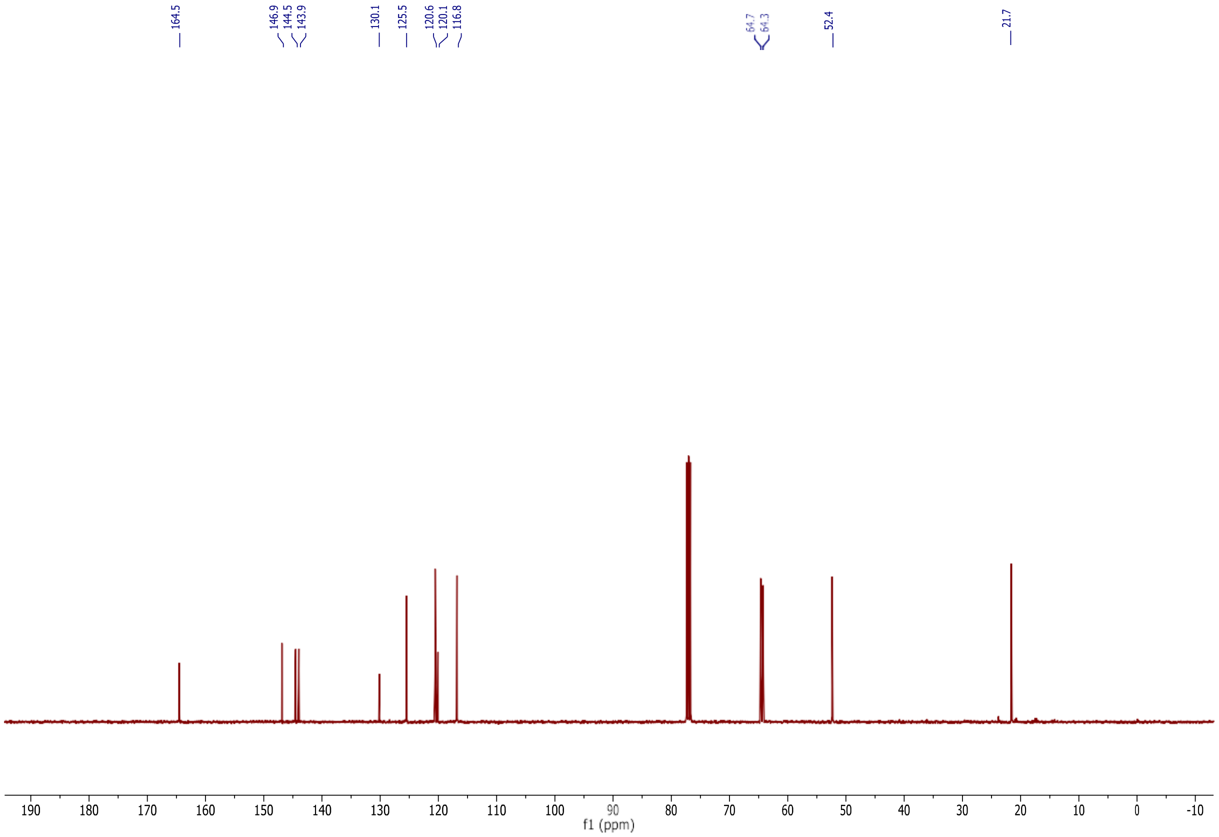
164.5
146.9
144.5
143.9
130.1
125.5
120.6
120.1
116.8
64.7
64.3
52.4
21.7
190 180 170 160 150 140 130 120 110 100 90 80 70 60 50 40 30 20 10 0 -10
f1 (ppm)

methyl 7-(5-(hydroxymethyl)-1H-1,2,3-triazol-1-yl)-2,3-dihydrobenzo[b][1,4]dioxine-6-carboxylate **2b**

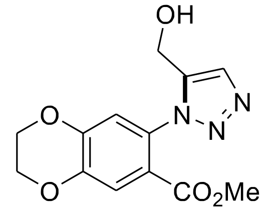

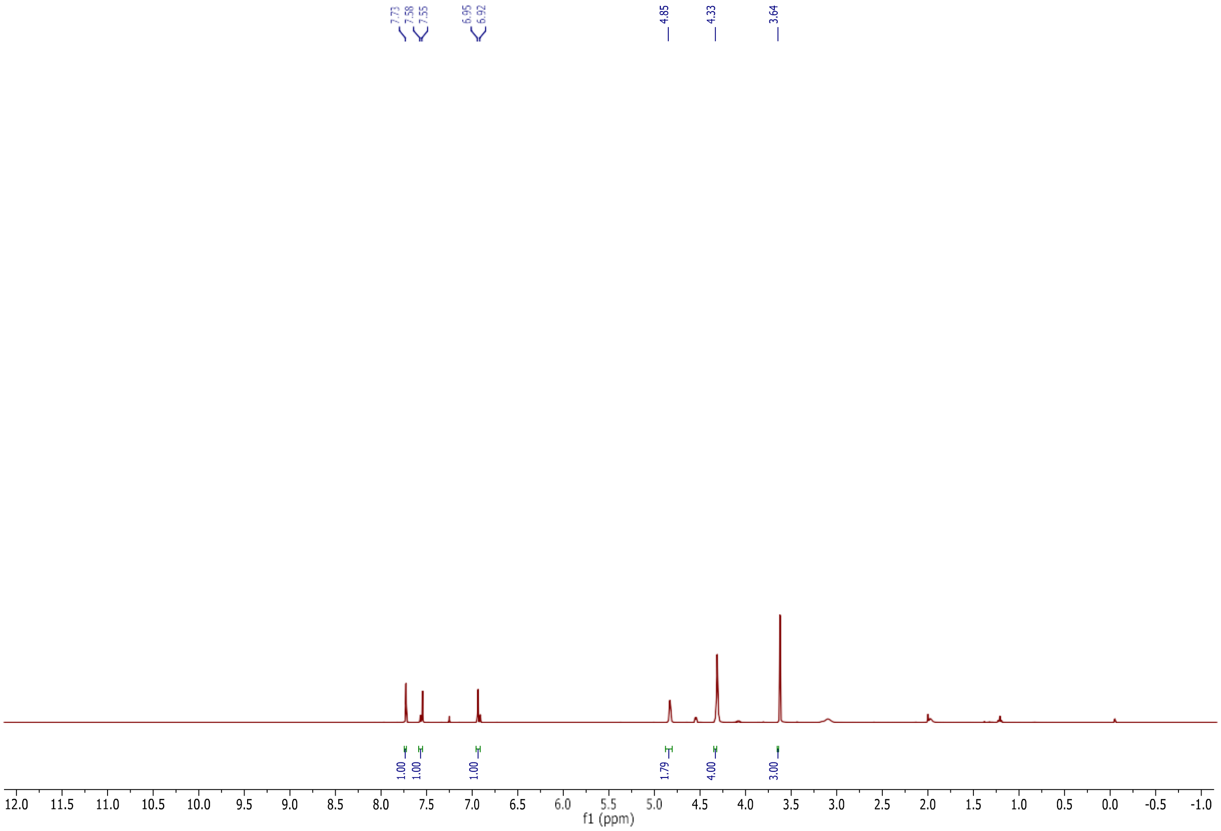

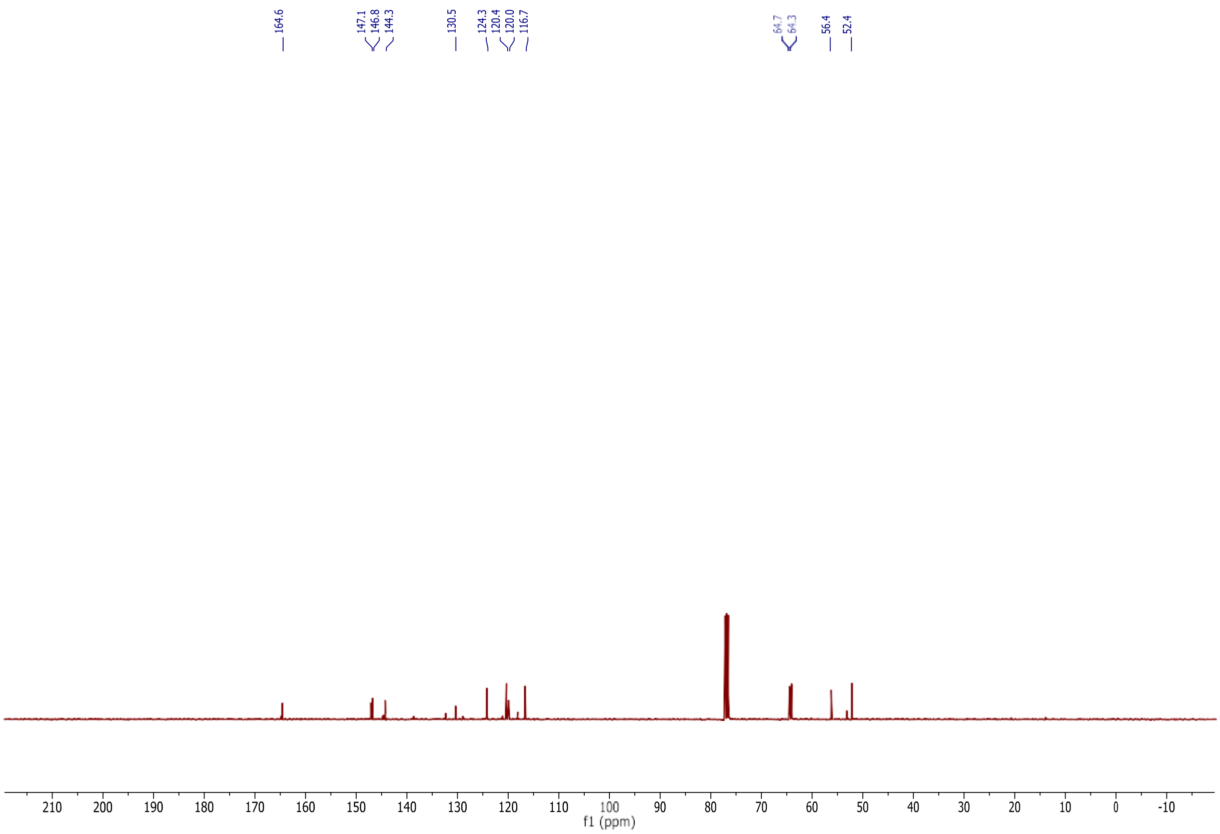
164.6
147.1
146.8
144.3
130.5
124.3
120.4
120.0
116.7
64.7
64.3
56.4
52.4
210 200 190 180 170 160 150 140 130 120 110 100 90 80 70 60 50 40 30 20 10 0 -10
f1 (ppm)

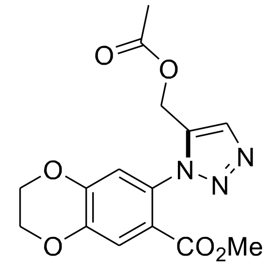

methyl 7-(5-(acetoxymethyl)-1H-1,2,3-triazol-1-yl)-2,3-dihydrobenzo[b][1,4]dioxine-6-carboxylate ***2c***

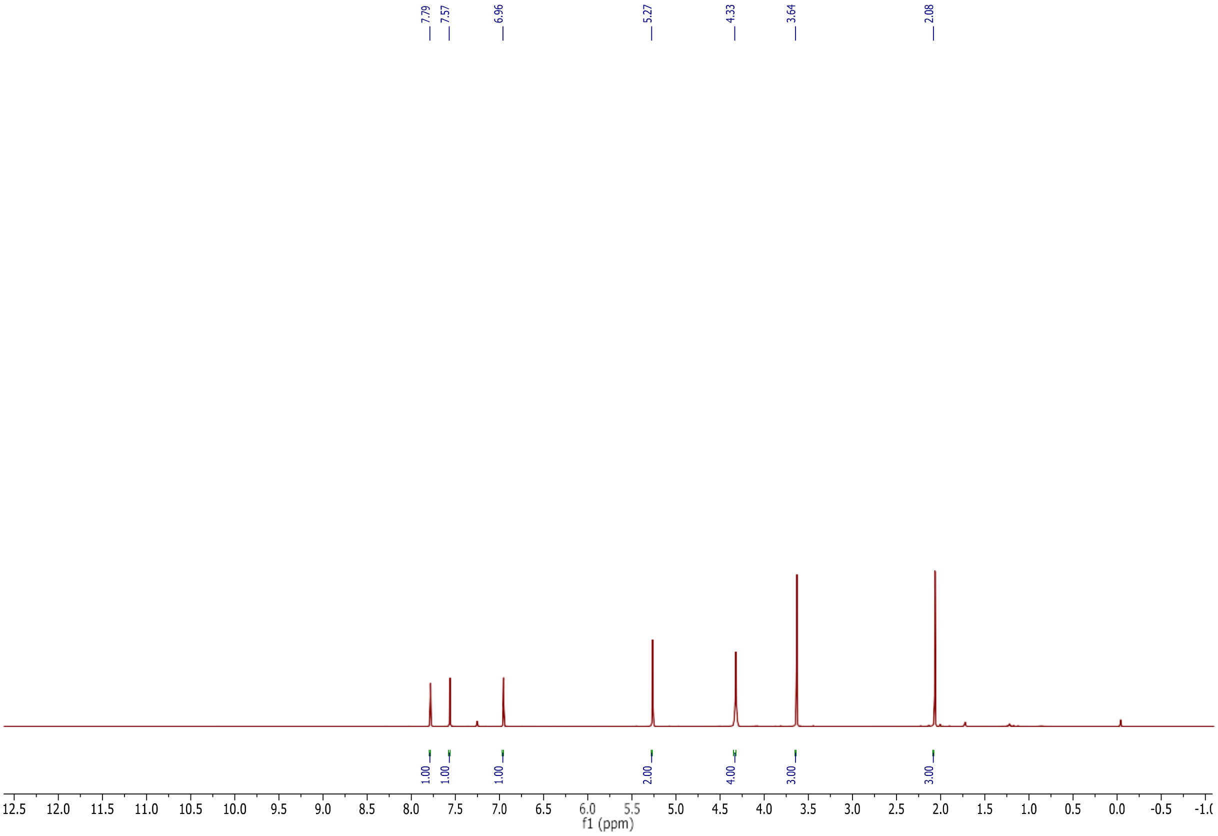

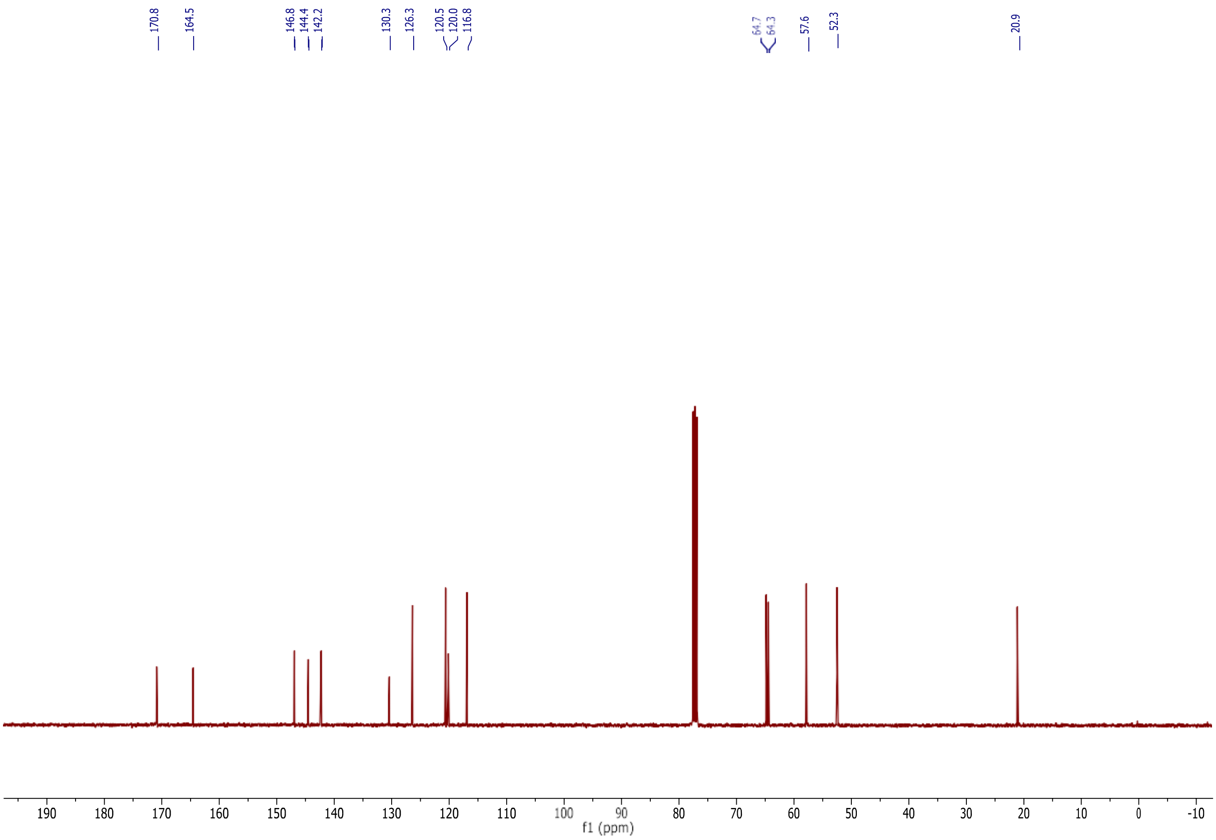

170.8
164.5
146.8
144.4
142.2
130.3
126.3
120.5
120.0
116.8
64.7
64.3
57.6
52.3
20.9
190
180
170
160
150
140
130
120
110
100
90
80
70
60
50
40
30
20
10
0
-10
f1 (ppm)

methyl 7-(5-((benzyl(methyl)amino)methyl)-1H-1,2,3-triazol-1-yl)-2,3-dihydrobenzo[b][1,4]dioxine-6-carboxylate

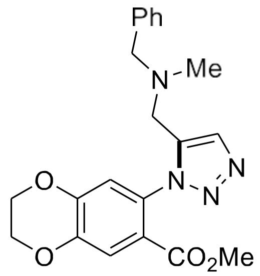

2d

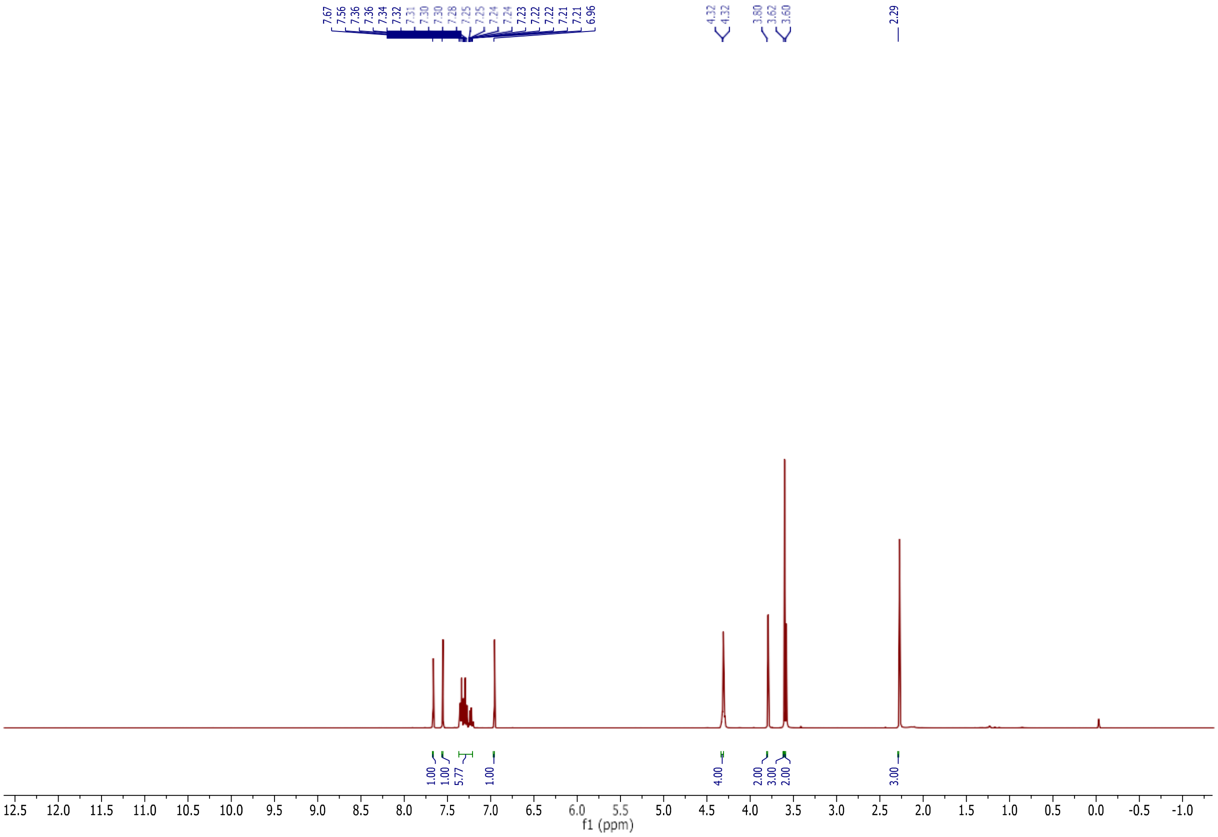

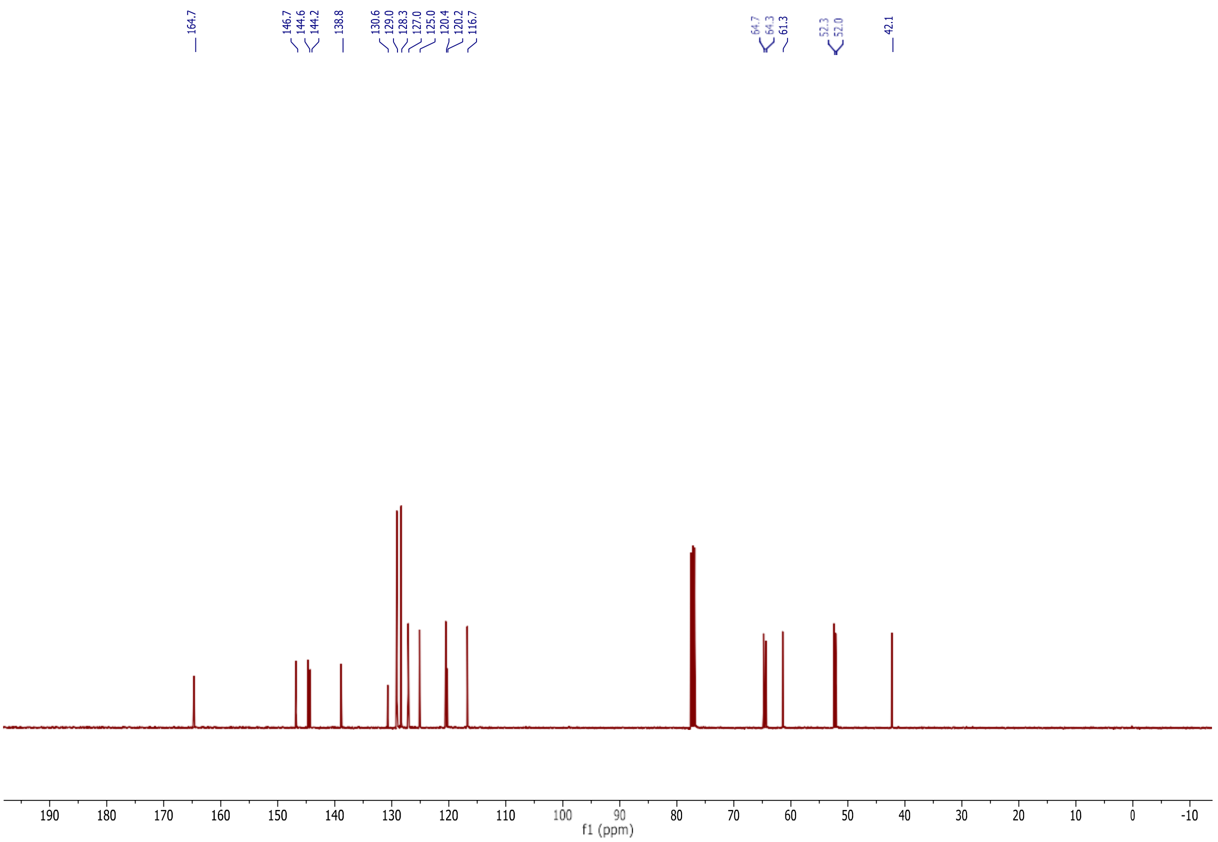
164.7
146.7
144.6
144.2
138.8
130.6
129.0
128.3
127.0
125.0
120.4
120.2
116.7
64.7
64.3
61.3
52.3
52.0
42.1
190 180 170 160 150 140 130 120 110 100 90 80 70 60 50 40 30 20 10 0 -10
f1 (ppm)

methyl 6-(5-(bromomethyl)-1H-1,2,3-triazol-1-yl)-1H-indazole-7-carboxylate

3a

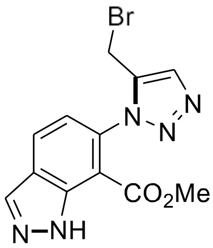

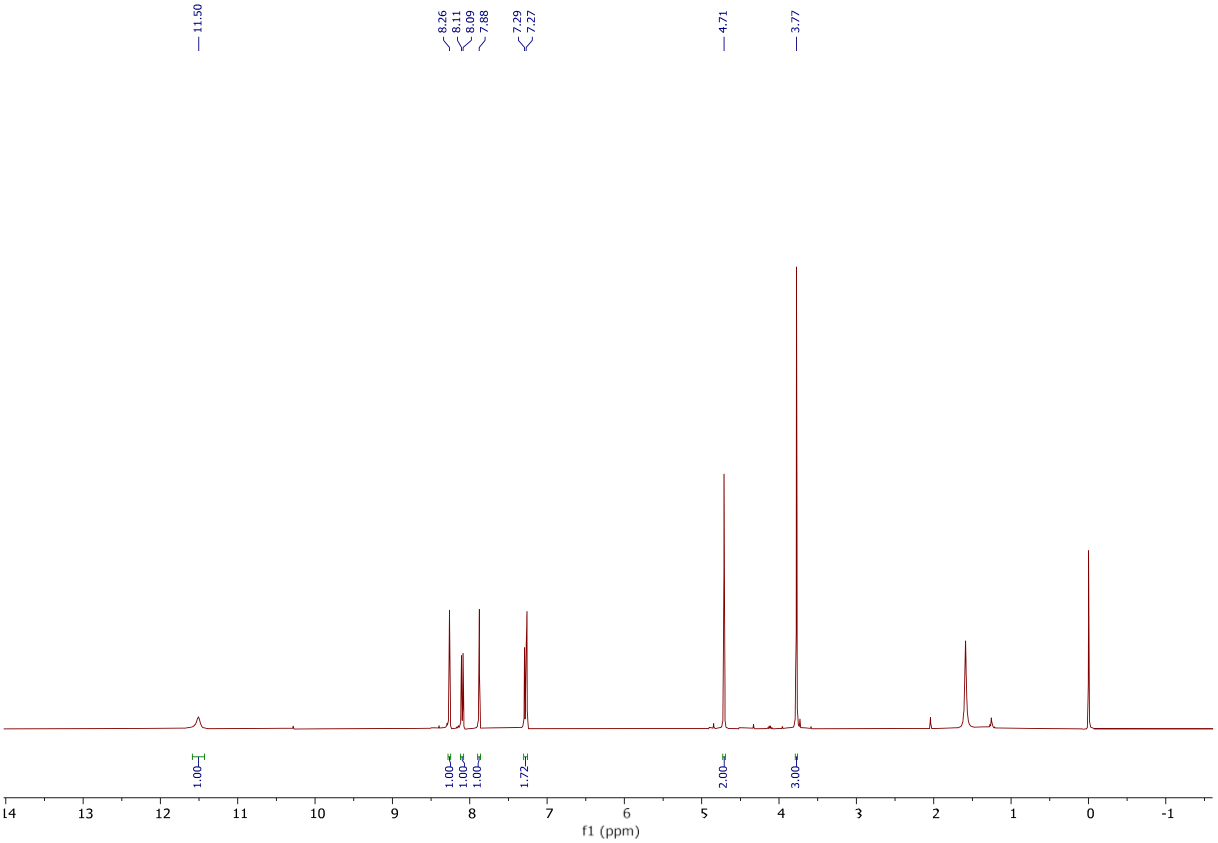

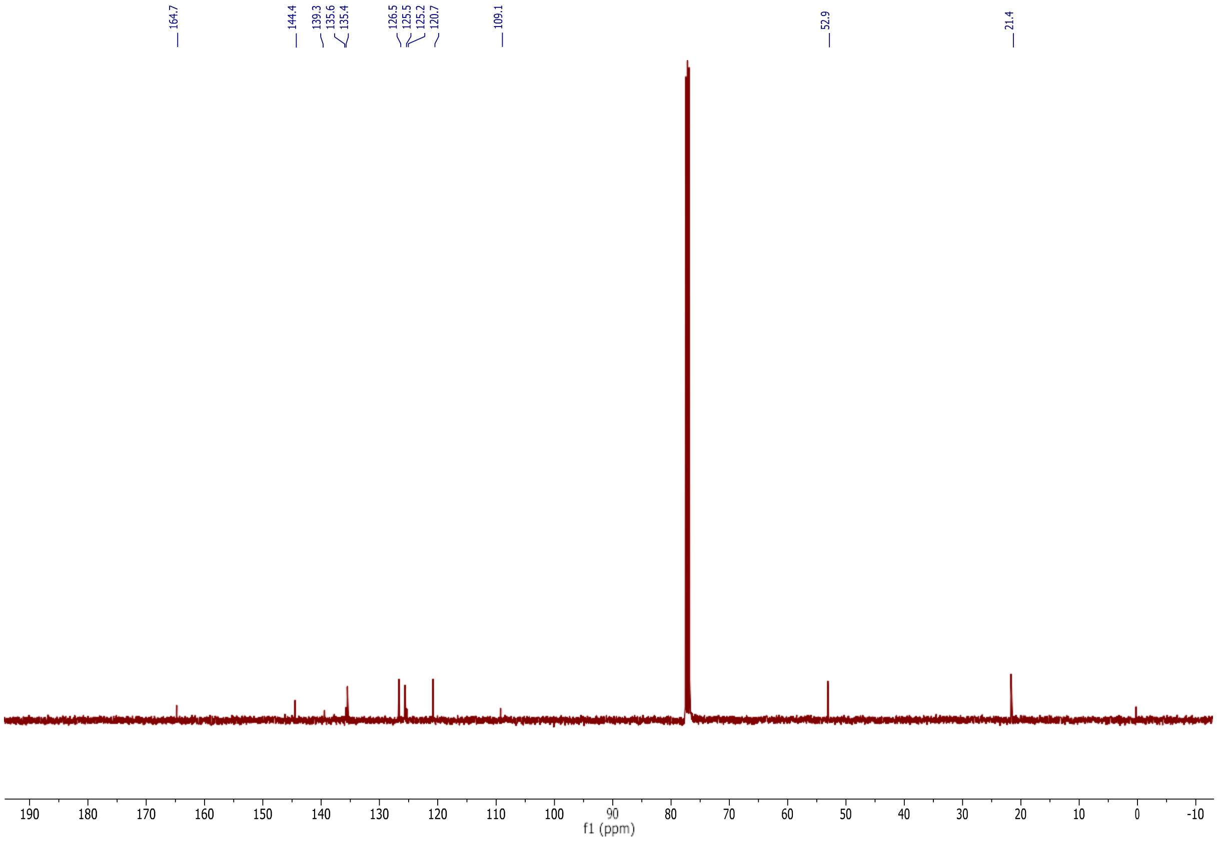
164.7
144.4
139.3
135.6
135.4
126.5
125.5
125.2
120.7
109.1
52.9
21.4
190
180
170
160
150
140
130
120
110
100
90
80
70
60
50
40
30
20
10
0
-10
f1 (ppm)

methyl 6-(5-(hydroxymethyl)-1H-1,2,3-triazol-1-yl)-1H-indazole-7-carboxylate

3b

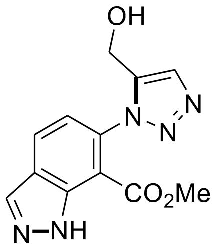

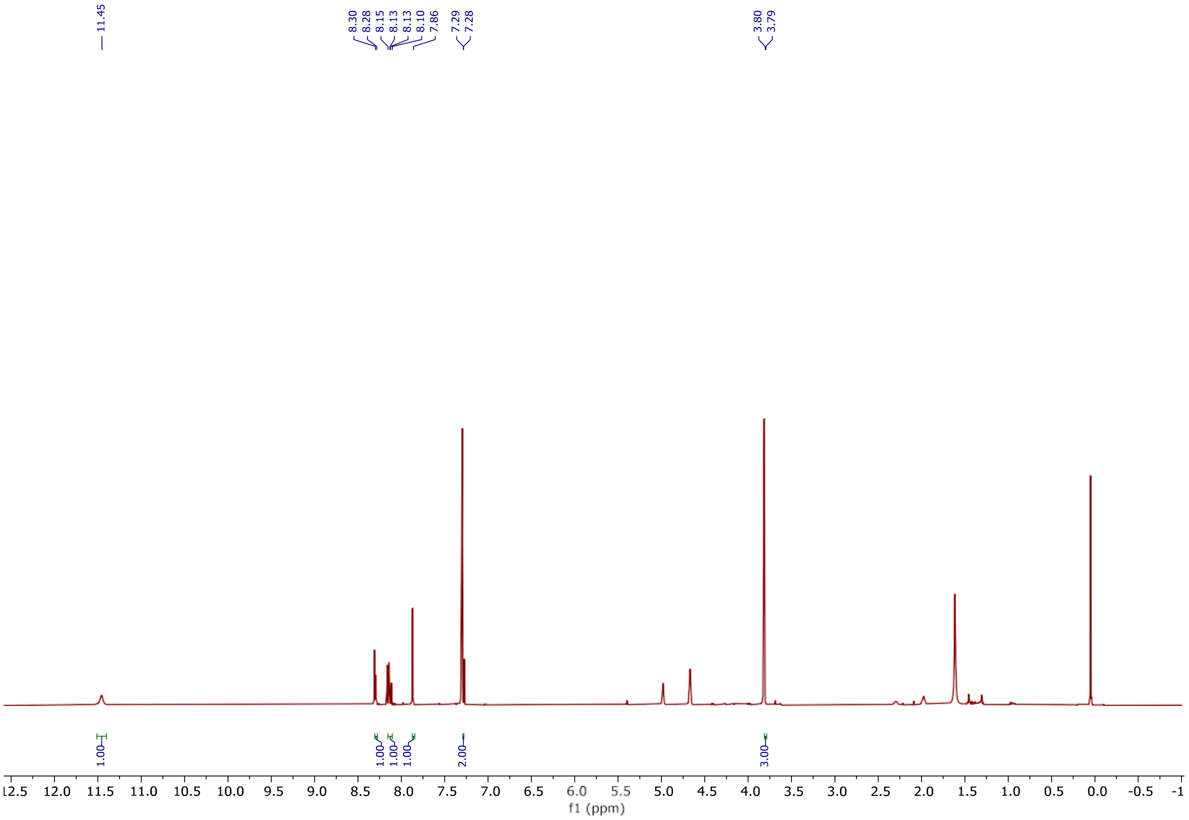

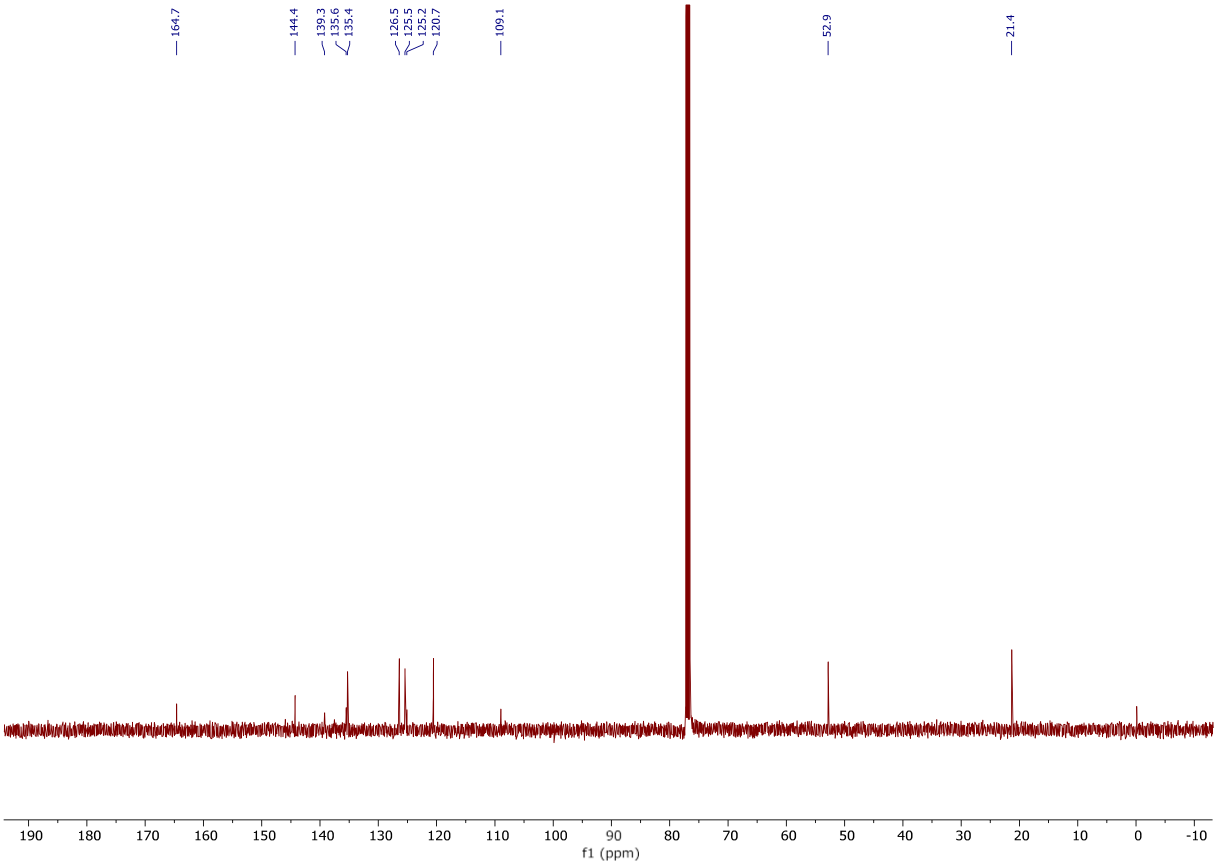

164.7
144.4
139.3
135.6
135.4
126.5
125.5
125.2
120.7
109.1
52.9
21.4
190
180
170
160
150
140
130
120
110
100
90
80
70
60
50
40
30
20
10
0
-10
f1 (ppm)

methyl 6-(5-(acetoxymethyl)-1H-1,2,3-triazol-1-yl)-1H-indazole-7-carboxylate

3c

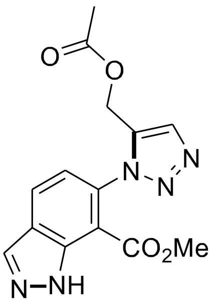

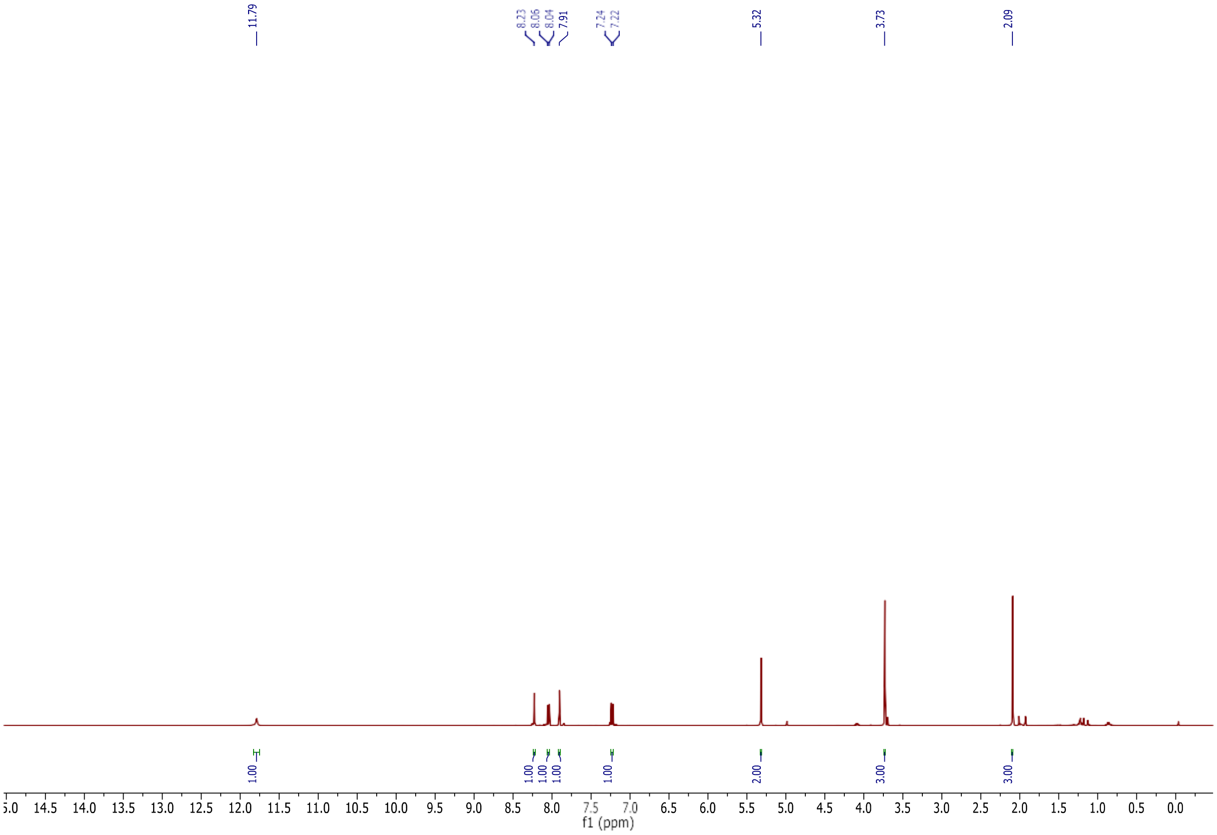

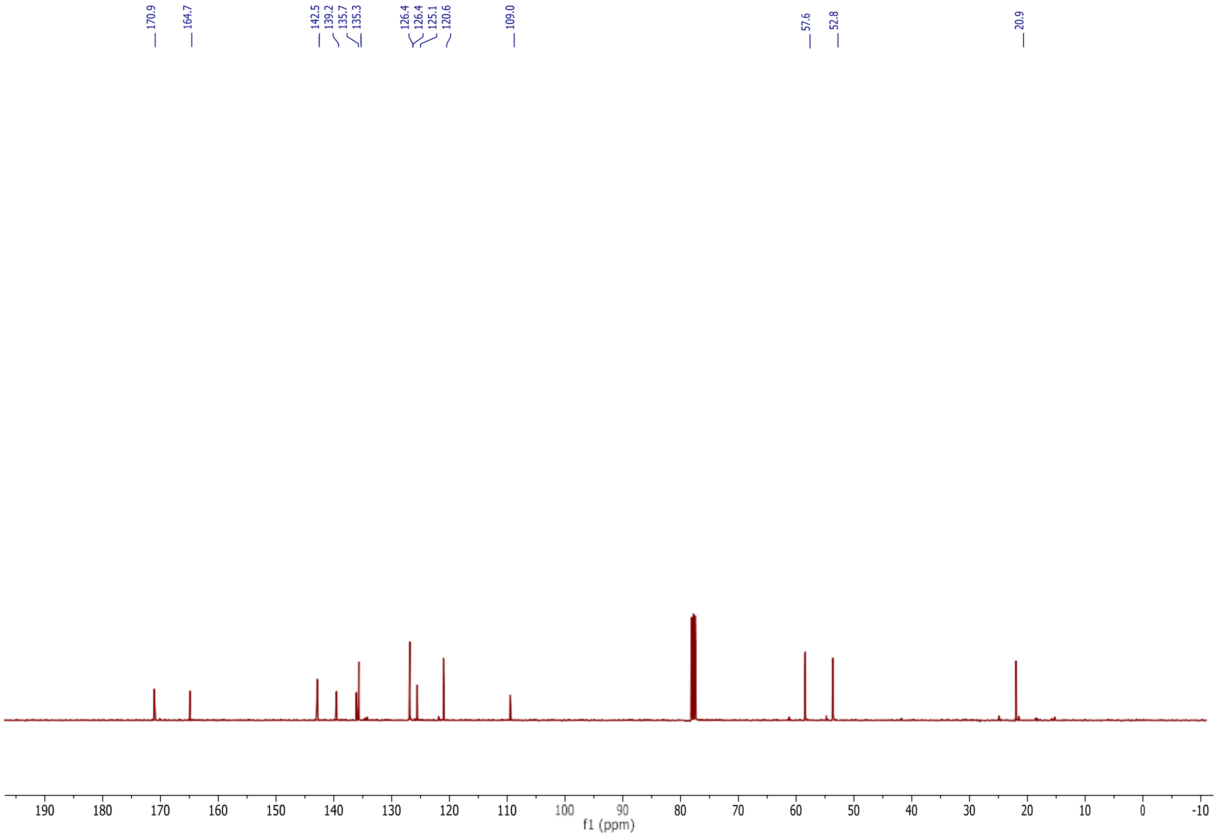

170.9
164.7
142.5
139.2
135.7
135.3
126.4
126.4
125.1
120.6
109.0
57.6
52.8
20.9
190 180 170 160 150 140 130 120 110 100 90 80 70 60 50 40 30 20 10 0 -10
f1 (ppm)

methyl 6-(5-((benzyl(methyl)amino)methyl)-1H-1,2,3-triazol-1-yl)-1H-indazole-7-carboxylate

3d

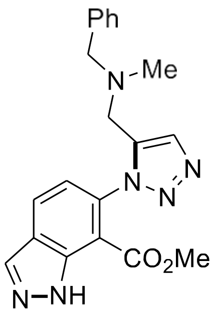

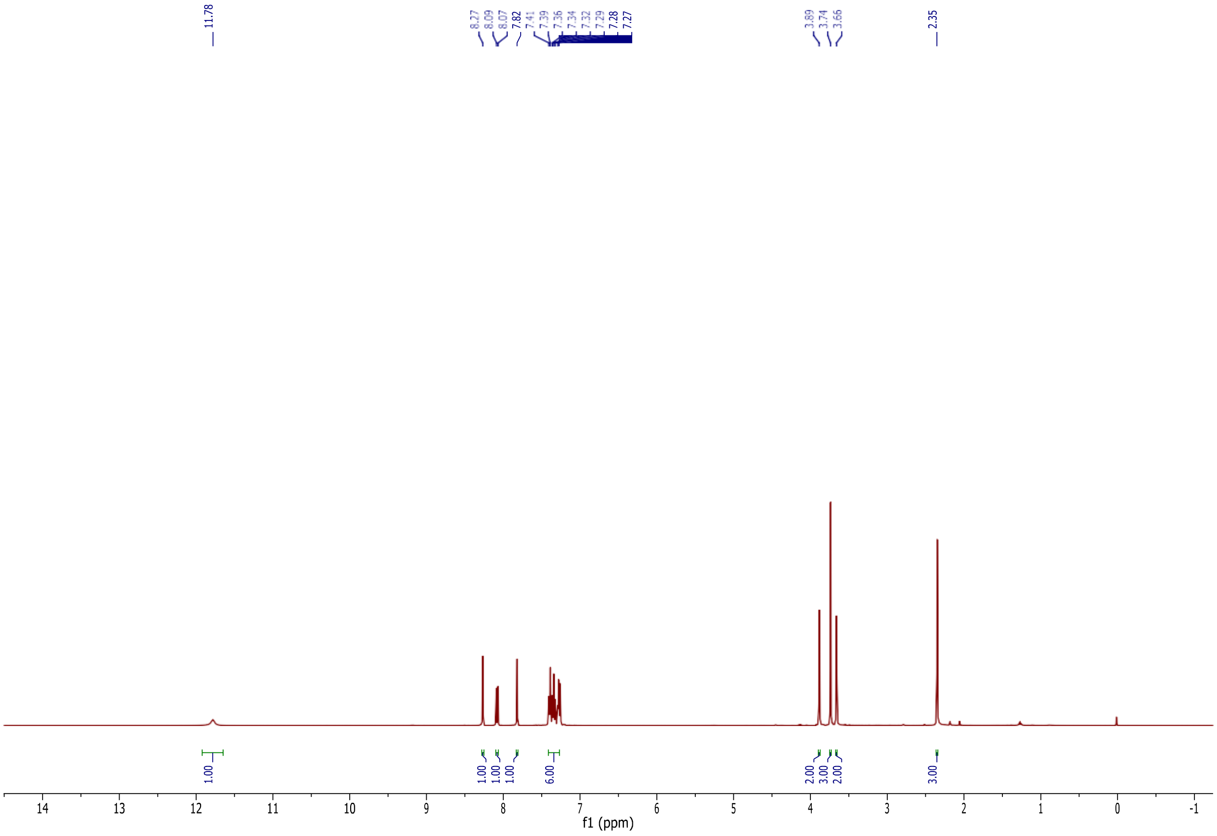

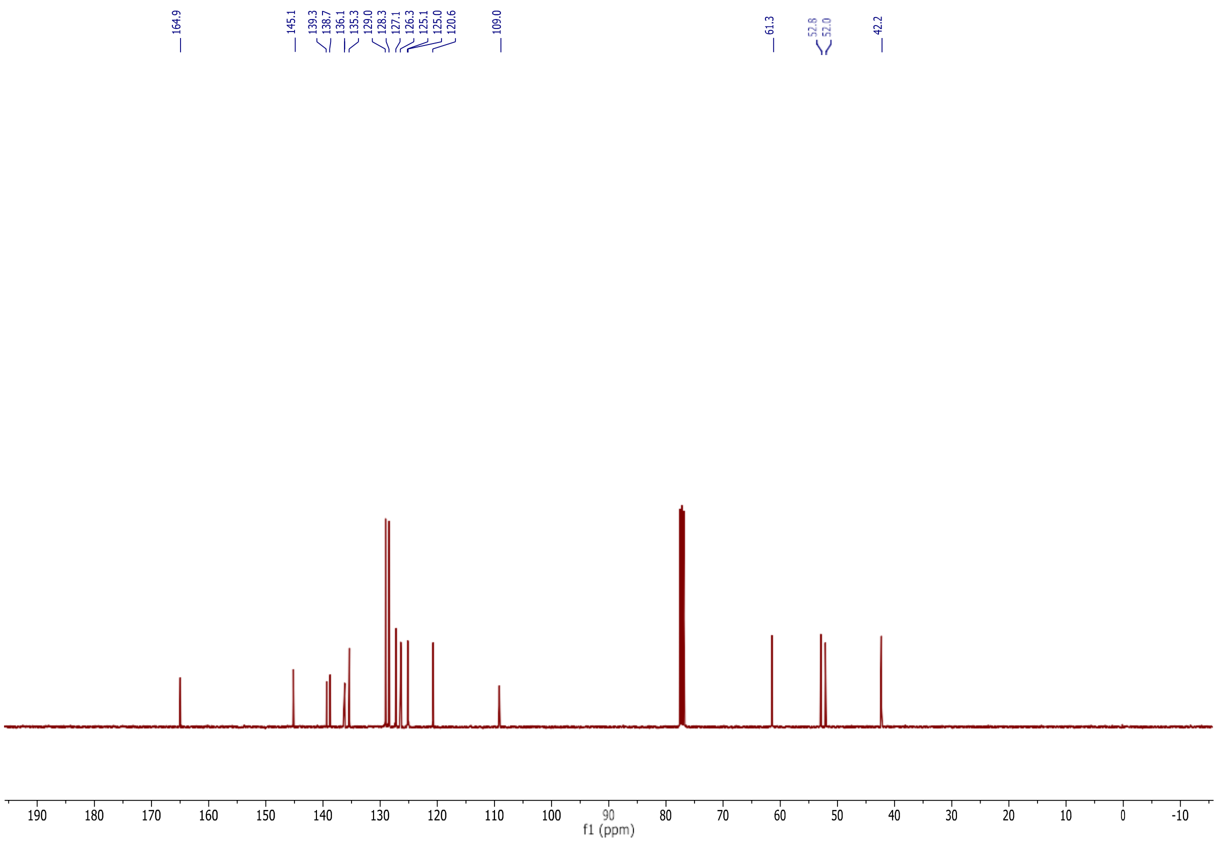
164.9
145.1
139.3
138.7
136.1
135.3
129.0
128.3
127.1
126.3
125.1
125.0
120.6
109.0
61.3
52.8
52.0
42.2
190
180
170
160
150
140
130
120
110
100
90
80
70
60
50
40
30
20
10
0
-10
f1 (ppm)

methyl 3-(5-(bromomethyl)-1H-1,2,3-triazol-1-yl)-5-phenylthiophene-2-carboxylate

4a

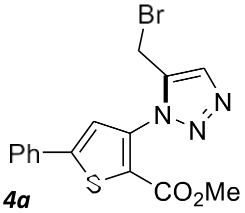

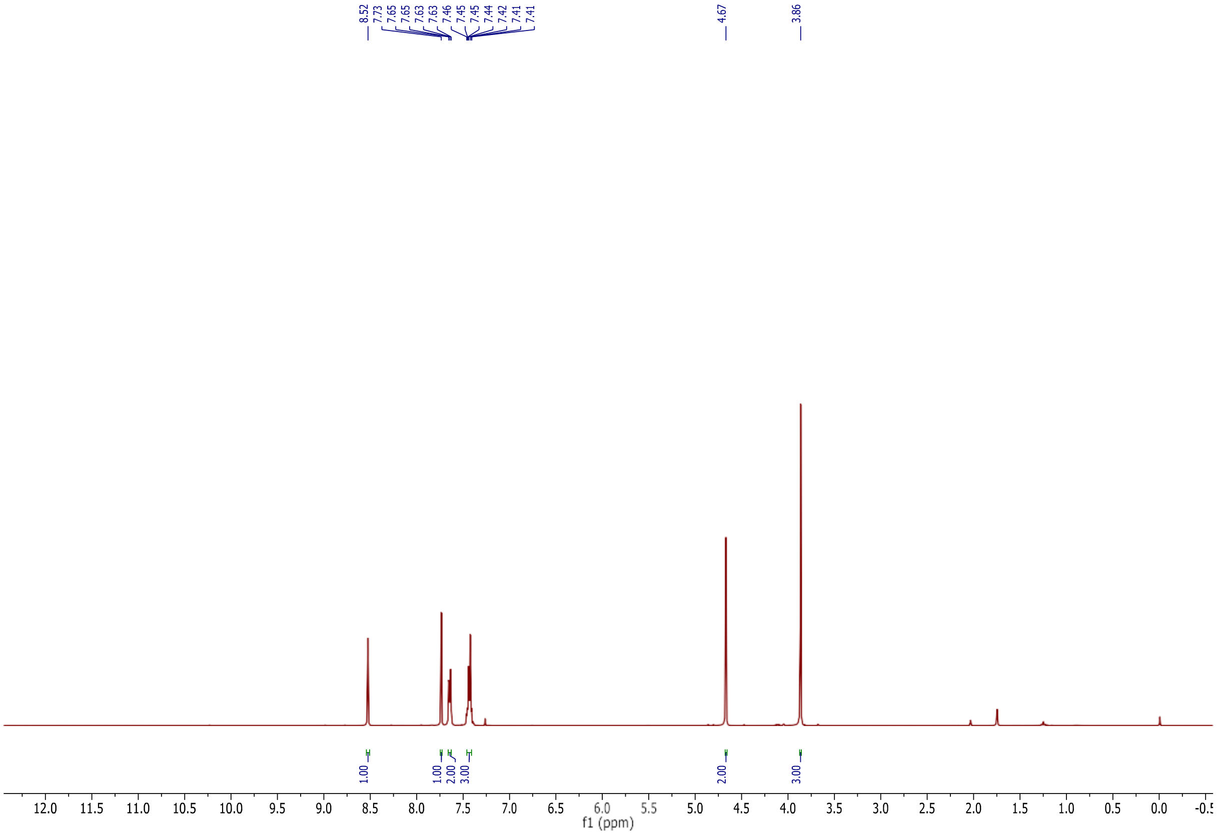

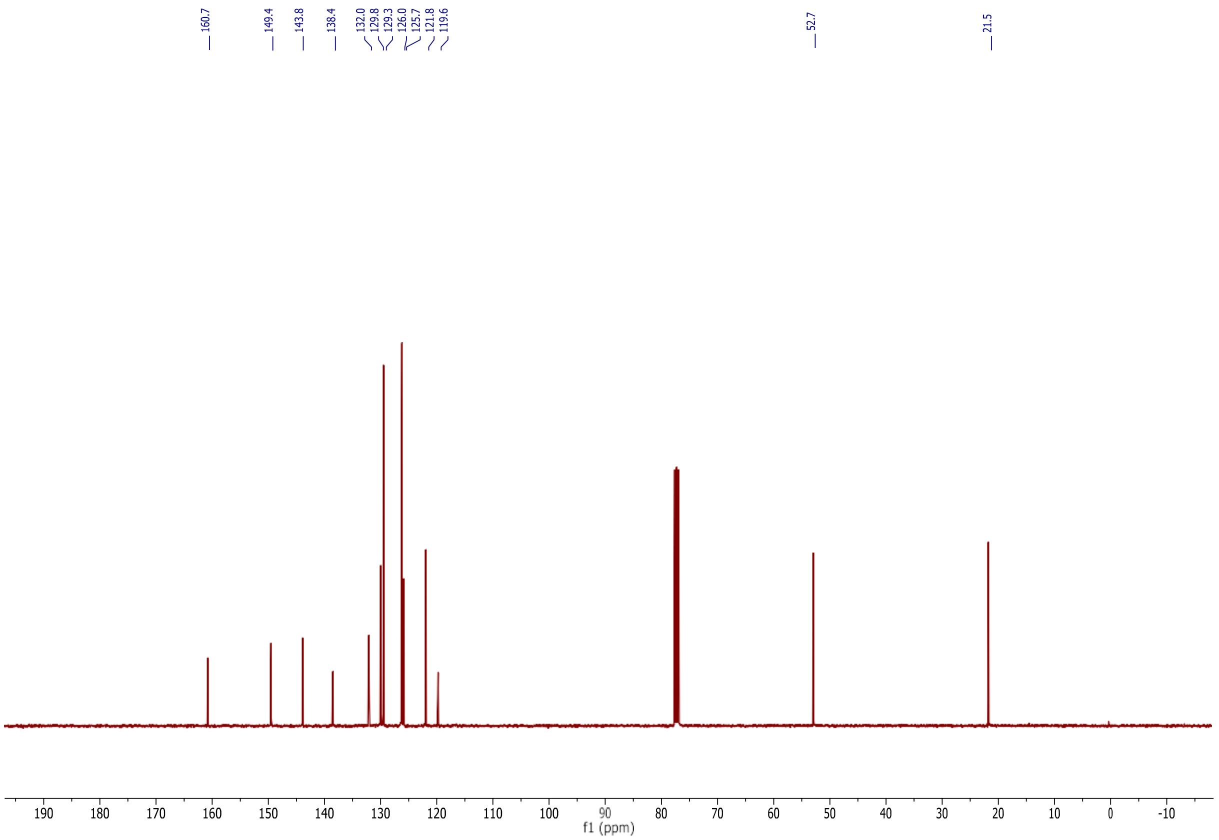
160.7
149.4
143.8
138.4
132.0
129.8
129.3
126.0
125.7
121.8
119.6
52.7
21.5
190 180 170 160 150 140 130 120 110 100 90 80 70 60 50 40 30 20 10 0 -10
f1 (ppm)

methyl 3-(5-(hydroxymethyl)-1H-1,2,3-triazol-1-yl)-5-phenylthiophene-2-carboxylate

4b

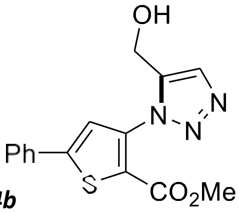

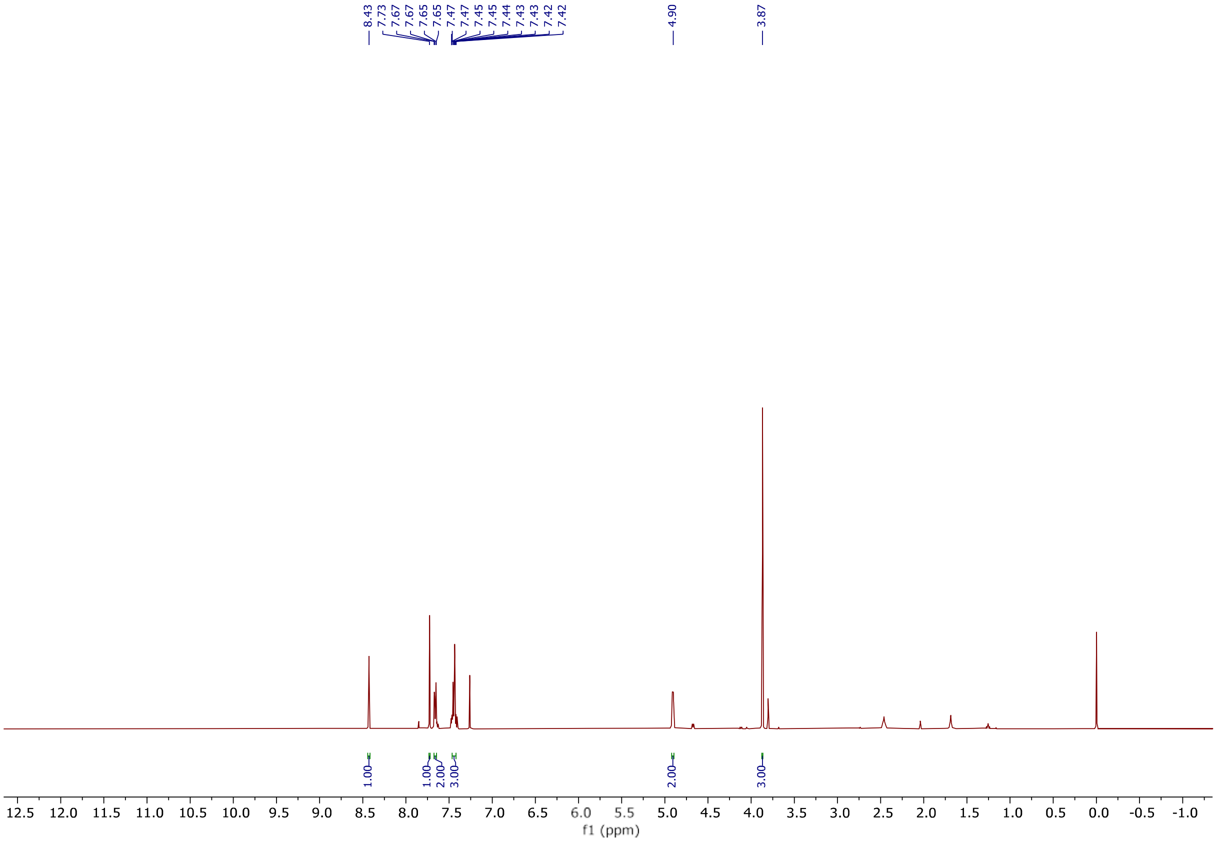

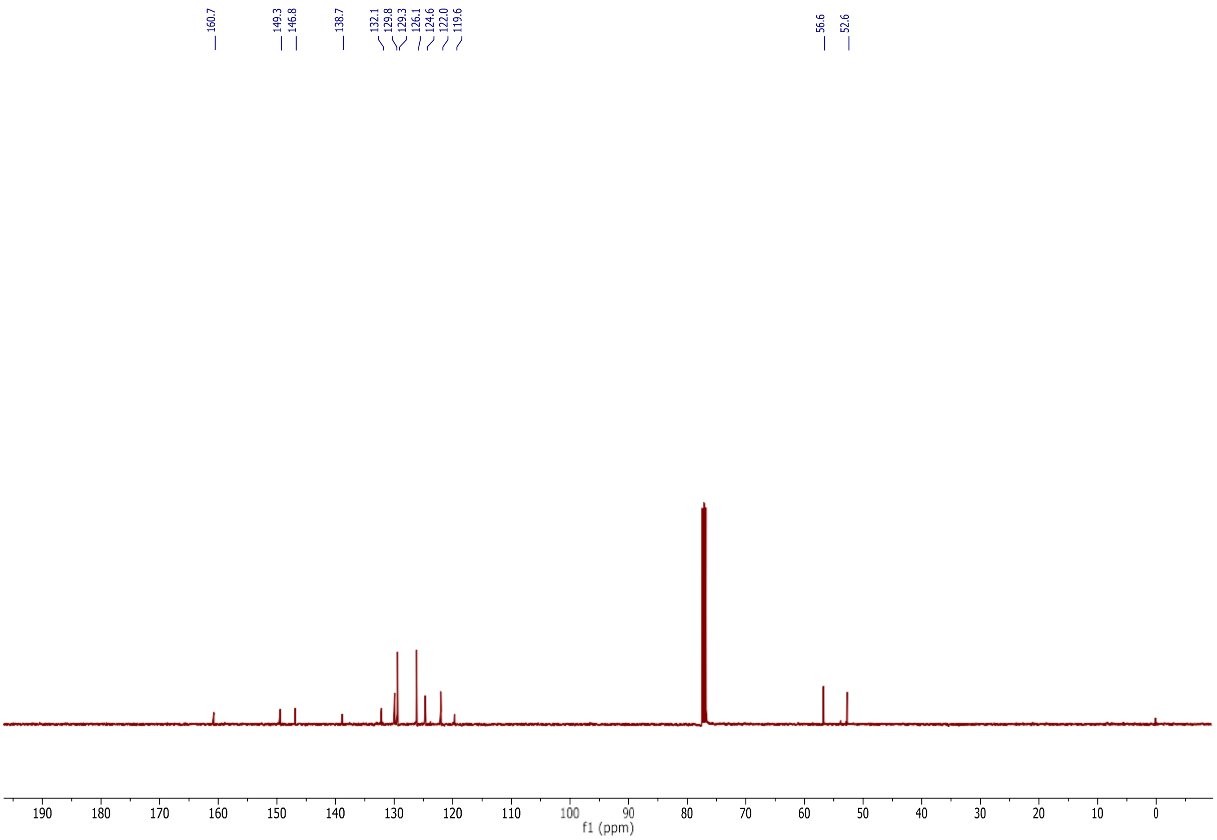
160.7
149.3
146.8
138.7
132.1
129.8
129.3
126.1
124.6
122.0
119.6
56.6
52.6
190
180
170
160
150
140
130
120
110
100
90
80
70
60
50
40
30
20
10
0
f1 (ppm)

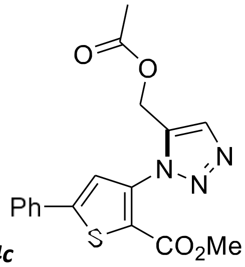

methyl 3-(5-(acetoxymethyl)-1H-1,2,3-triazol-1-yl)-5-phenylthiophene-2-carboxylate ***4c***

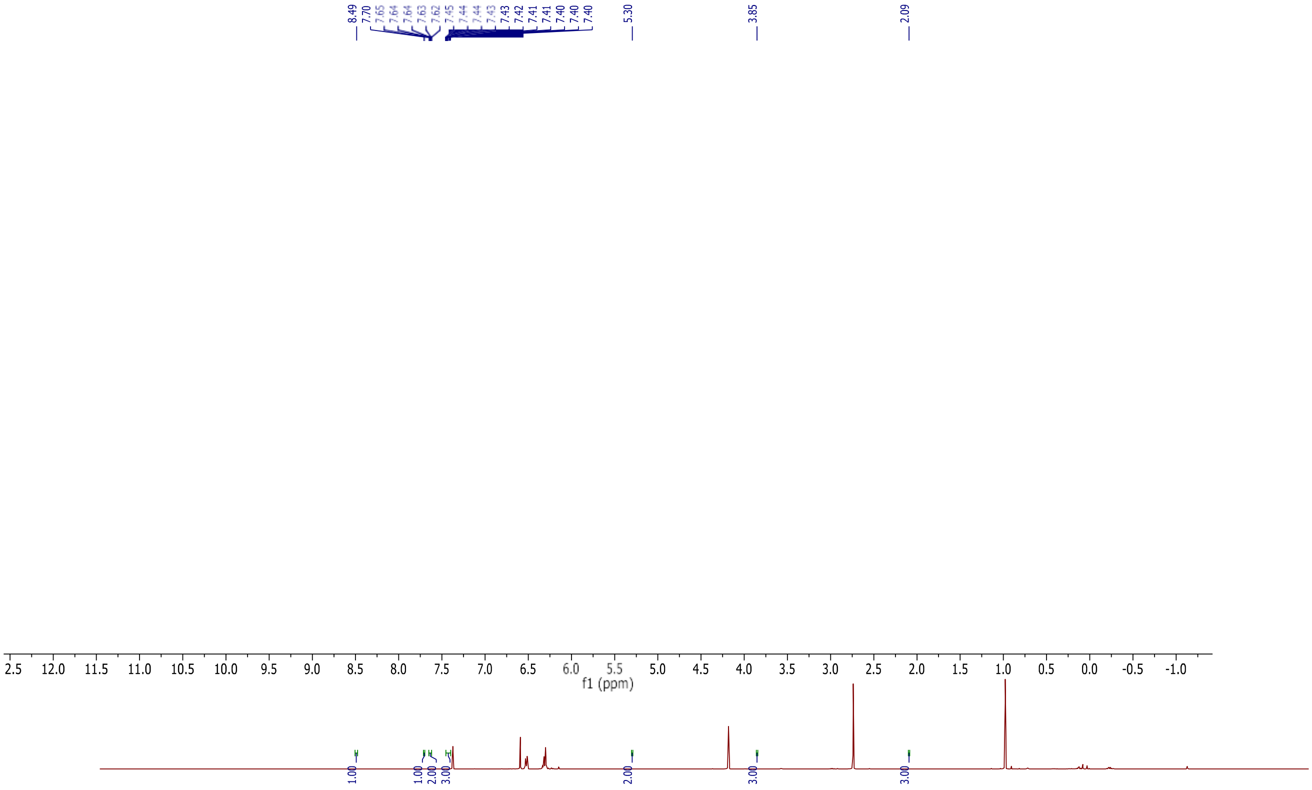

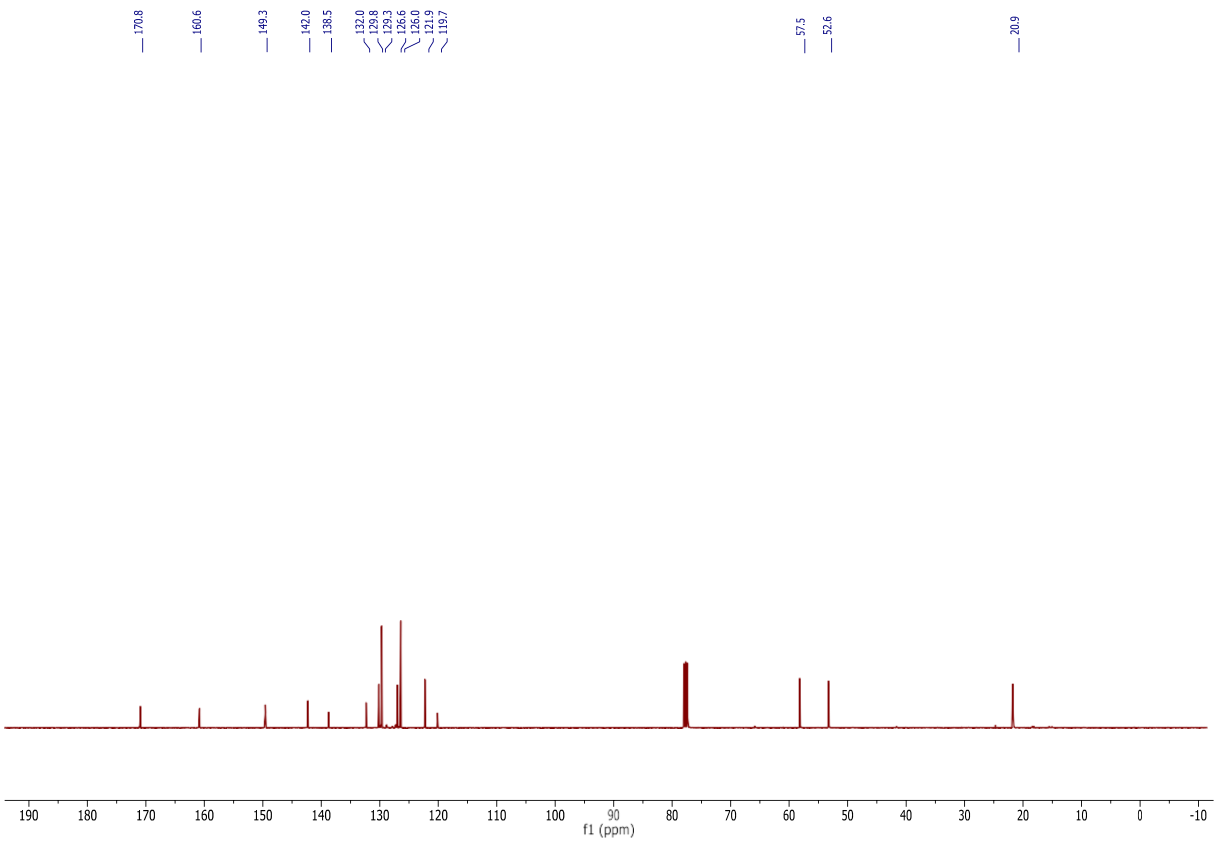
170.8
160.6
149.3
142.0
138.5
132.0
129.8
129.3
126.6
126.0
121.9
119.7
57.5
52.6
20.9
190
180
170
160
150
140
130
120
110
100
90
80
70
60
50
40
30
20
10
0
-10
f1 (ppm)

methyl 3-(5-((benzyl(methyl)amino)methyl)-1H-1,2,3-triazol-1-yl)-5-phenylthiophene-2-carboxylate ***4d***

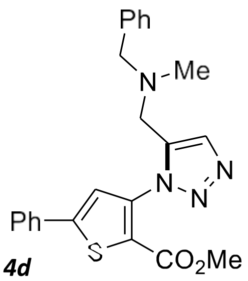

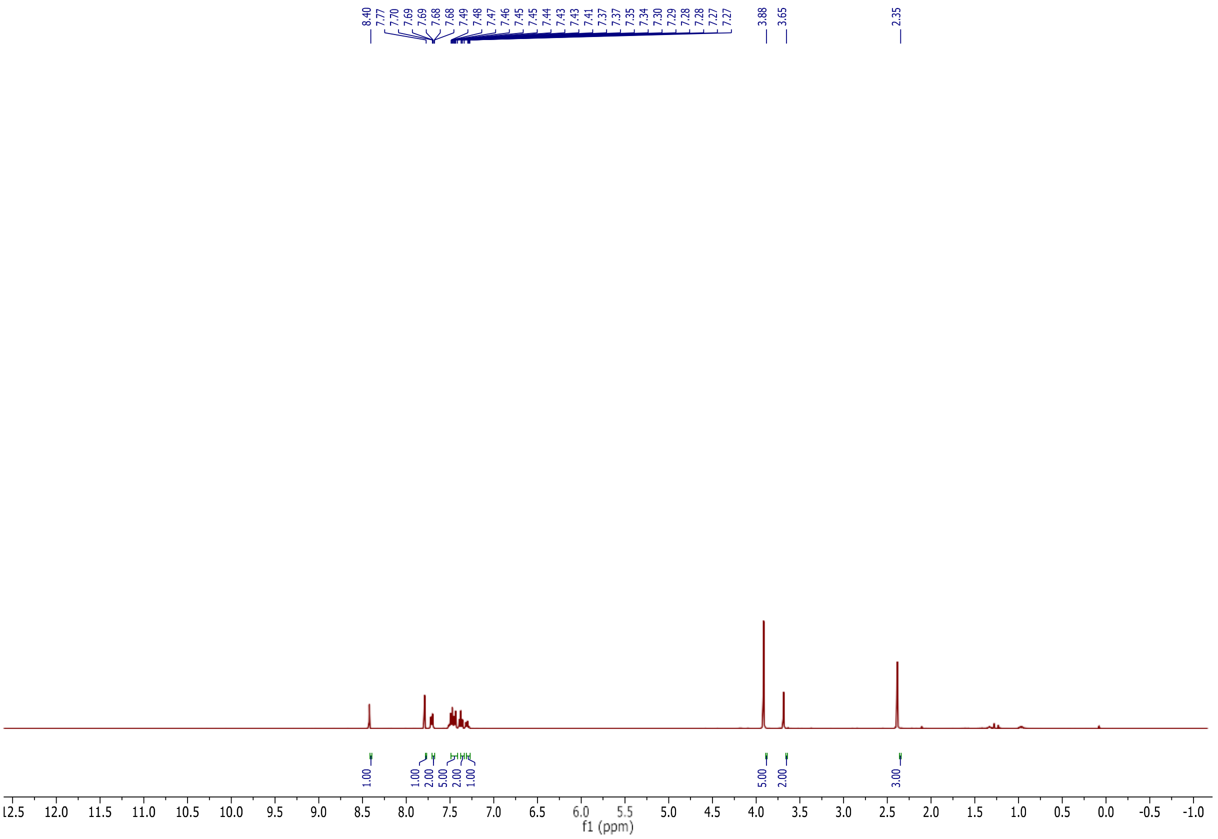

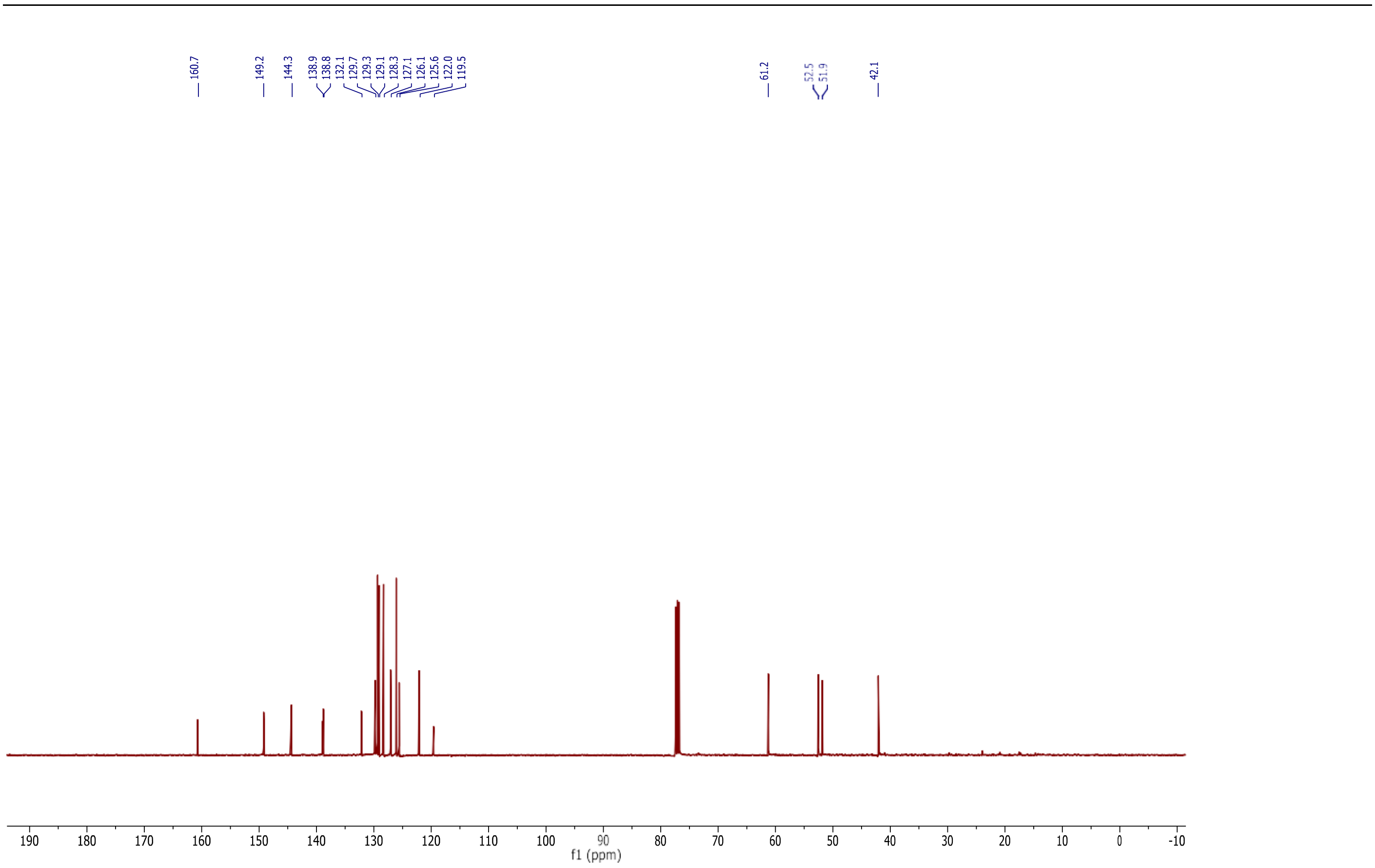
160.7
149.2
144.3
138.9
138.8
132.1
129.7
129.3
129.1
128.3
127.1
126.1
125.6
122.0
119.5
61.2
52.5
51.9
42.1
190 180 170 160 150 140 130 120 110 100 90 80 70 60 50 40 30 20 10 0 -10
f1 (ppm)

methy 3-(5-(bromomethyl)-1H-1,2,3-triazol-1-yl)thiophene-2-carboxylate

5a

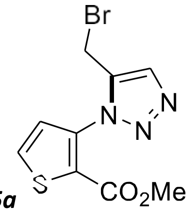

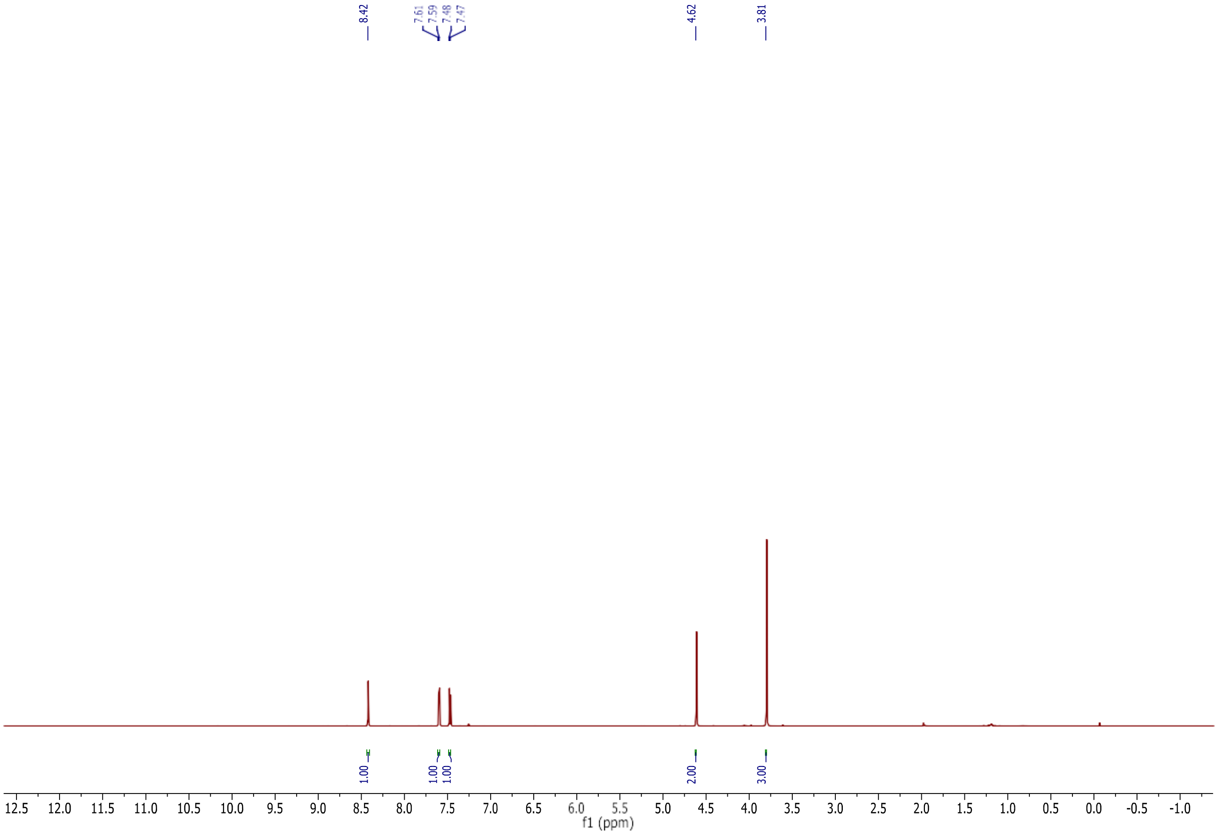

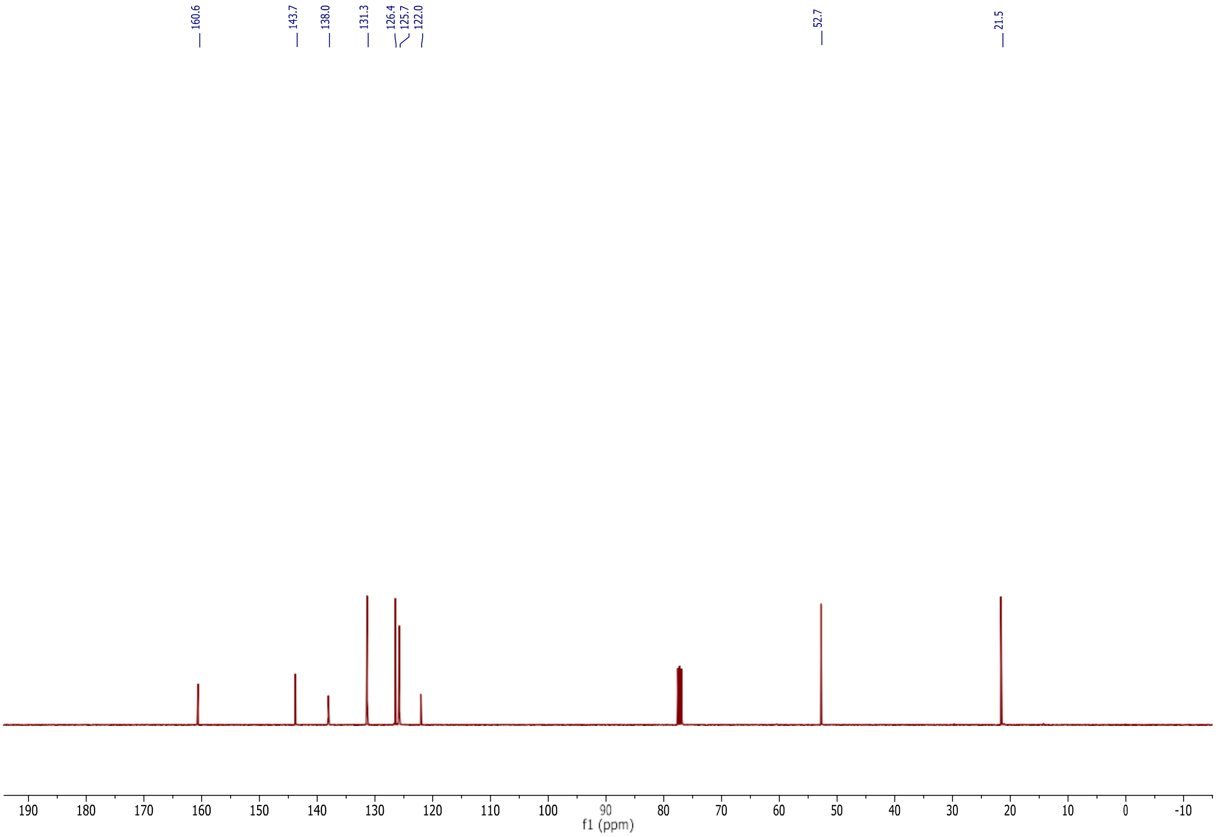

160.6
143.7
138.0
131.3
126.4
125.7
122.0
52.7
21.5
190 180 170 160 150 140 130 120 110 100 90 80 70 60 50 40 30 20 10 0 -10
f1 (ppm)

methyl 3-(5-(hydroxymethyl)-1H-1,2,3-triazol-1-yl)thiophene-2-carboxylate

5b

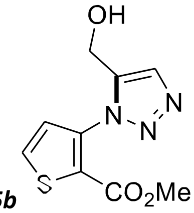

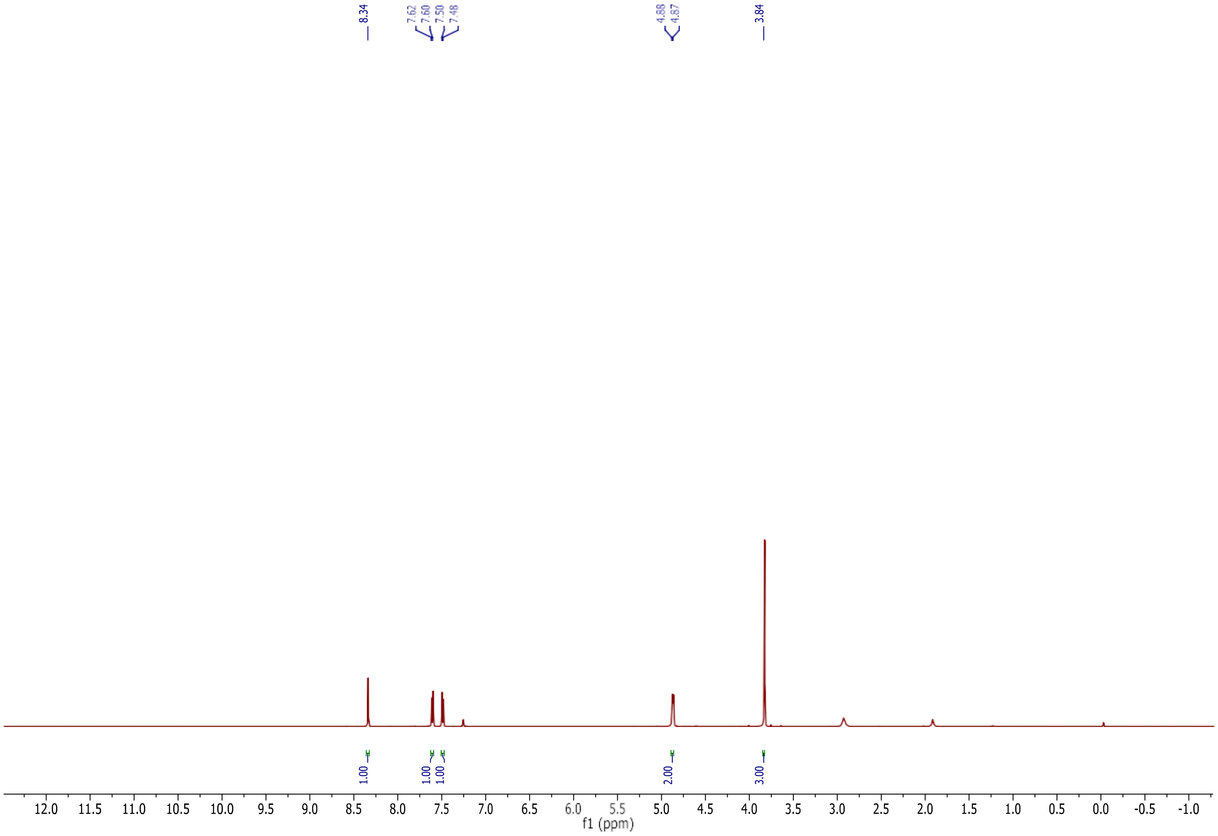

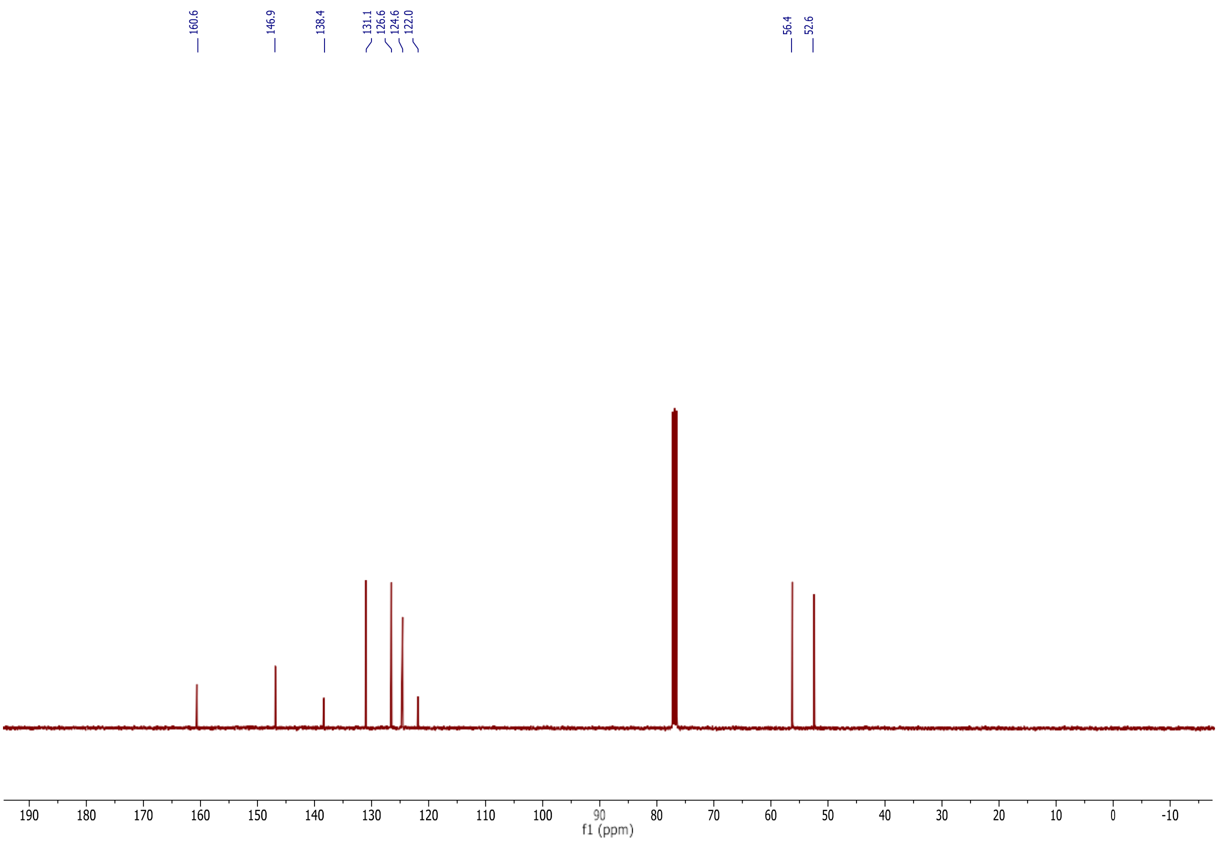
160.6
146.9
138.4
131.1
126.6
124.6
122.0
56.4
52.6
190 180 170 160 150 140 130 120 110 100 90 80 70 60 50 40 30 20 10 0 -10
f1 (ppm)

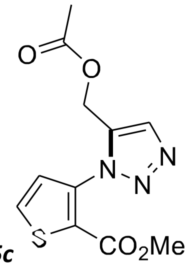

methyl 3-(5-(acetoxymethyl)-1H-1,2,3-triazol-1-yl)thiophene-2-carboxylate ***5c***

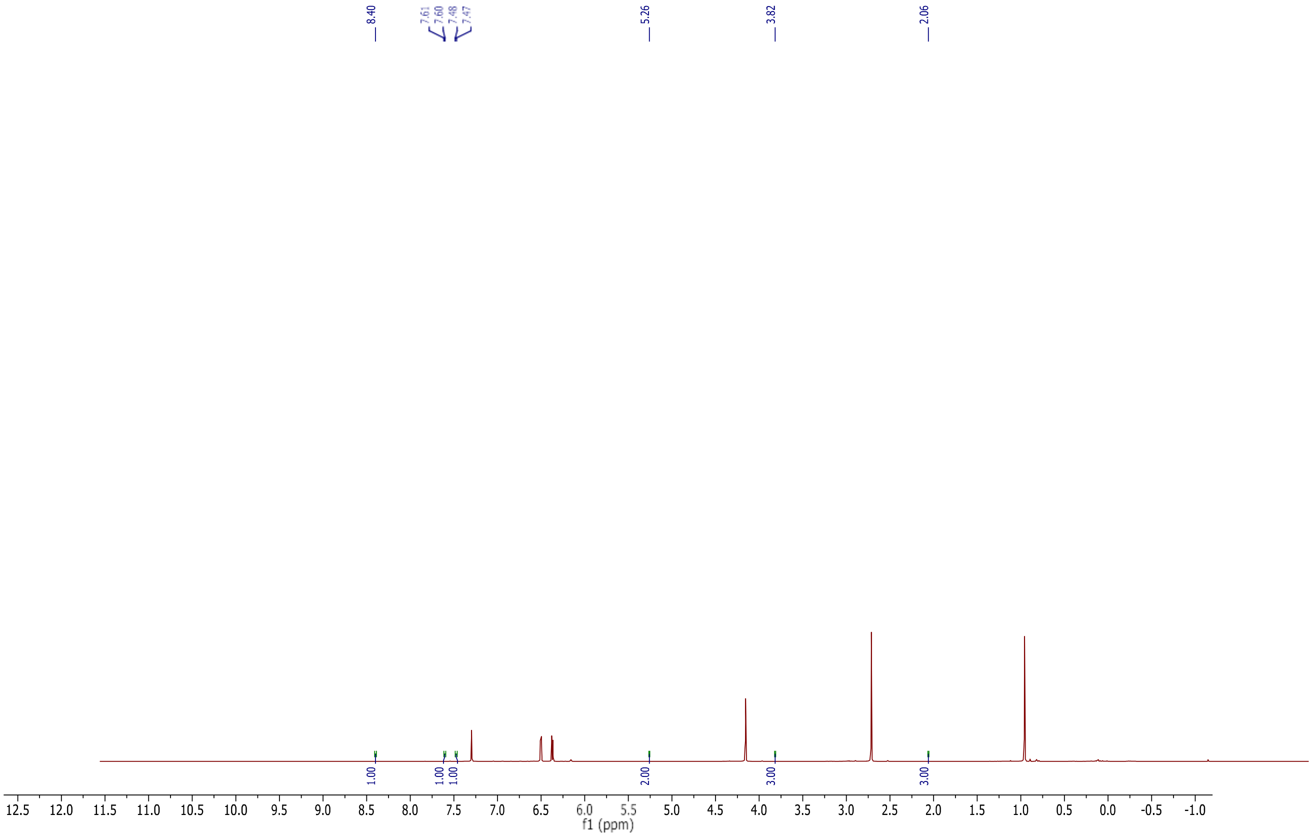

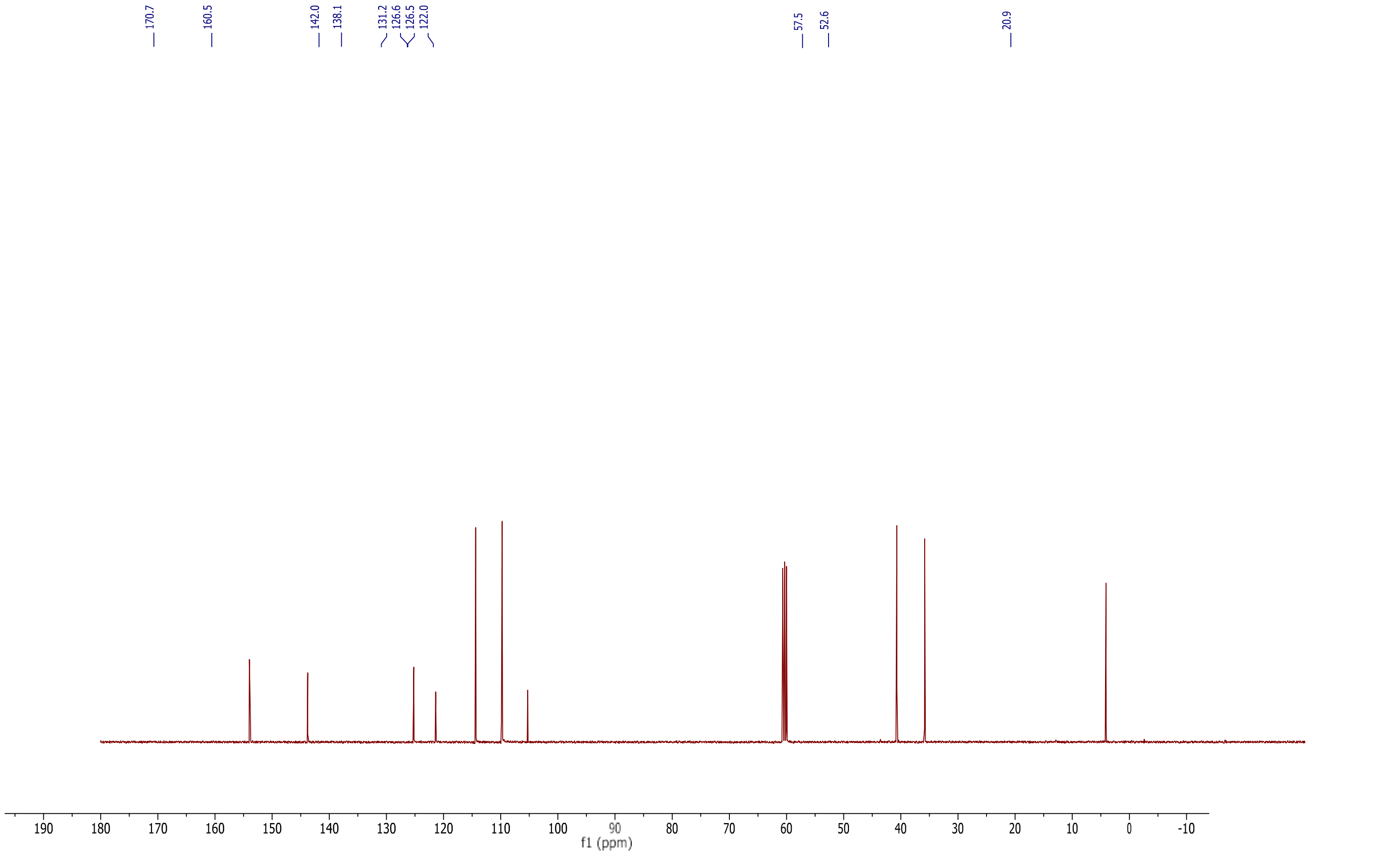
170.7
160.5
142.0
138.1
131.2
126.6
126.5
122.0
57.5
52.6
20.9
190 180 170 160 150 140 130 120 110 100 90 80 70 60 50 40 30 20 10 0 -10
f1 (ppm)

methyl 3-(5-((benzyl(methyl)amino)methyl)-1H-1,2,3-triazol-1-yl)thiophene-2-carboxylate **5d**

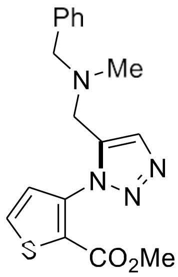

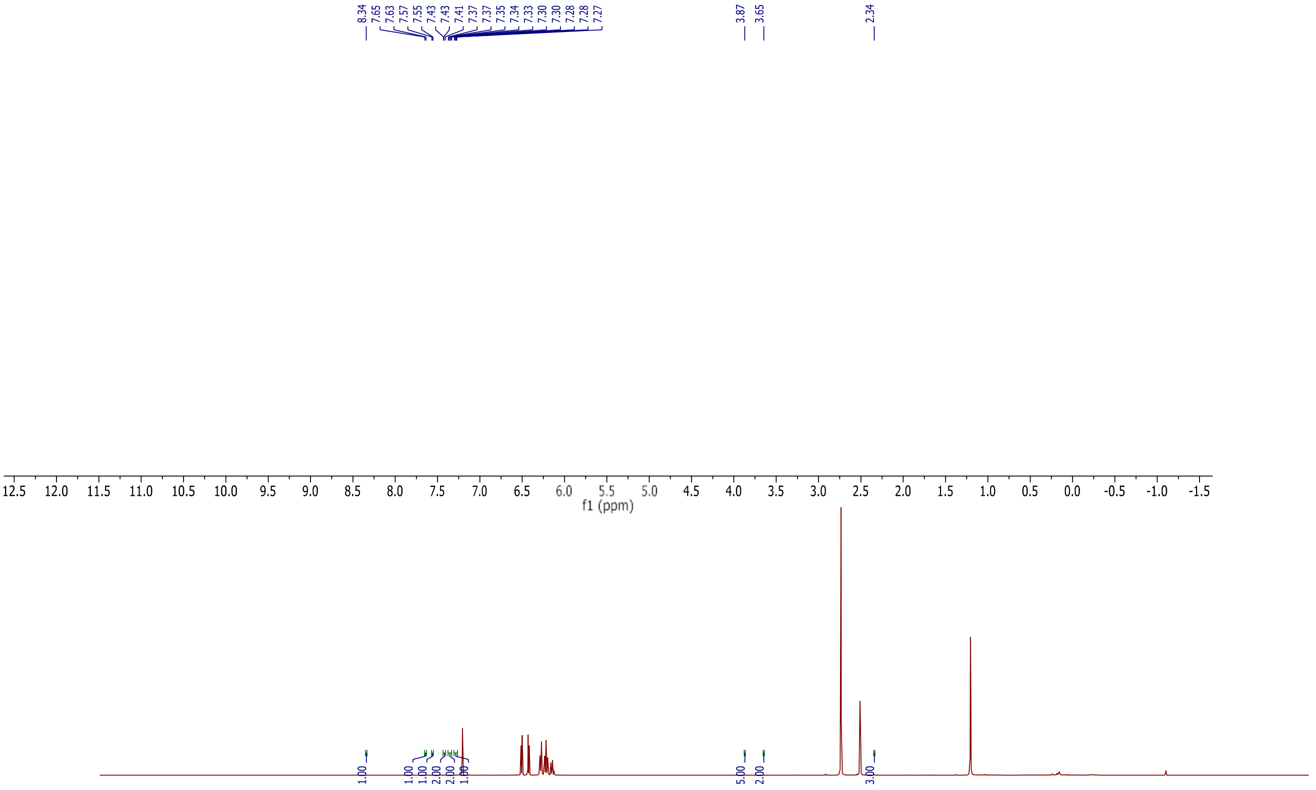

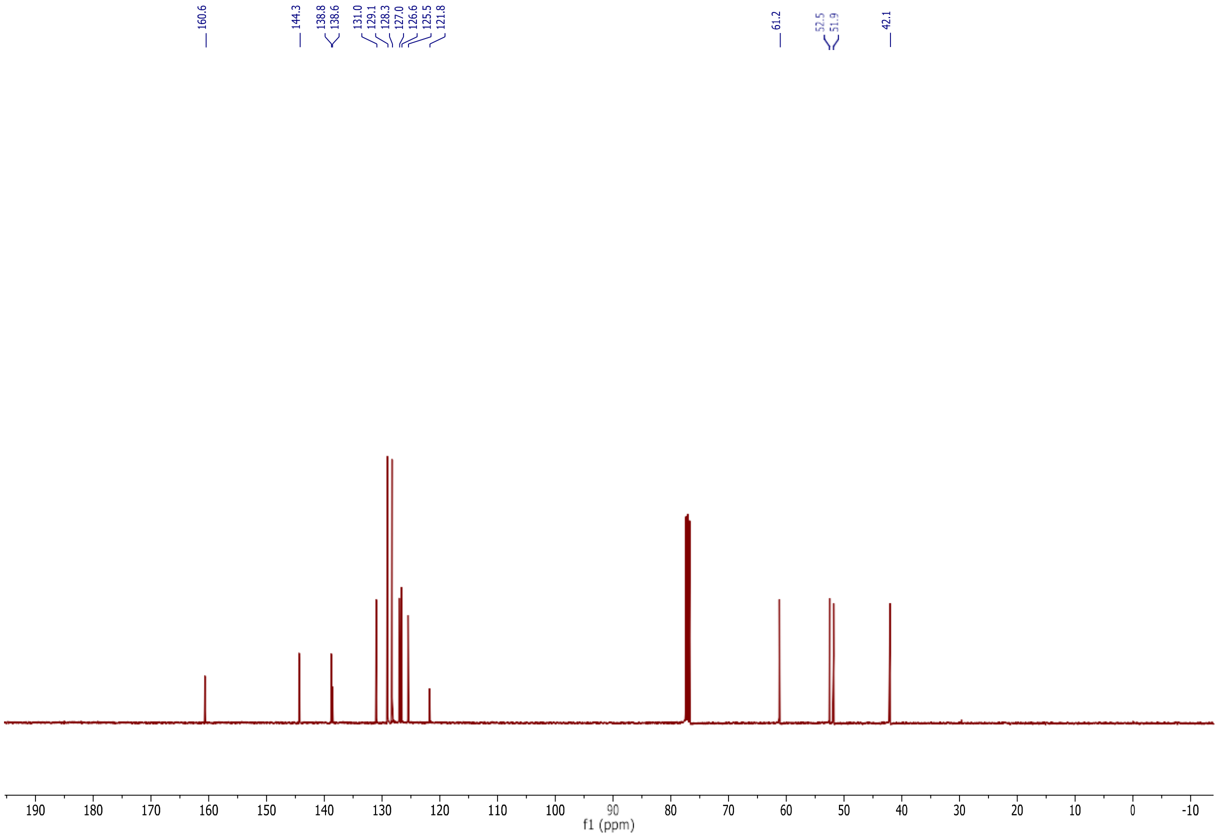
160.6
144.3
138.8
138.6
131.0
129.1
128.3
127.0
126.6
125.5
121.8
61.2
52.5
51.9
42.1
190
180
170
160
150
140
130
120
110
100
90
80
70
60
50
40
30
20
10
0
-10
f1 (ppm)

methyl 4-(5-(bromomethyl)-1H-1,2,3-triazol-1-yl)thiazole-5-carboxylate

6a

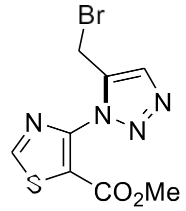

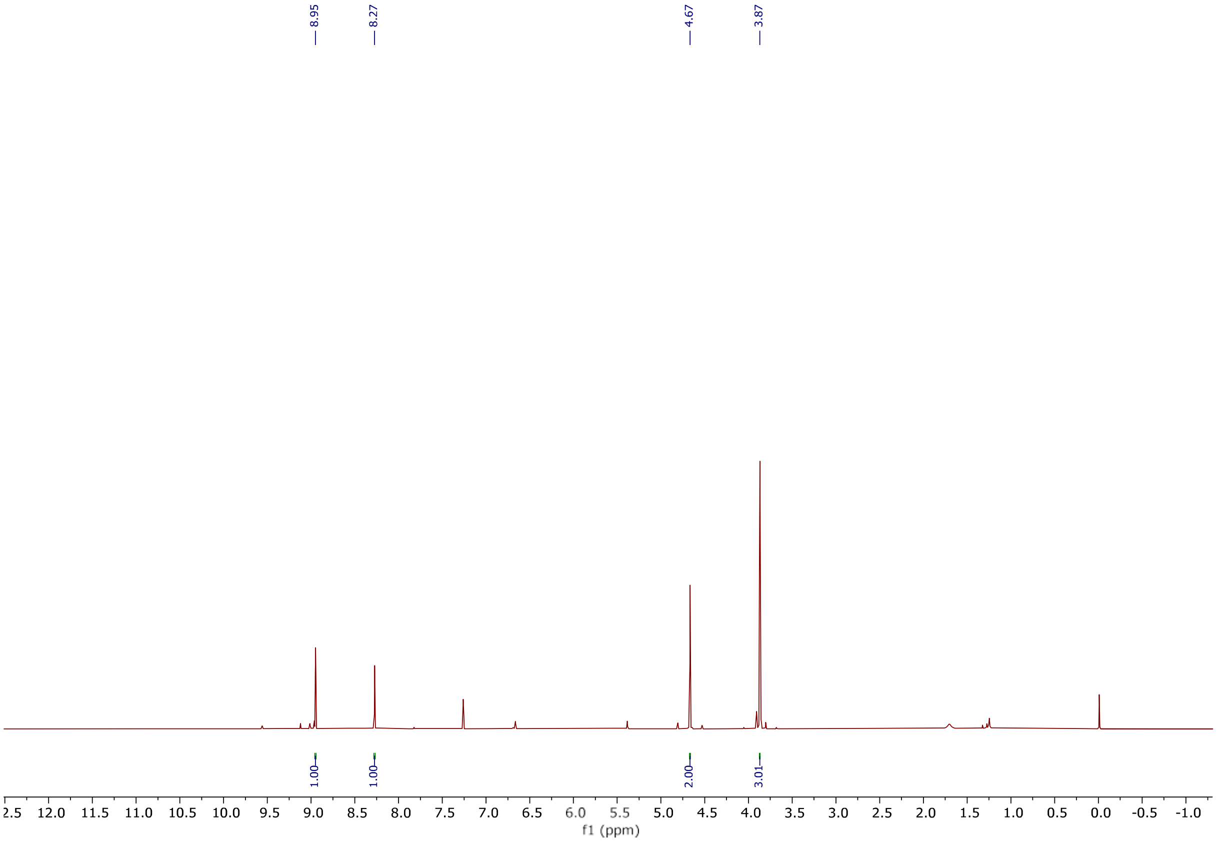

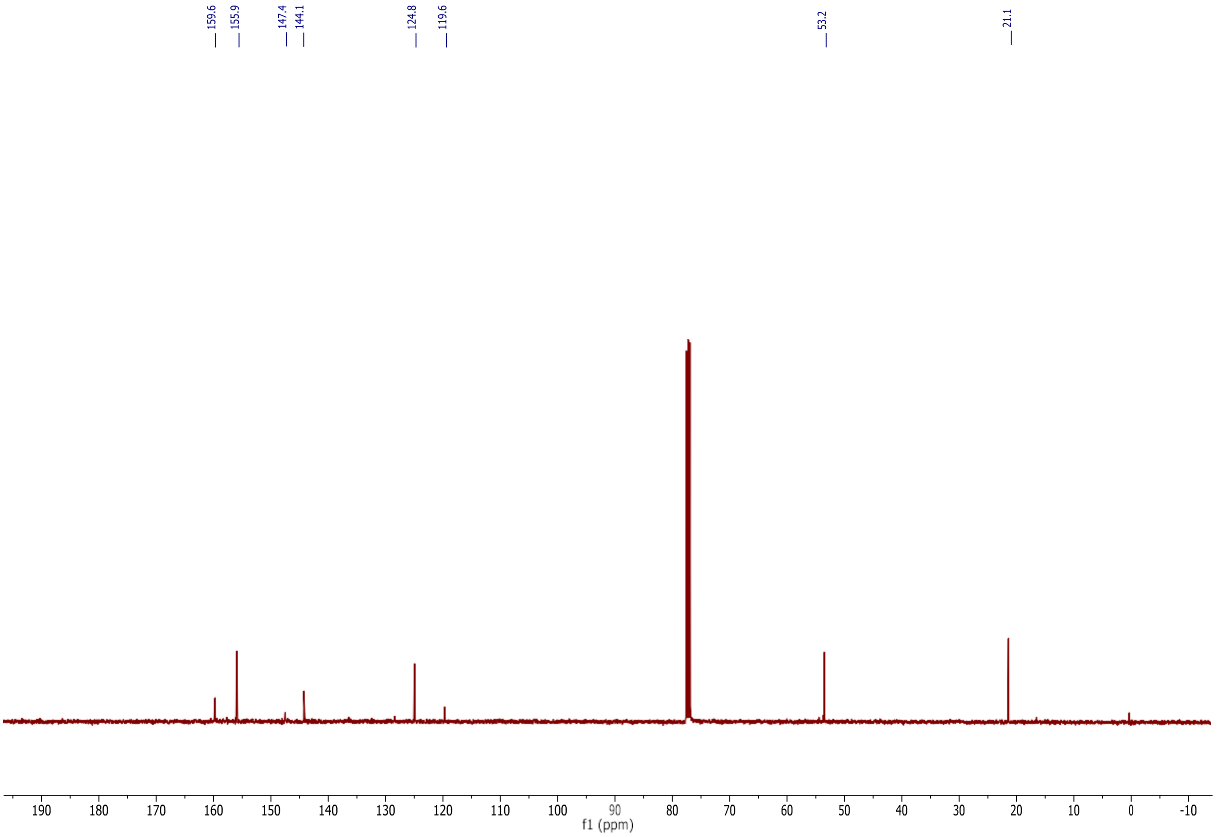
159.6
155.9
147.4
144.1
124.8
119.6
53.2
21.1
190 180 170 160 150 140 130 120 110 100 90 80 70 60 50 40 30 20 10 0 -10
f1 (ppm)

methyl 4-(5-(hydroxymethyl)-1H-1,2,3-triazol-1-yl)thiazole-5-carboxylate 6b

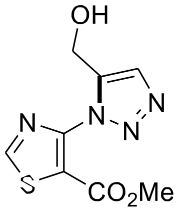

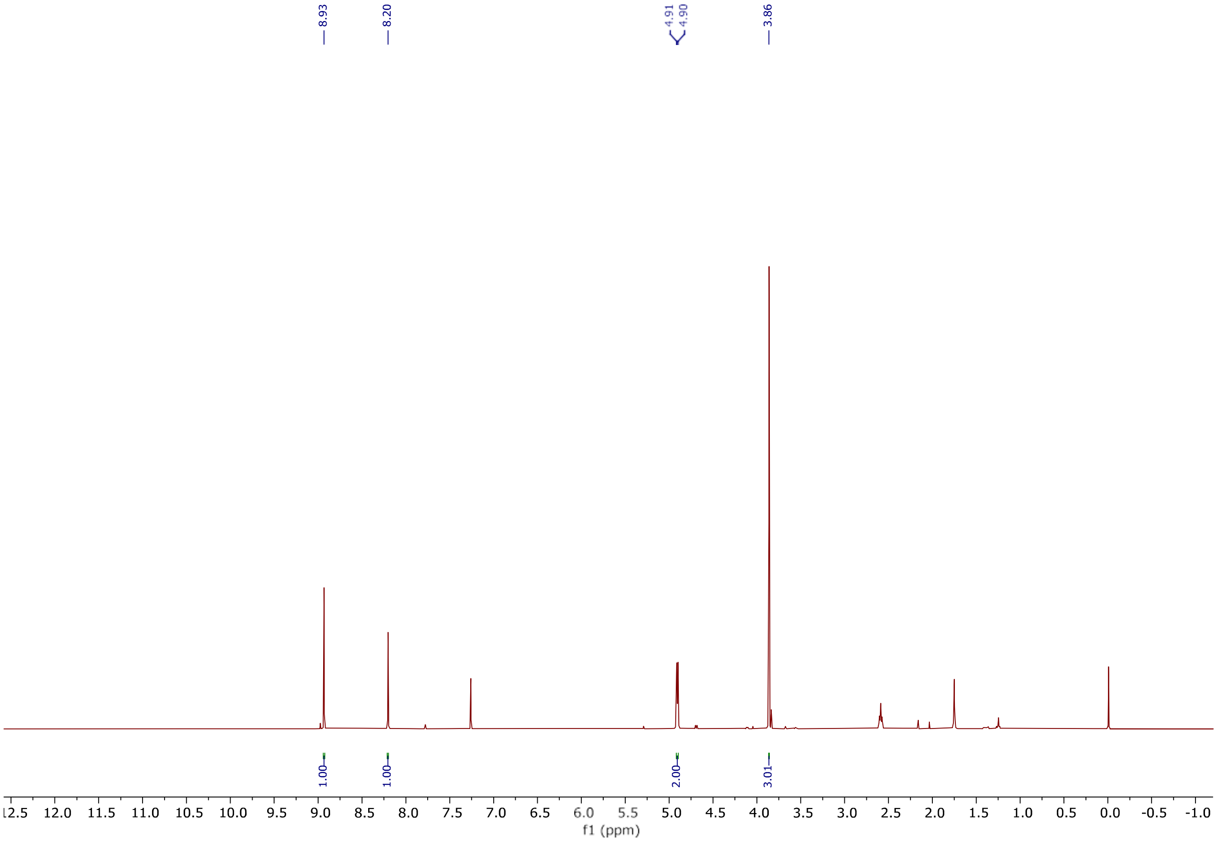

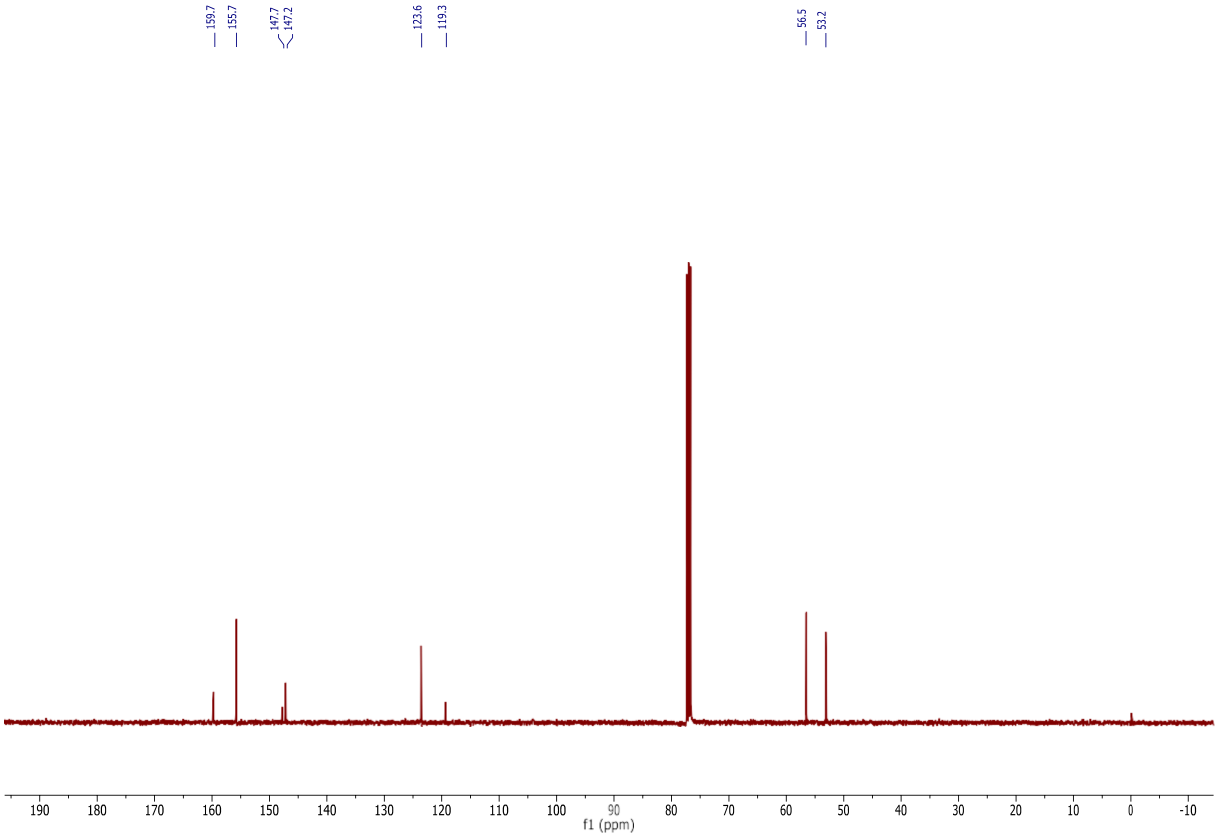
159.7
155.7
147.7
147.2
123.6
119.3
56.5
53.2
190 180 170 160 150 140 130 120 110 100 90 80 70 60 50 40 30 20 10 0 -10
f1 (ppm)

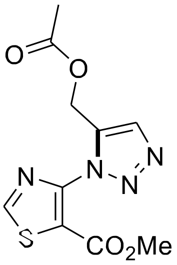

8.93
8.25
5.29
3.84
2.08

methyl 4-(5-(acetoxymethyl)-1H-1,2,3-triazol-1-yl)thiazole-5-carboxylate 6c

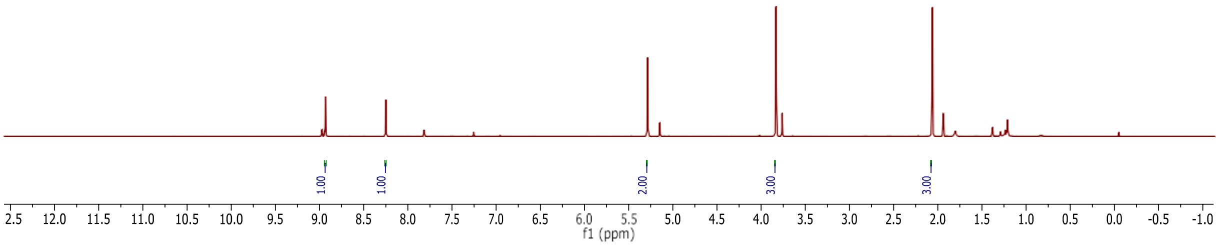

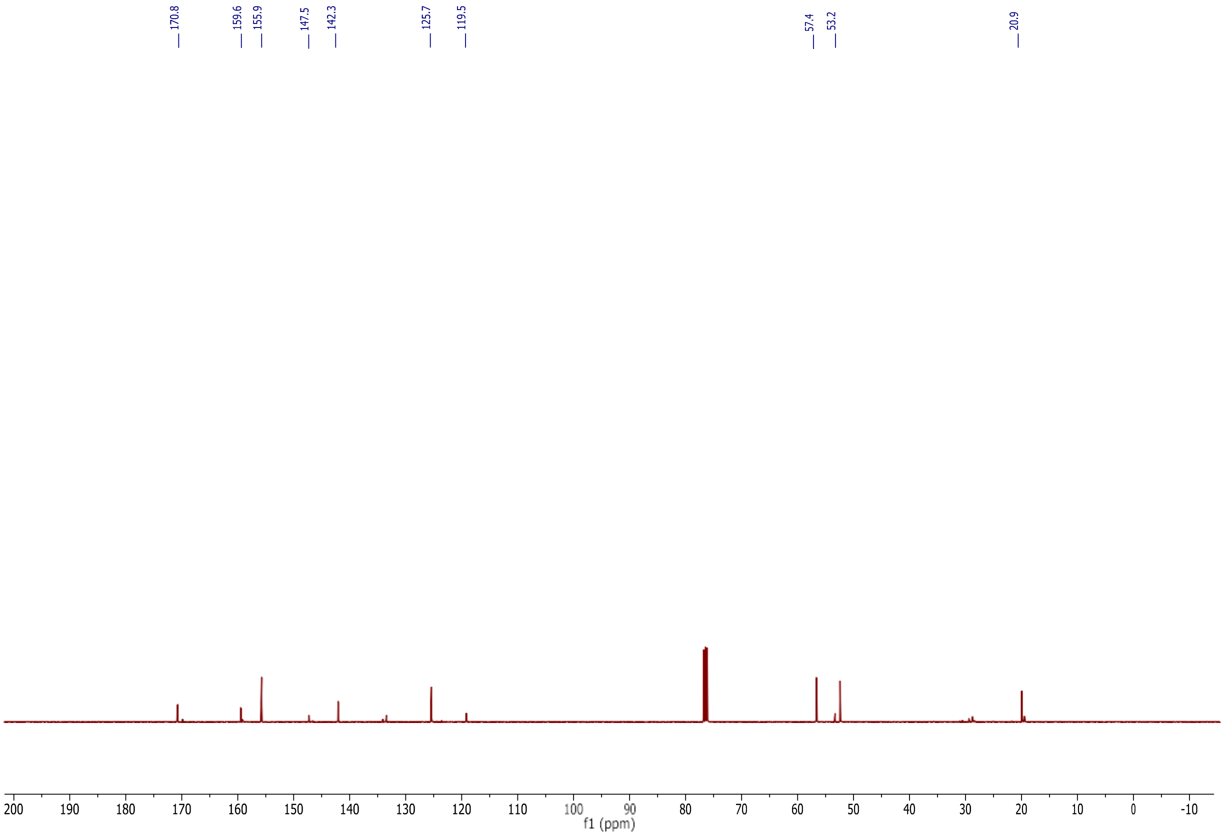
170.8
159.6
155.9
147.5
142.3
125.7
119.5
57.4
53.2
20.9
200 190 180 170 160 150 140 130 120 110 100 90 80 70 60 50 40 30 20 10 0 -10
f1 (ppm)

methyl 4-(5-((benzyl(methyl)amino)methyl)-1H-1,2,3-triazol-1-yl)thiazole-5-carboxylate **6d**

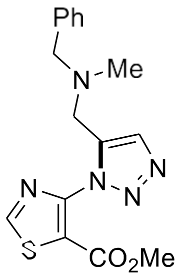

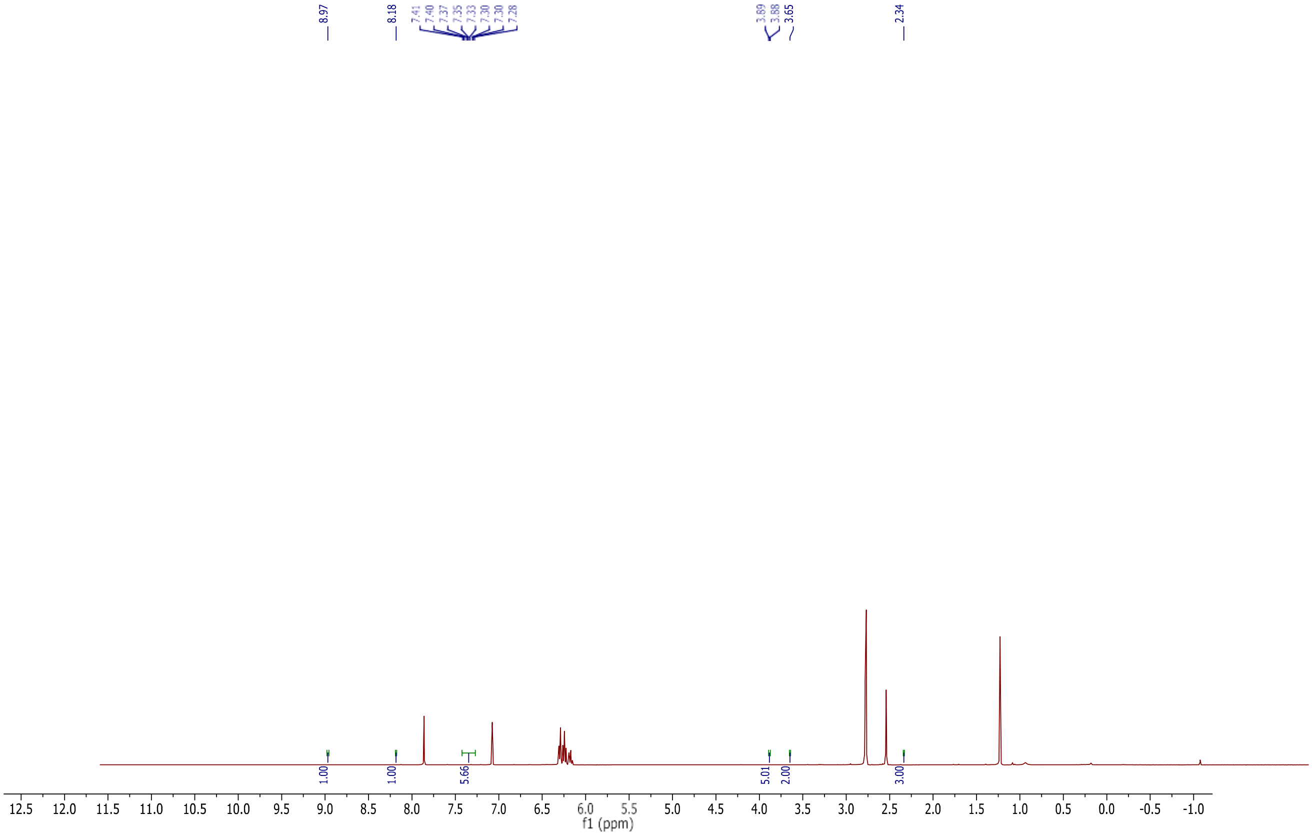

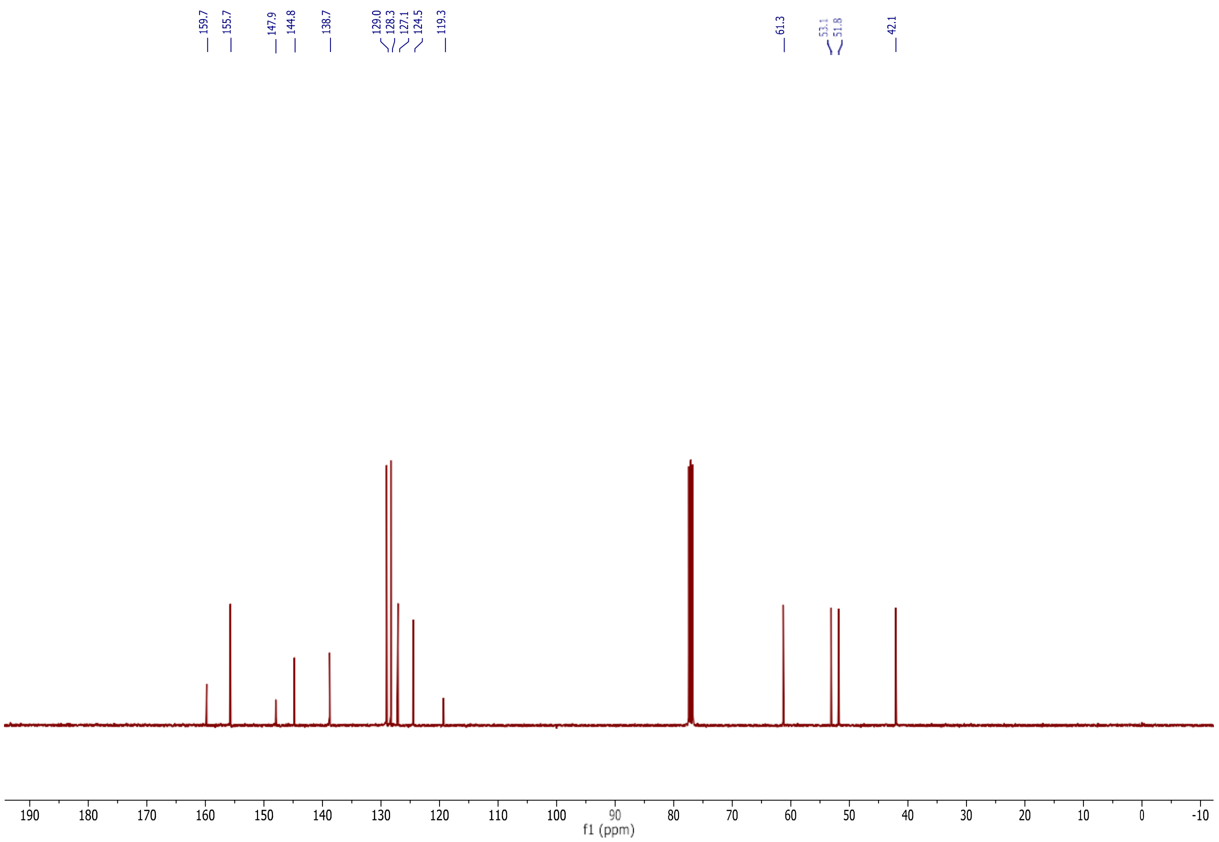

159.7
155.7
147.9
144.8
138.7
129.0
128.3
127.1
124.5
119.3
61.3
53.1
51.8
42.1
190
180
170
160
150
140
130
120
110
100
90
80
70
60
50
40
30
20
10
0
-10
f1 (ppm)

10H-pyrido[1,2-a]thieno[3,2-d]pyrimidin-10-one **8a**

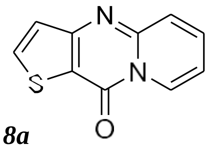

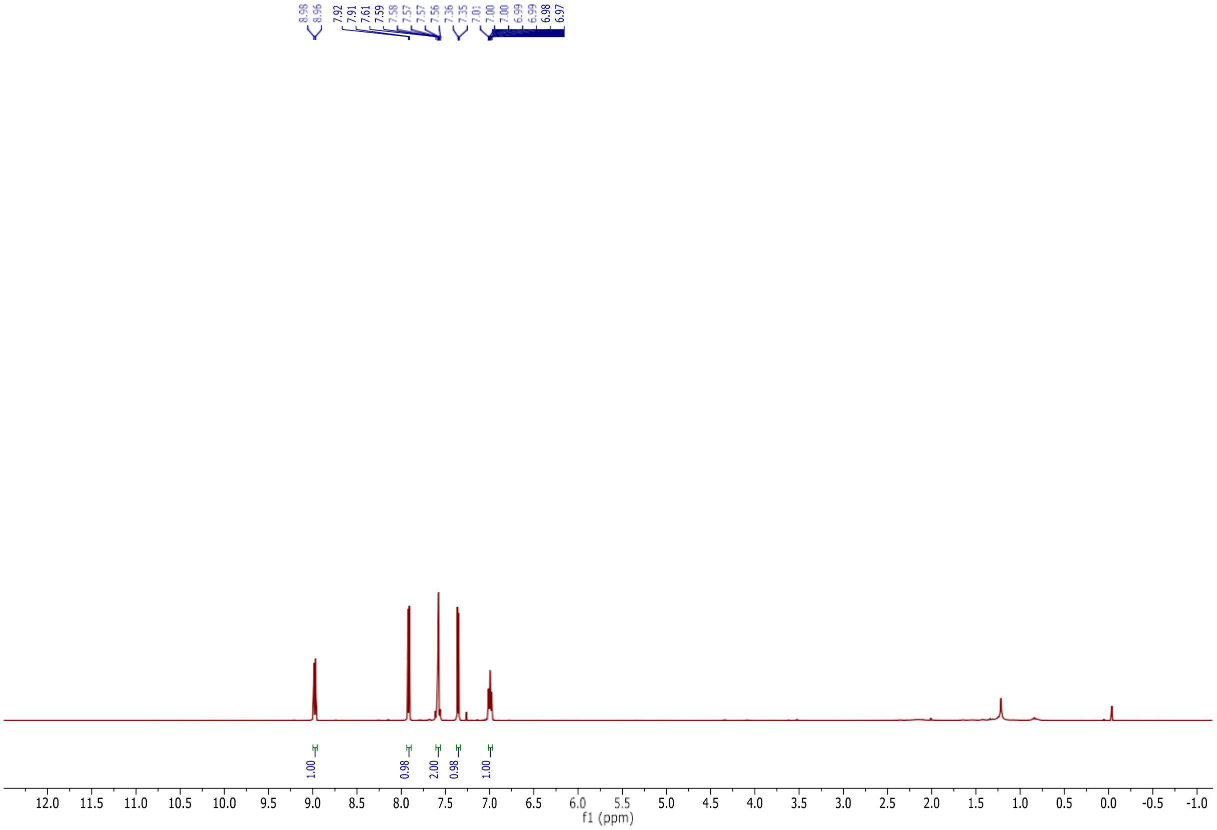

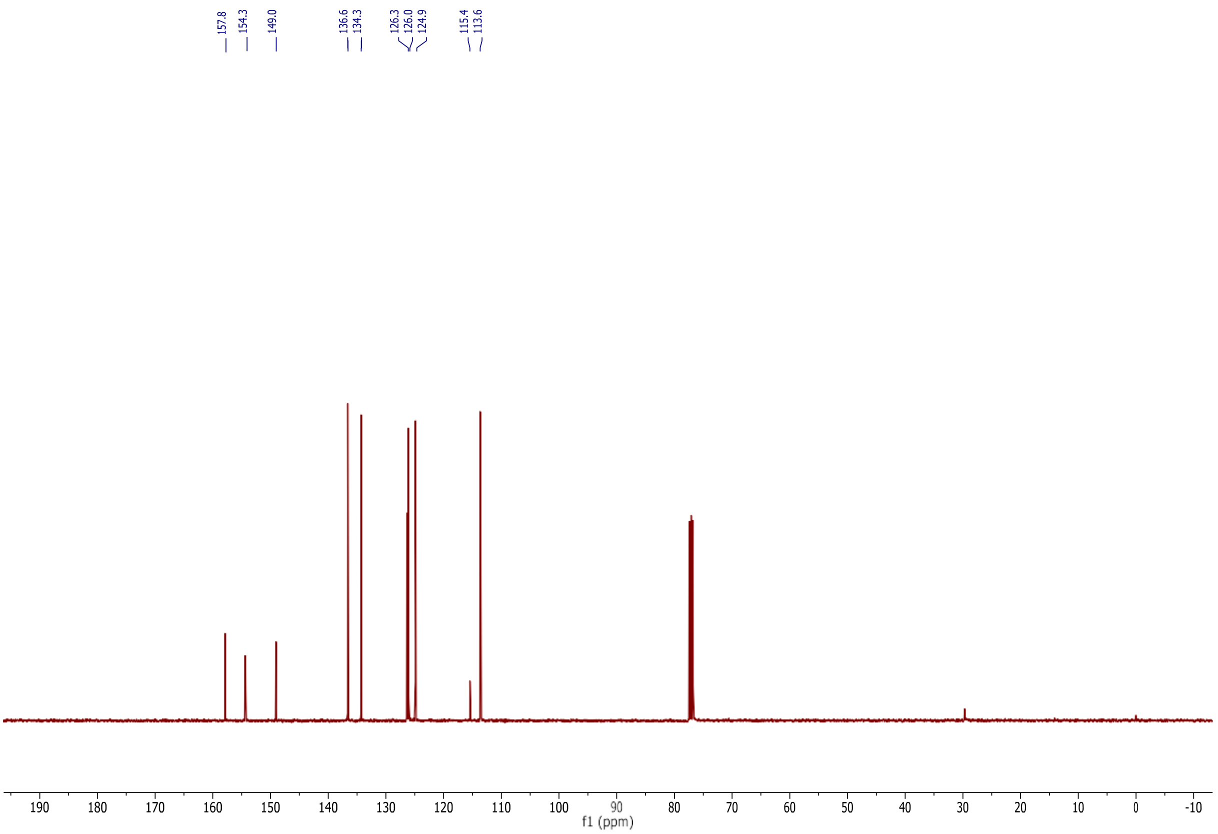
157.8
154.3
149.0
136.6
134.3
126.3
126.0
124.9
115.4
113.6
190 180 170 160 150 140 130 120 110 100 90 80 70 60 50 40 30 20 10 0 -10
f1 (ppm)

7-chloro-10H-pyrido[1,2-a]thieno[3,2-d]pyrimidin-10-one

8b

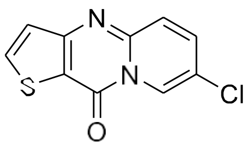

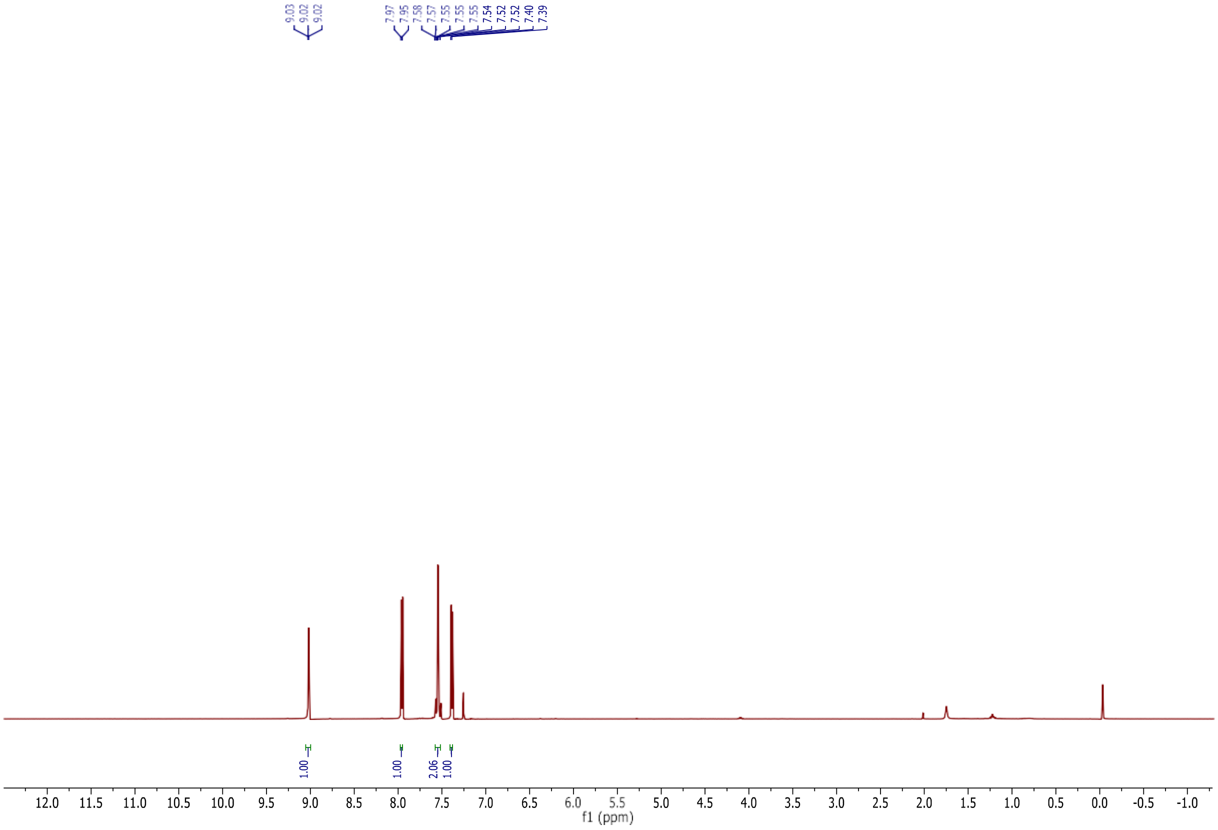

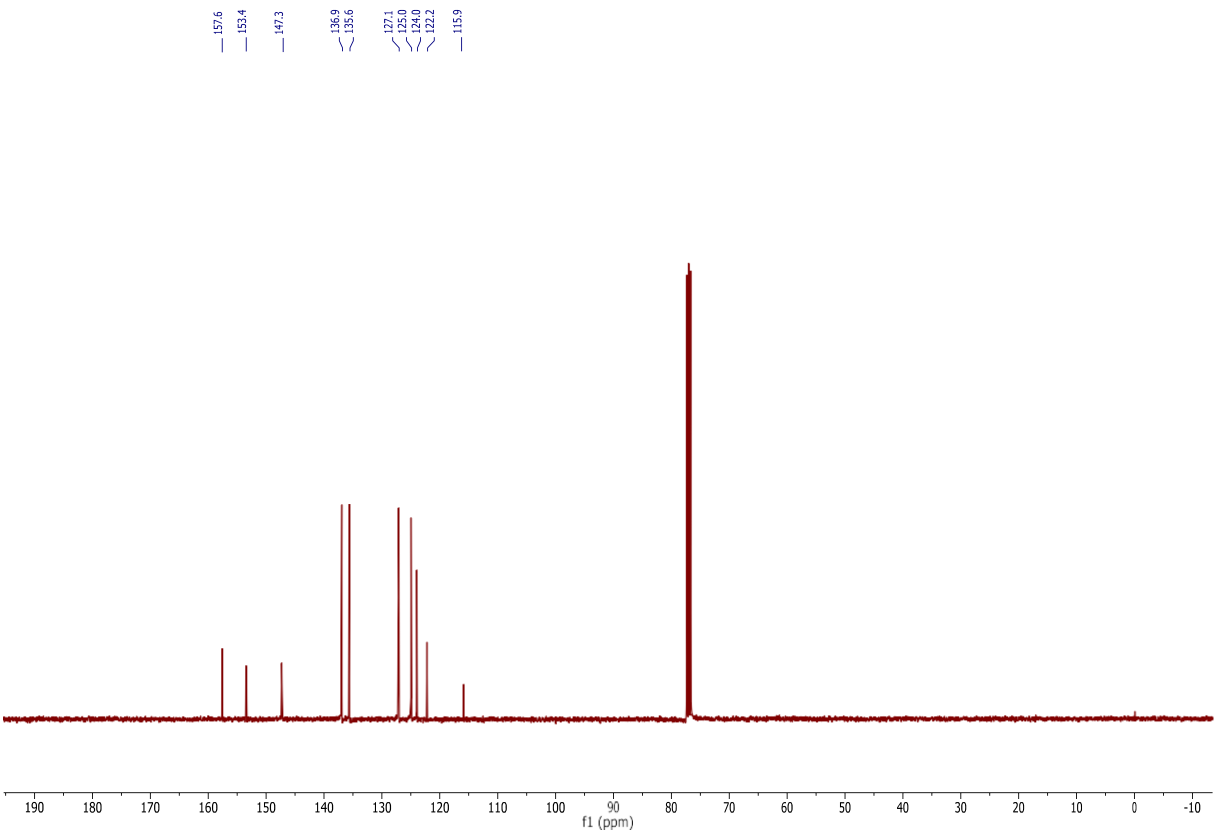

157.6
153.4
147.3
136.9
135.6
127.1
125.0
124.0
122.2
115.9
190 180 170 160 150 140 130 120 110 100 90 80 70 60 50 40 30 20 10 0 -10
f1 (ppm)

7-methyl-10H-pyrido[1,2-a]thieno[3,2-d]pyrimidin-10-one **8c**

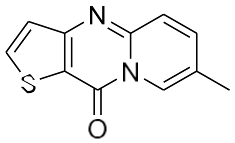

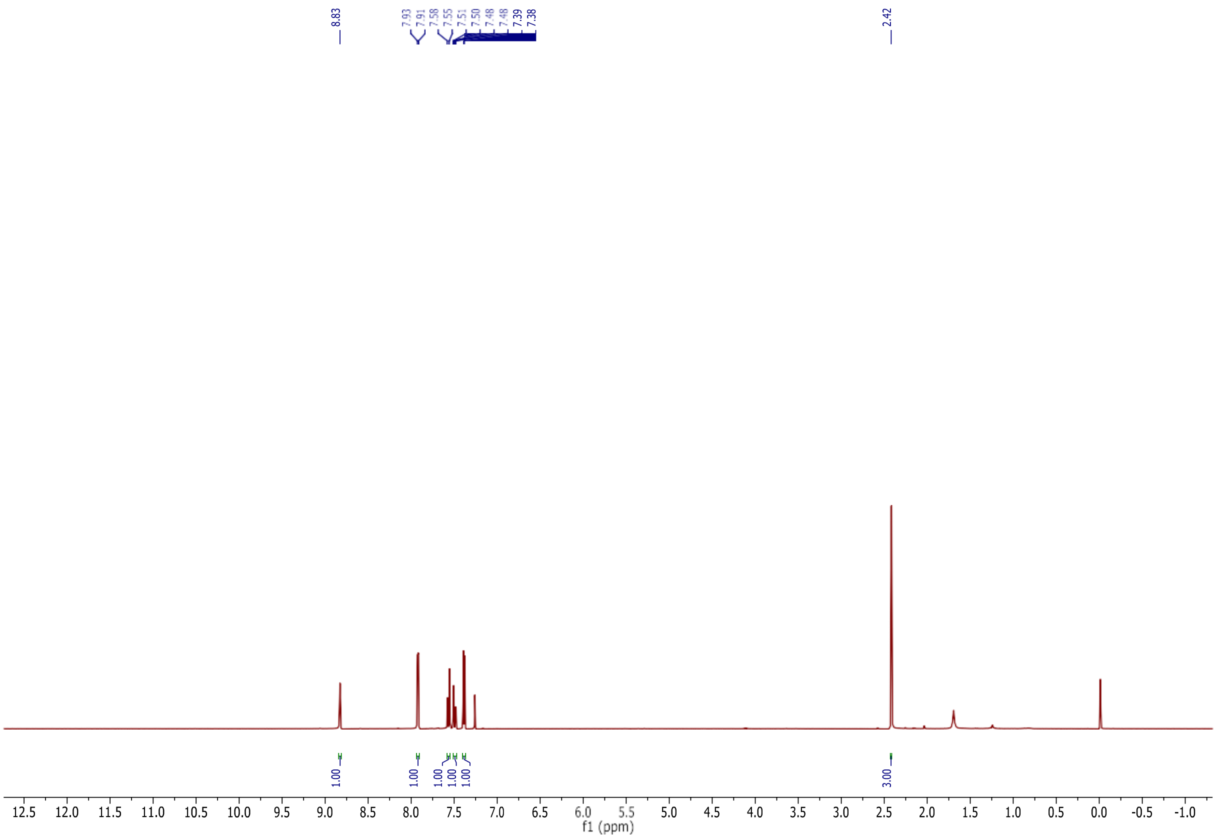

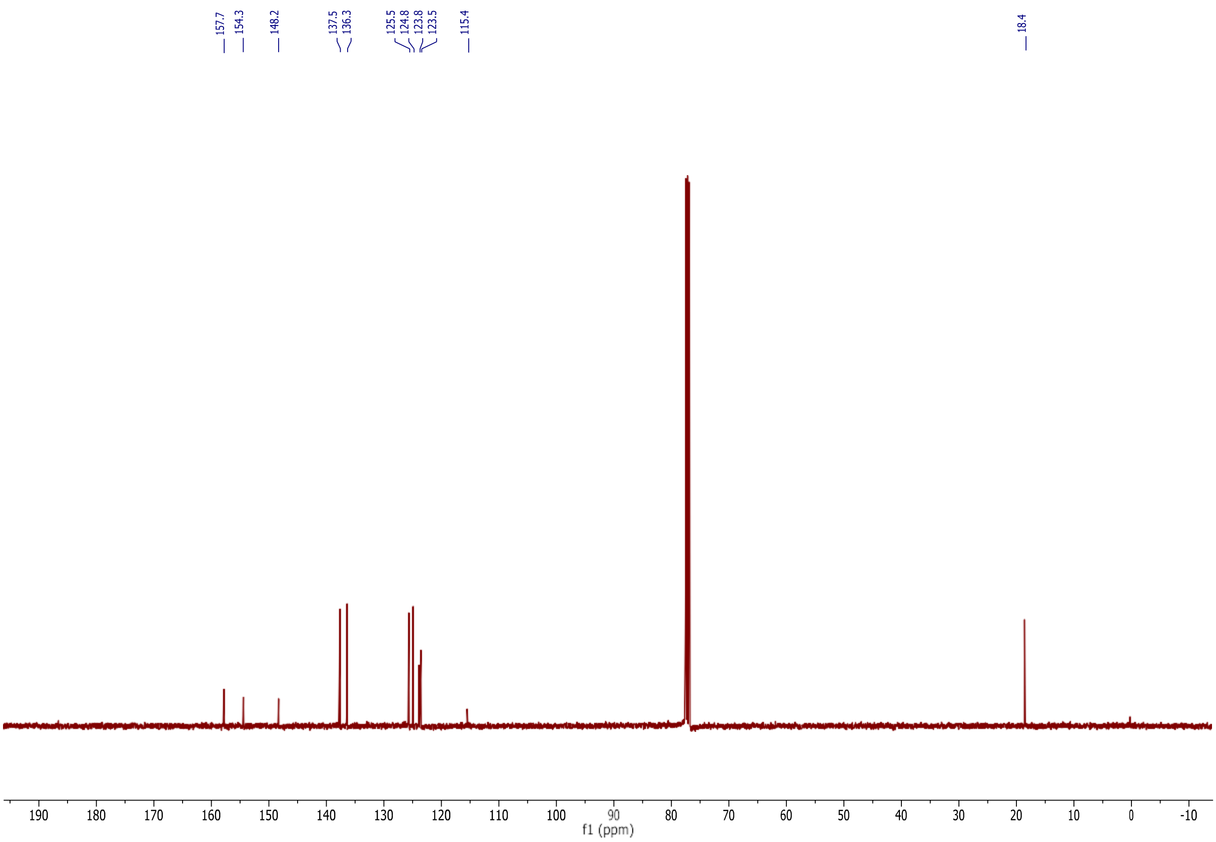

157.7
154.3
148.2
137.5
136.3
125.5
124.8
123.8
123.5
115.4
18.4
190
180
170
160
150
140
130
120
110
100
90
80
70
60
50
40
30
20
10
0
-10
f1 (ppm)

7-methoxy-10H-pyrido[1,2-a]thieno[3,2-d]pyrimidin-10-one ***8d***

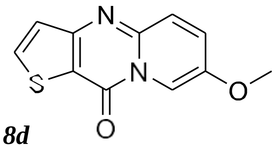

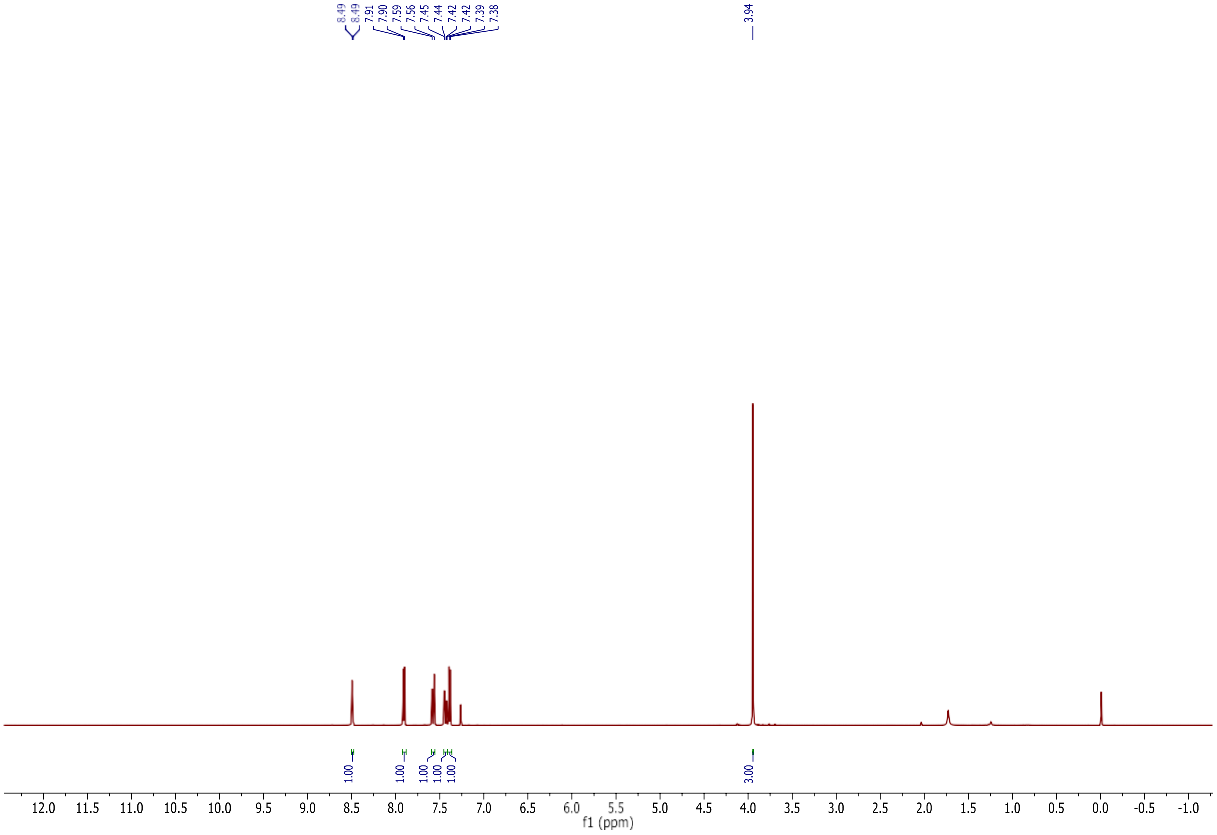

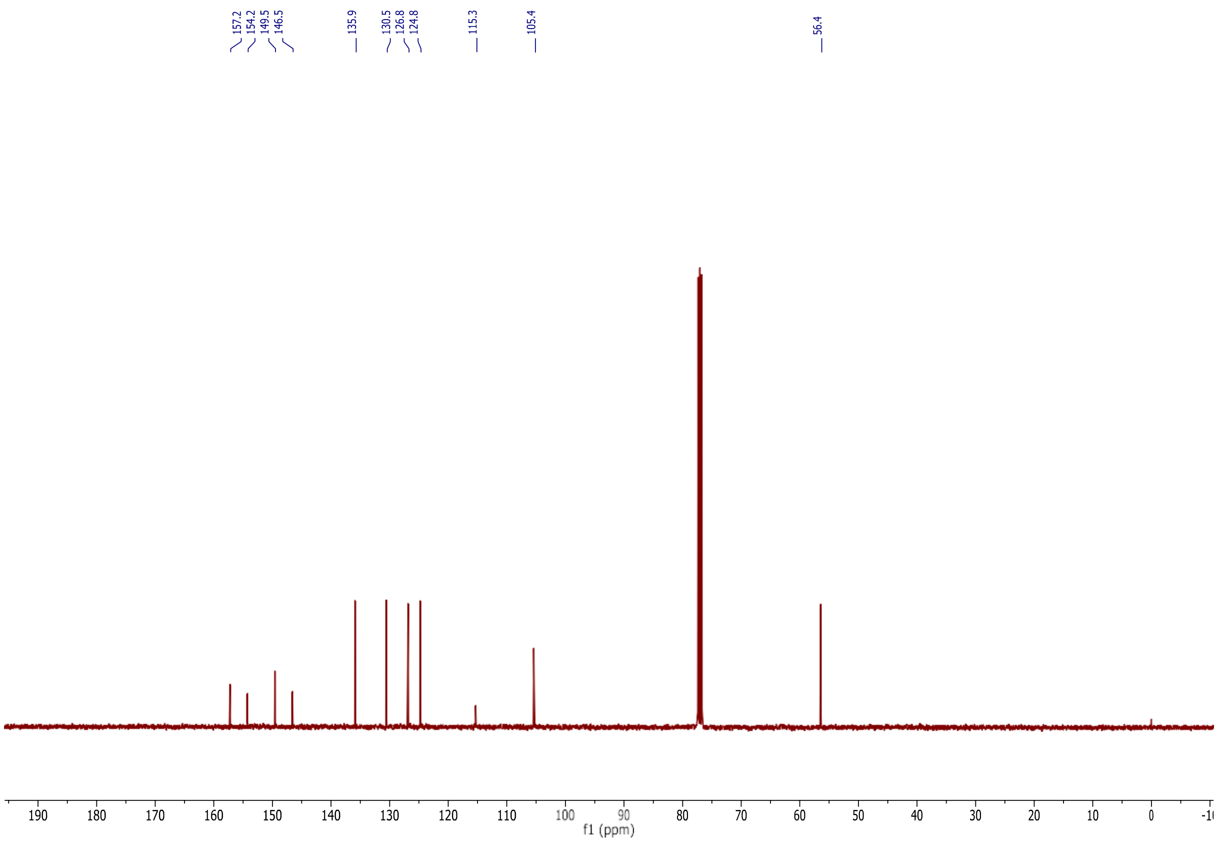
157.2
154.2
149.5
146.5
135.9
130.5
126.8
124.8
115.3
105.4
56.4
190 180 170 160 150 140 130 120 110 100 90 80 70 60 50 40 30 20 10 0
f1 (ppm)

2-phenyl-10H-pyrido[1,2-a]thieno[3,2-d]pyrimidin-10-one

9a

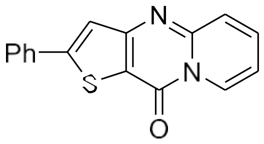

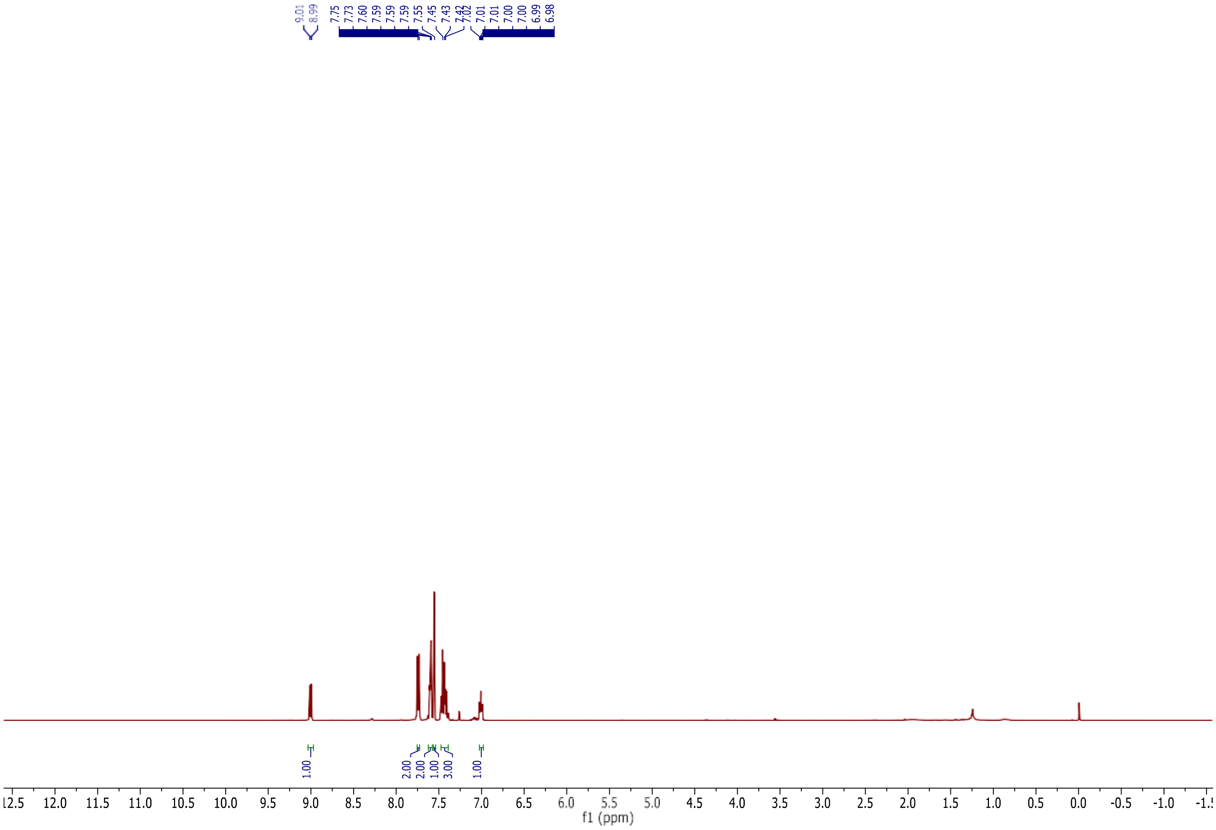

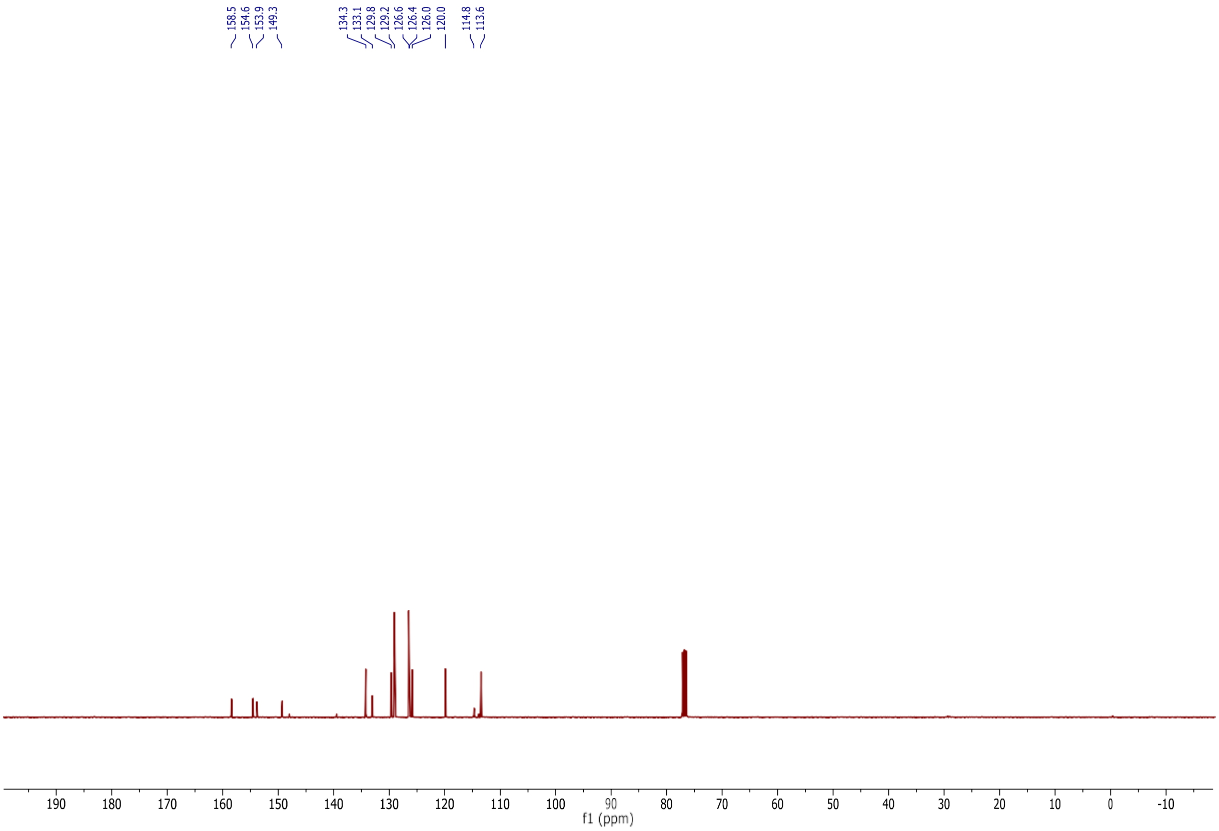
158.5
154.6
153.9
149.3
134.3
133.1
129.8
129.2
126.6
126.4
126.0
120.0
114.8
113.6
190
180
170
160
150
140
130
120
110
100
90
80
70
60
50
40
30
20
10
0
-10
f1 (ppm)

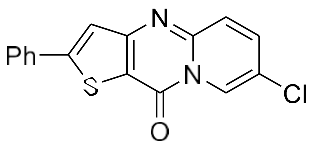

7-chloro-2-phenyl-10H-pyrido[1,2-a]thieno[3,2-d]pyrimidin-10-one ***9b***

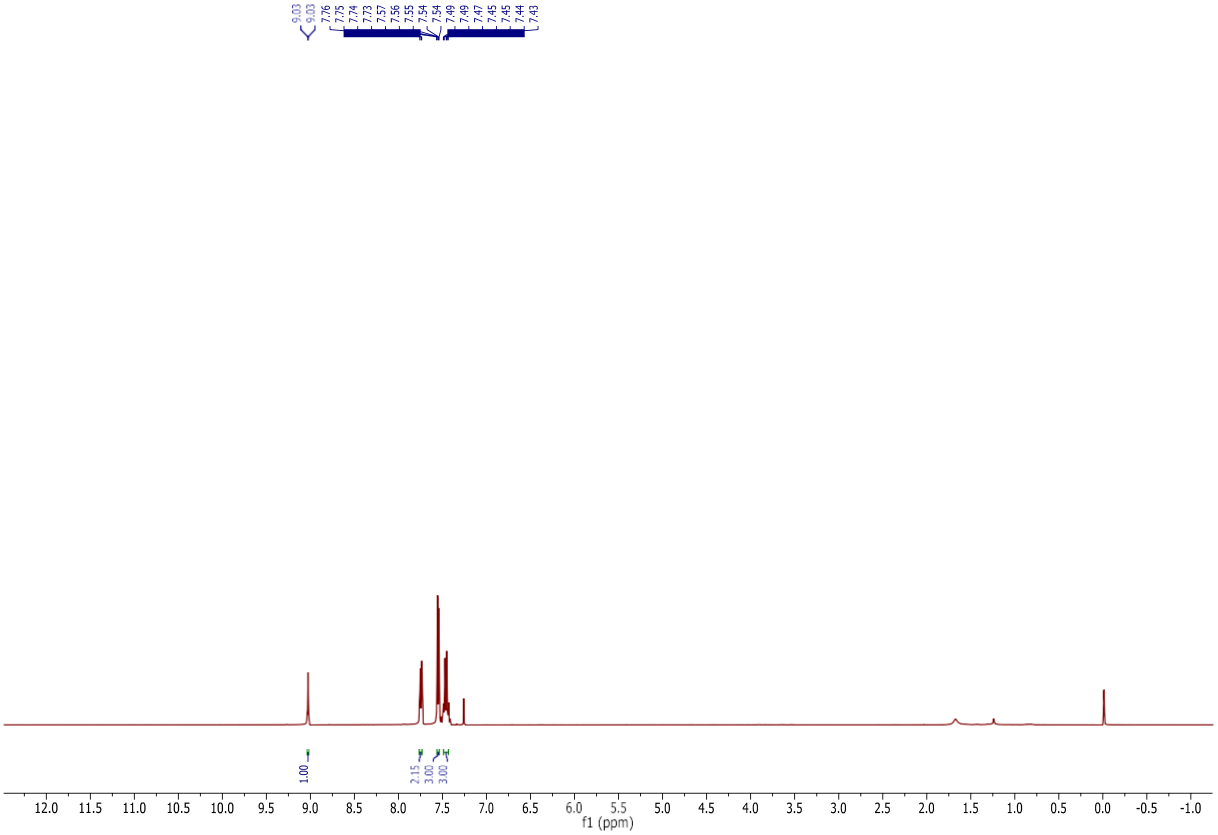

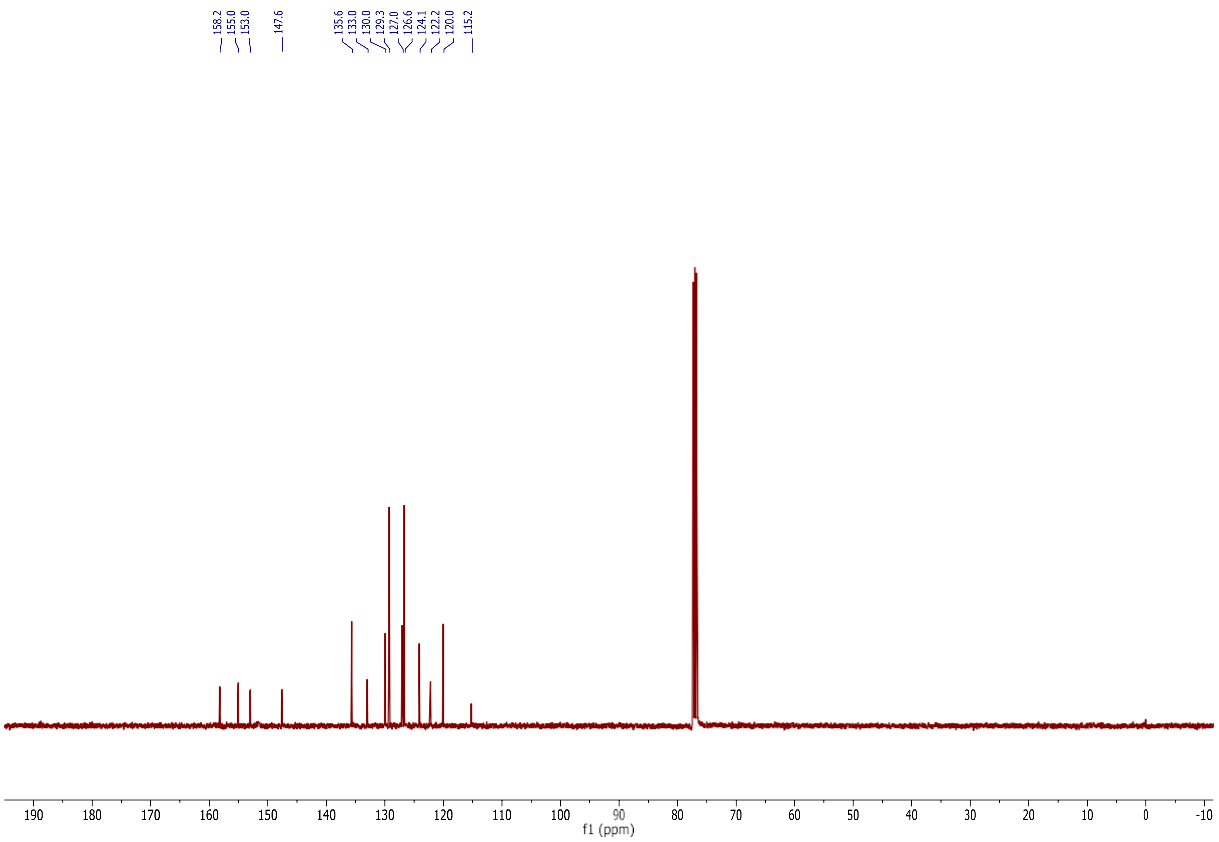
158.2
155.0
153.0
147.6
135.6
133.0
130.0
129.3
127.0
126.6
124.1
122.2
120.0
115.2
190 180 170 160 150 140 130 120 110 100 90 80 70 60 50 40 30 20 10 0 -10
f1 (ppm)

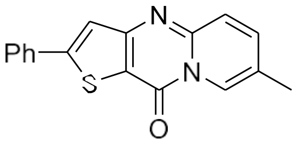

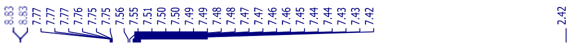

7-methyl-2-phenyl-10H-pyrido[1,2-a]thieno[3,2-d]pyrimidin-10-one ***9c***

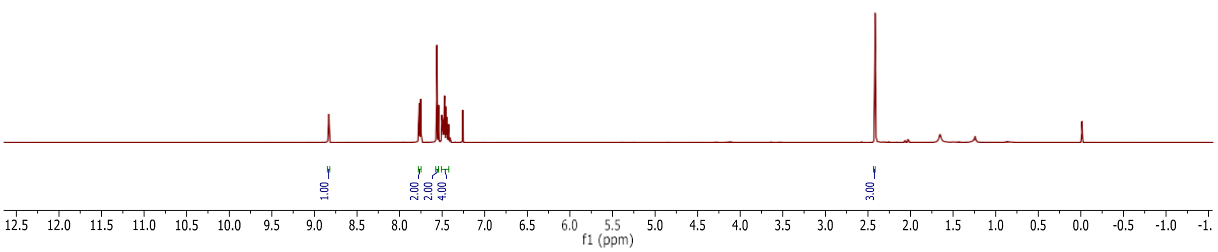

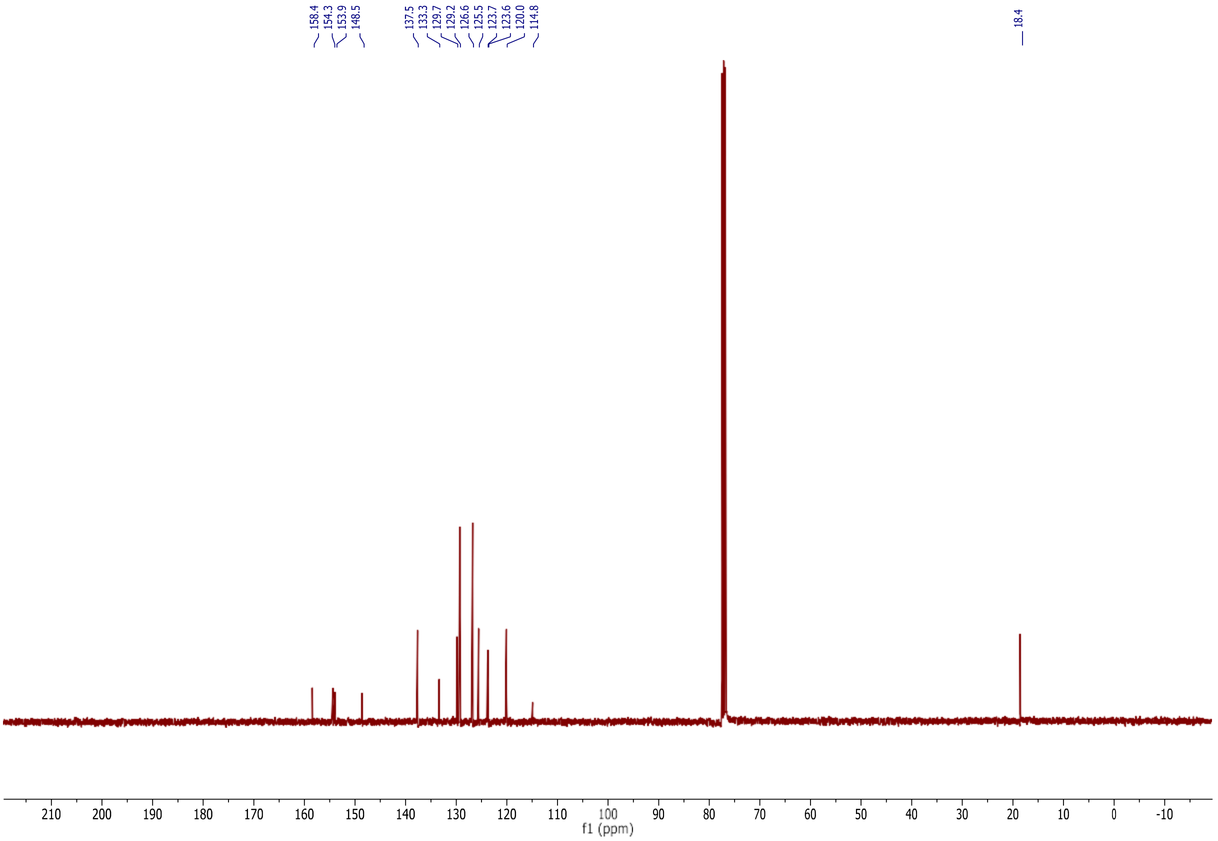
158.4
154.3
153.9
148.5
137.5
133.3
129.7
129.2
126.6
125.5
123.7
123.6
120.0
114.8
18.4
210
200
190
180
170
160
150
140
130
120
110
100
90
80
70
60
50
40
30
20
10
0
-10
f1 (ppm)

7-methoxy-2-phenyl-10H-pyrido[1,2-a]thieno[3,2-d]pyrimidin-10-one ***9d***

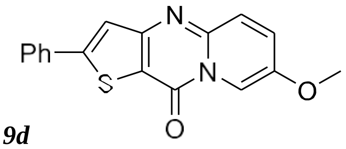

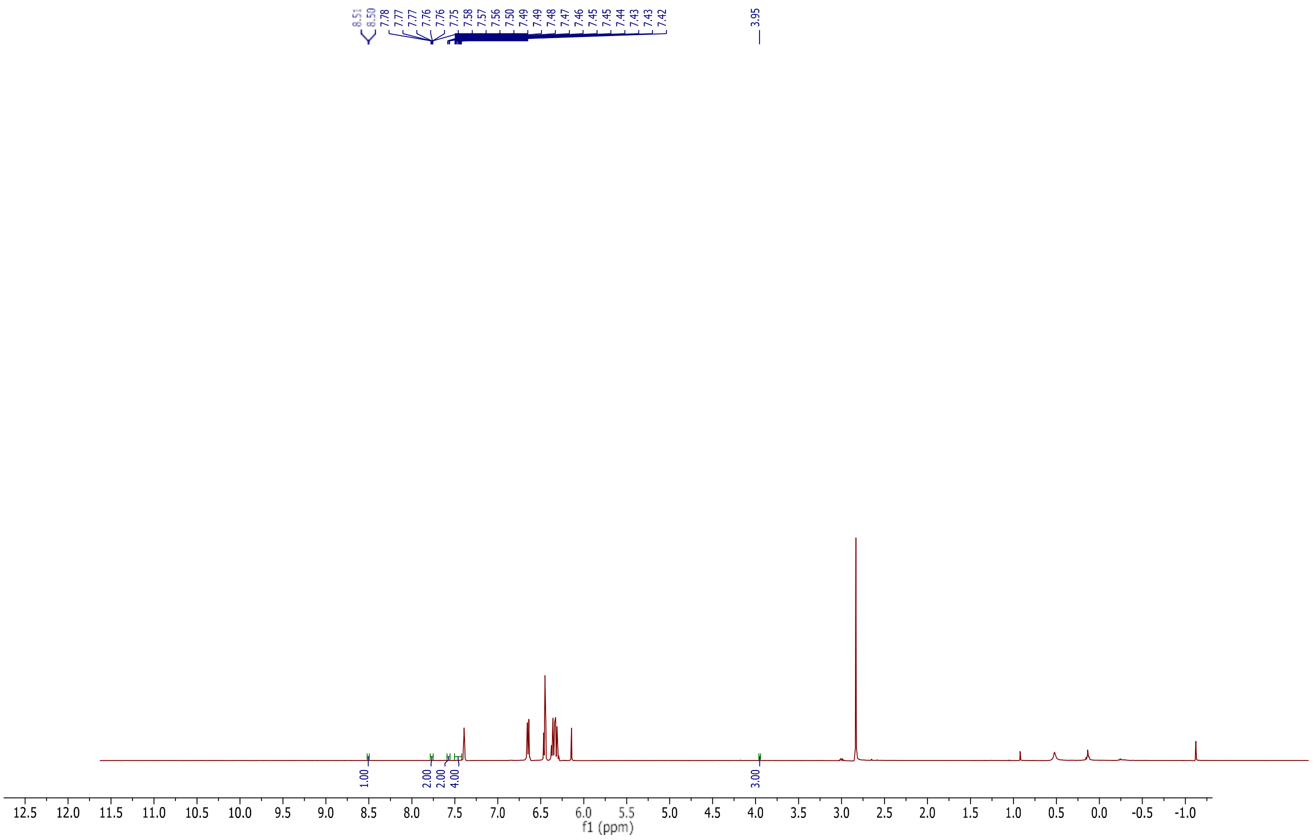

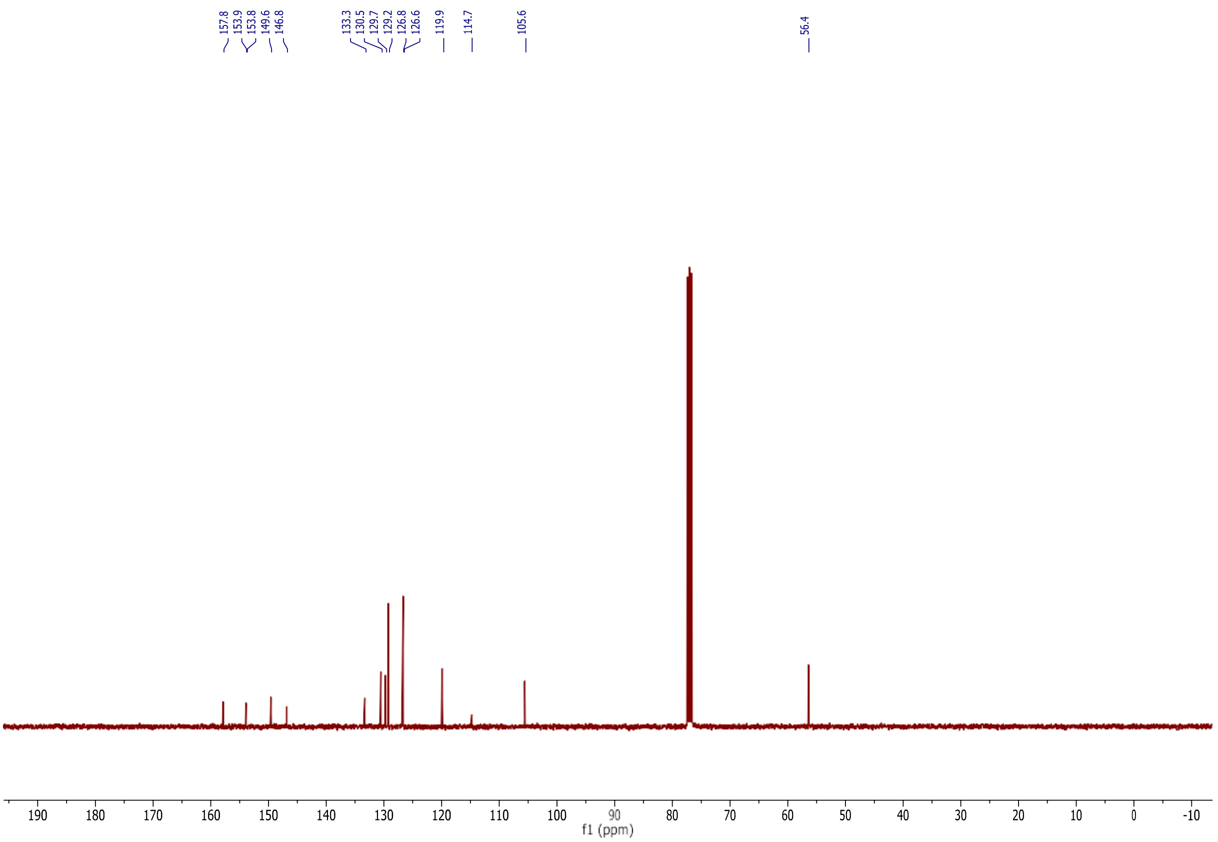
157.8
153.9
153.8
149.6
146.8
133.3
130.5
129.7
129.2
126.8
126.6
119.9
114.7
105.6
56.4
190
180
170
160
150
140
130
120
110
100
90
80
70
60
50
40
30
20
10
0
-10
f1 (ppm)

methyl 5-phenyl-3-(5-((phenylamino)methyl)-1H-1,2,3-triazol-1-yl)thiophene-2-carboxylate ***11a***

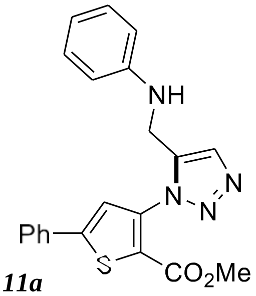

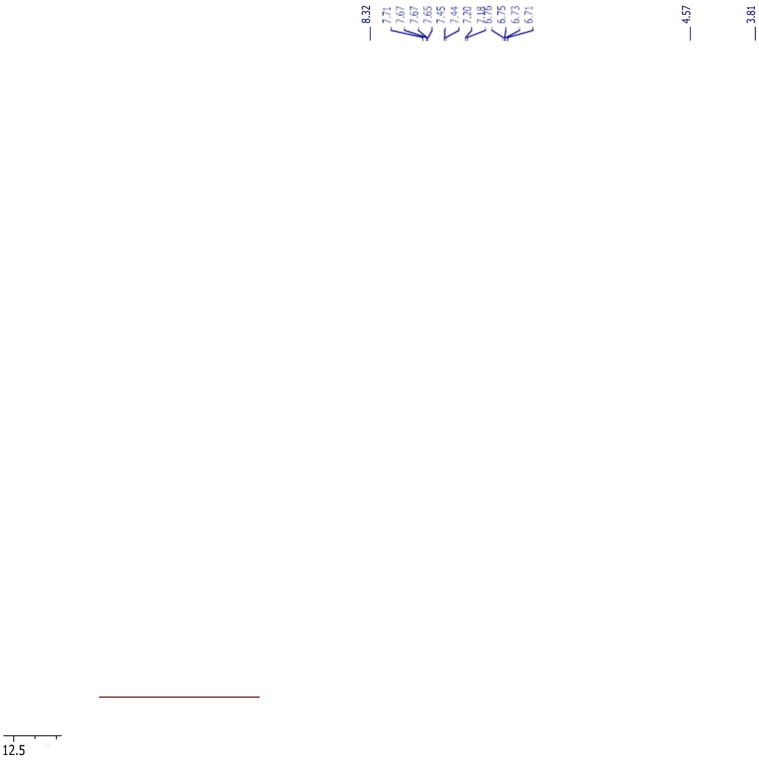

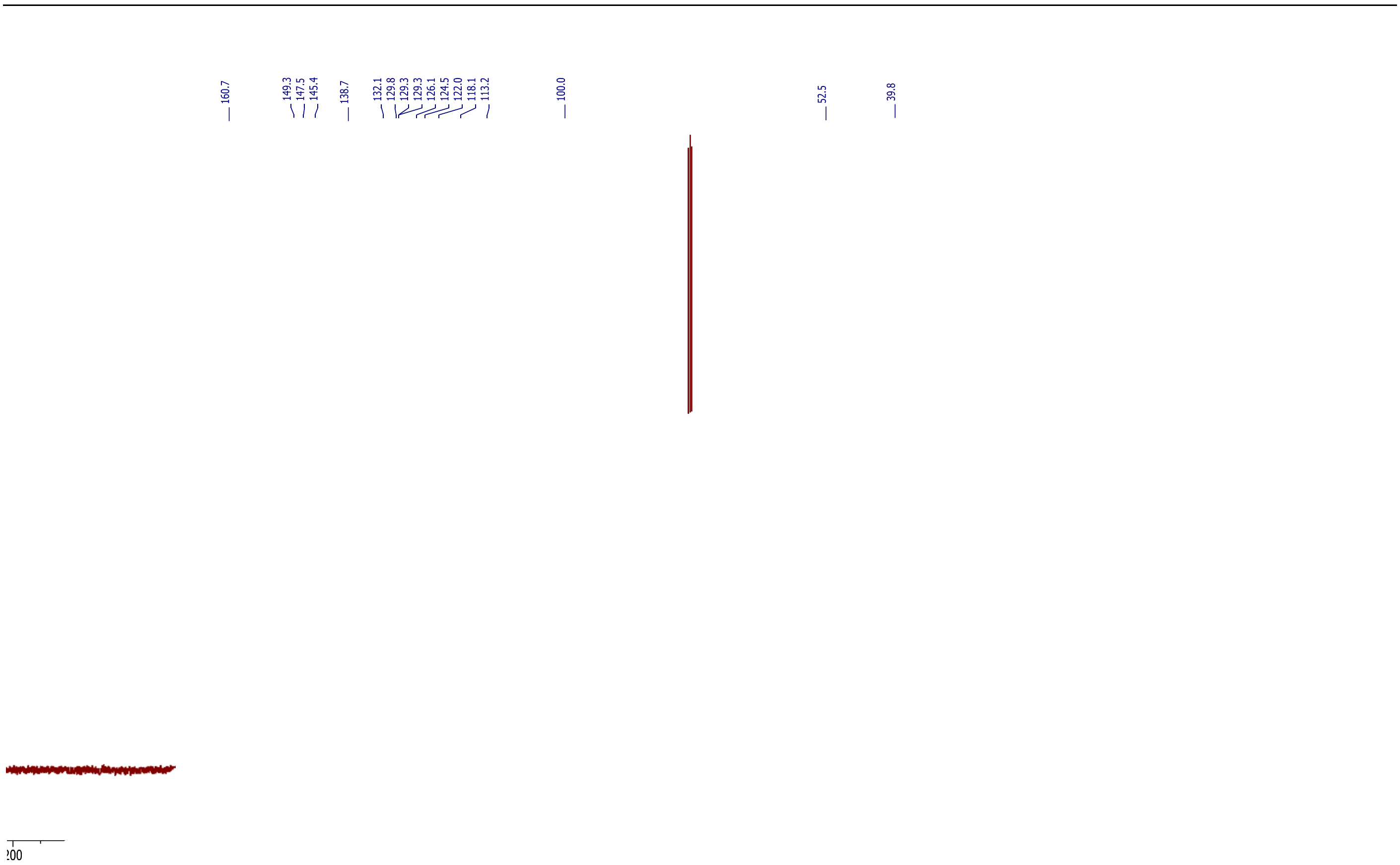
160.7
149.3
147.5
145.4
138.7
132.1
129.8
129.3
129.3
126.1
124.5
122.0
118.1
113.2
100.0
52.5
39.8
200

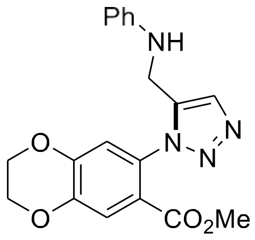

methyl 7-(5-((phenylamino)methyl)-1H-1,2,3-triazol-1-yl)-2,3-dihydrobenzo[b][1,4]dioxine-6-carboxylate ***12a***

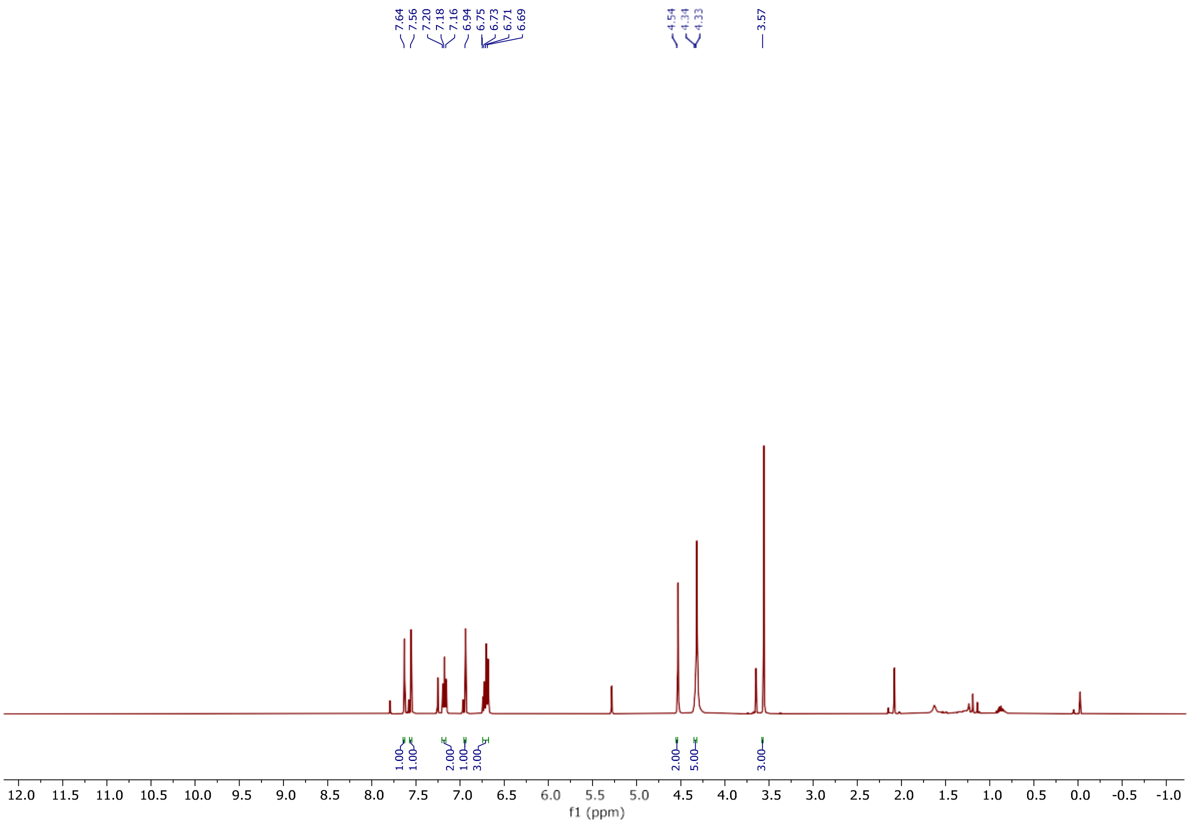

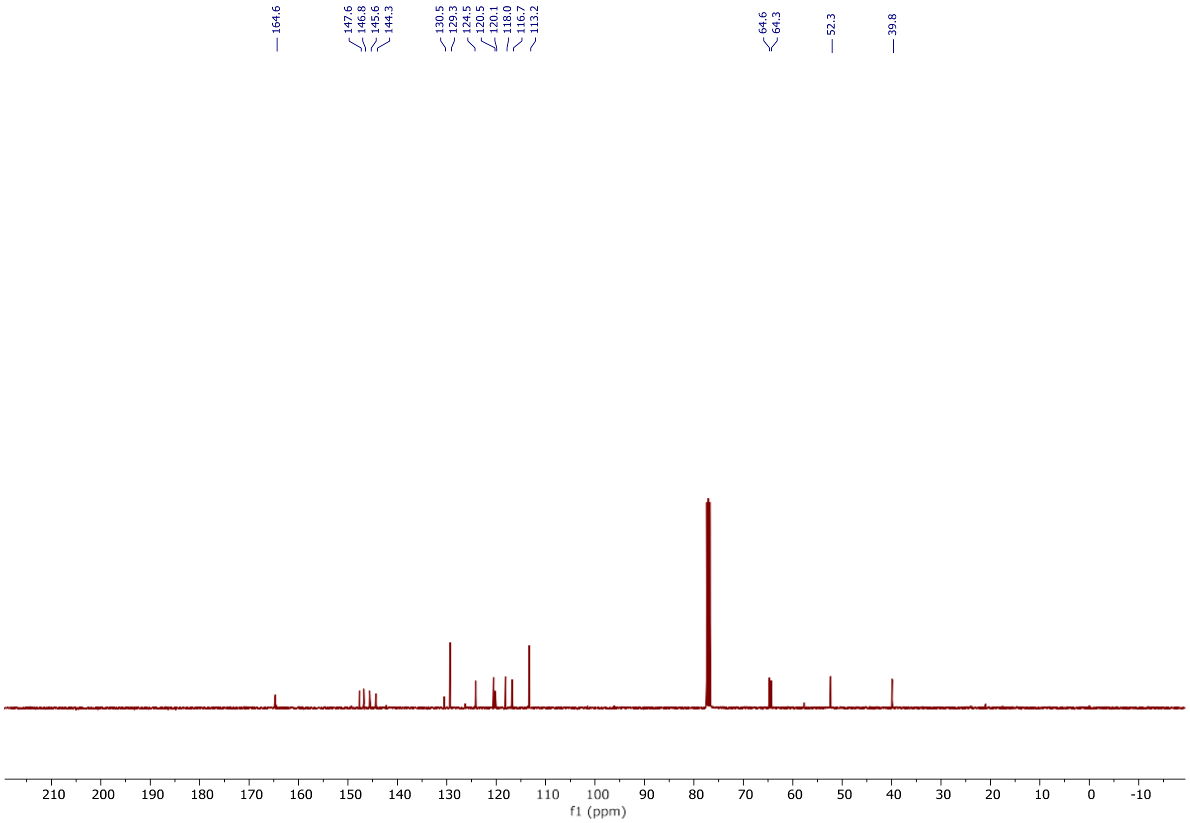
164.6
147.6
146.8
145.6
144.3
130.5
129.3
124.5
120.5
120.1
118.0
116.7
113.2
64.6
64.3
52.3
39.8
210 200 190 180 170 160 150 140 130 120 110 100 90 80 70 60 50 40 30 20 10 0 -10
f1 (ppm)

methyl 6-(5-((phenylamino)methyl)-1H-1,2,3-triazol-1-yl)-1H-indazole-7-carboxylate ***13a***

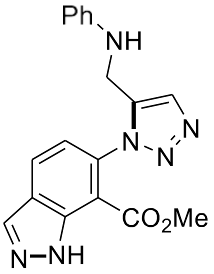

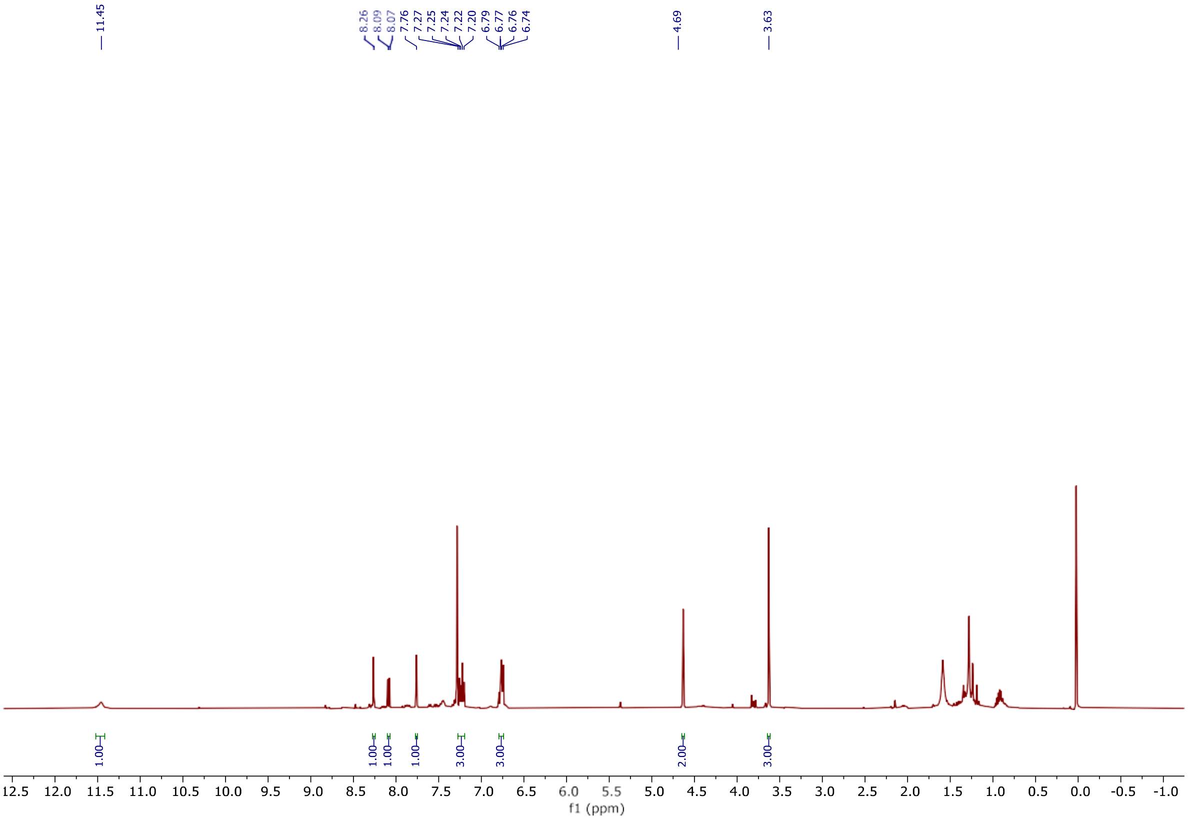

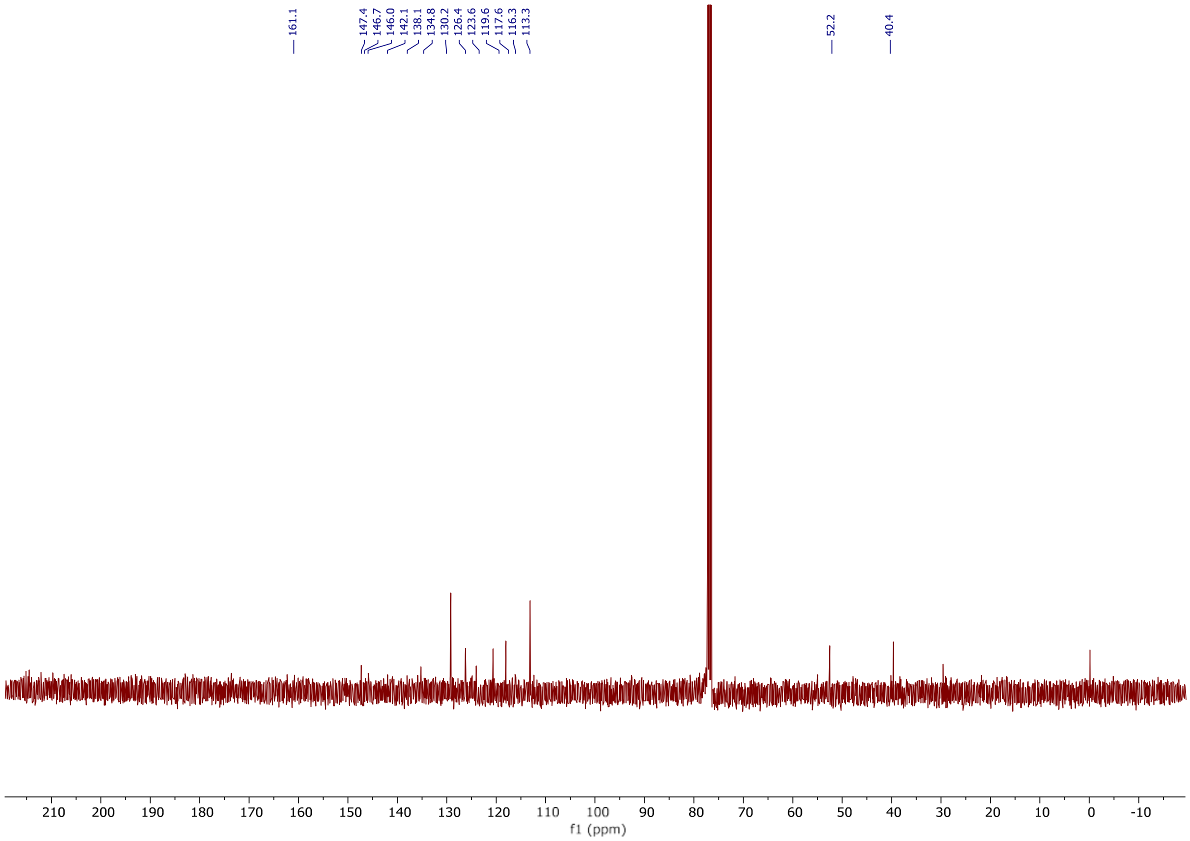

161.1
147.4
146.7
146.0
142.1
138.1
134.8
130.2
126.4
123.6
119.6
117.6
116.3
113.3
52.2
40.4
210 200 190 180 170 160 150 140 130 120 110 100 90 80 70 60 50 40 30 20 10 0 -10
f1 (ppm)

methyl 3-(5-((phenylamino)methyl)-1H-1,2,3-triazol-1-yl)thiophene-2-carboxylate

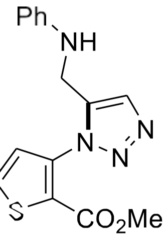

14a

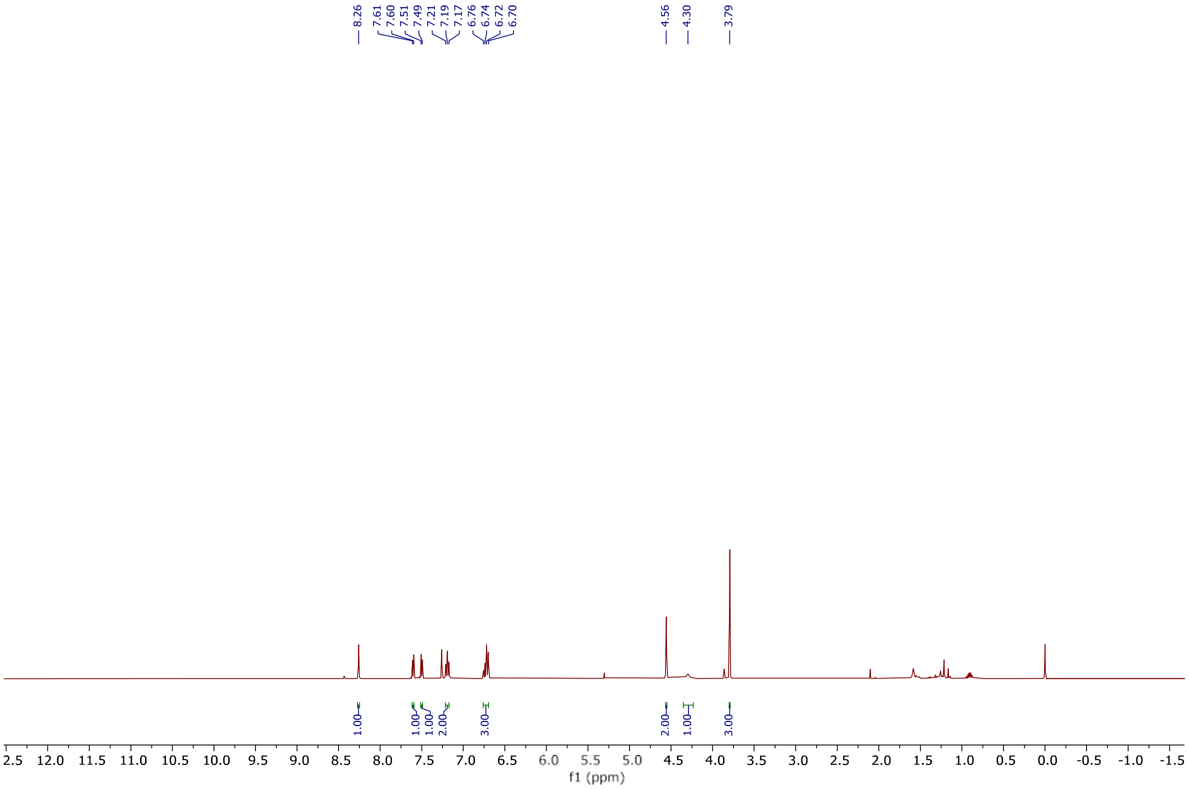

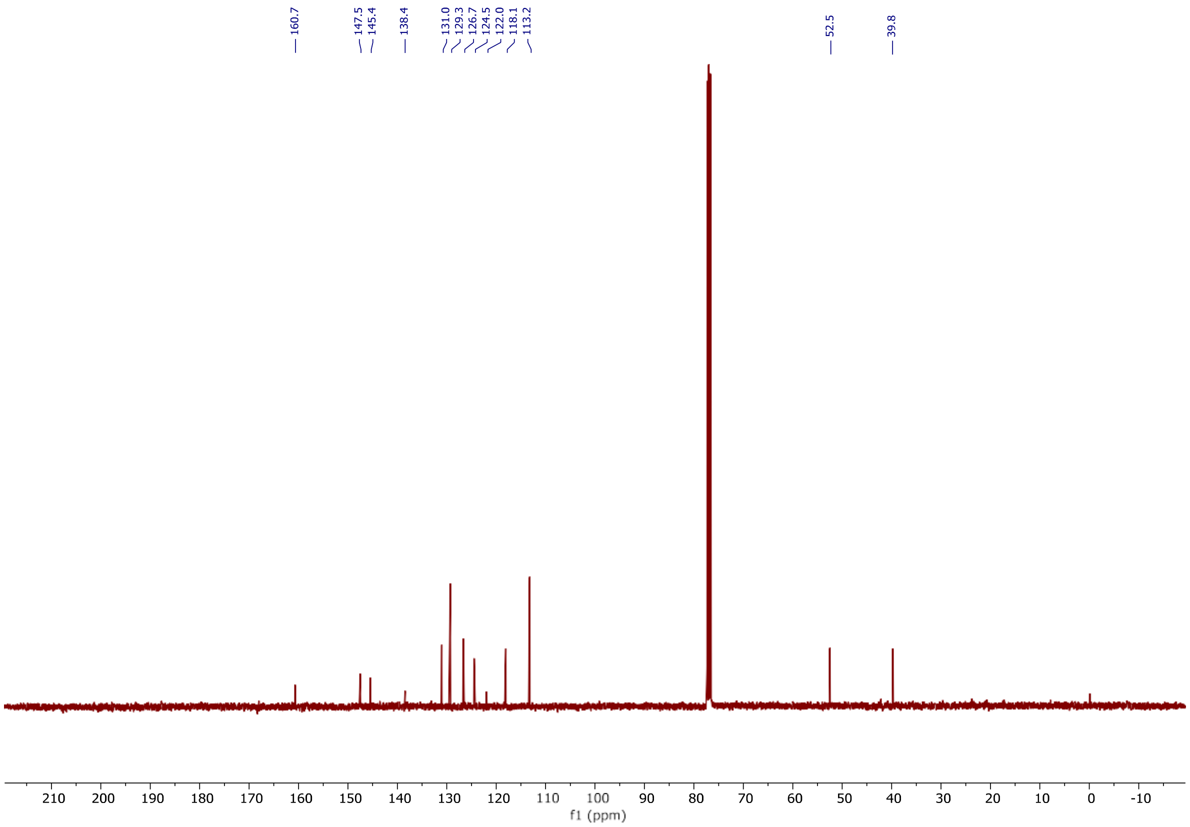
160.7
147.5
145.4
138.4
131.0
129.3
126.7
124.5
122.0
118.1
113.2
52.5
39.8
210 200 190 180 170 160 150 140 130 120 110 100 90 80 70 60 50 40 30 20 10 0 -10
f1 (ppm)

methyl 4-(5-((phenylamino)methyl)-1H-1,2,3-triazol-1-yl)thiazole-5-carboxylate ***15a***

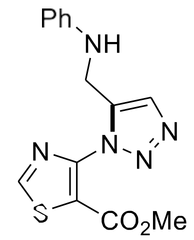

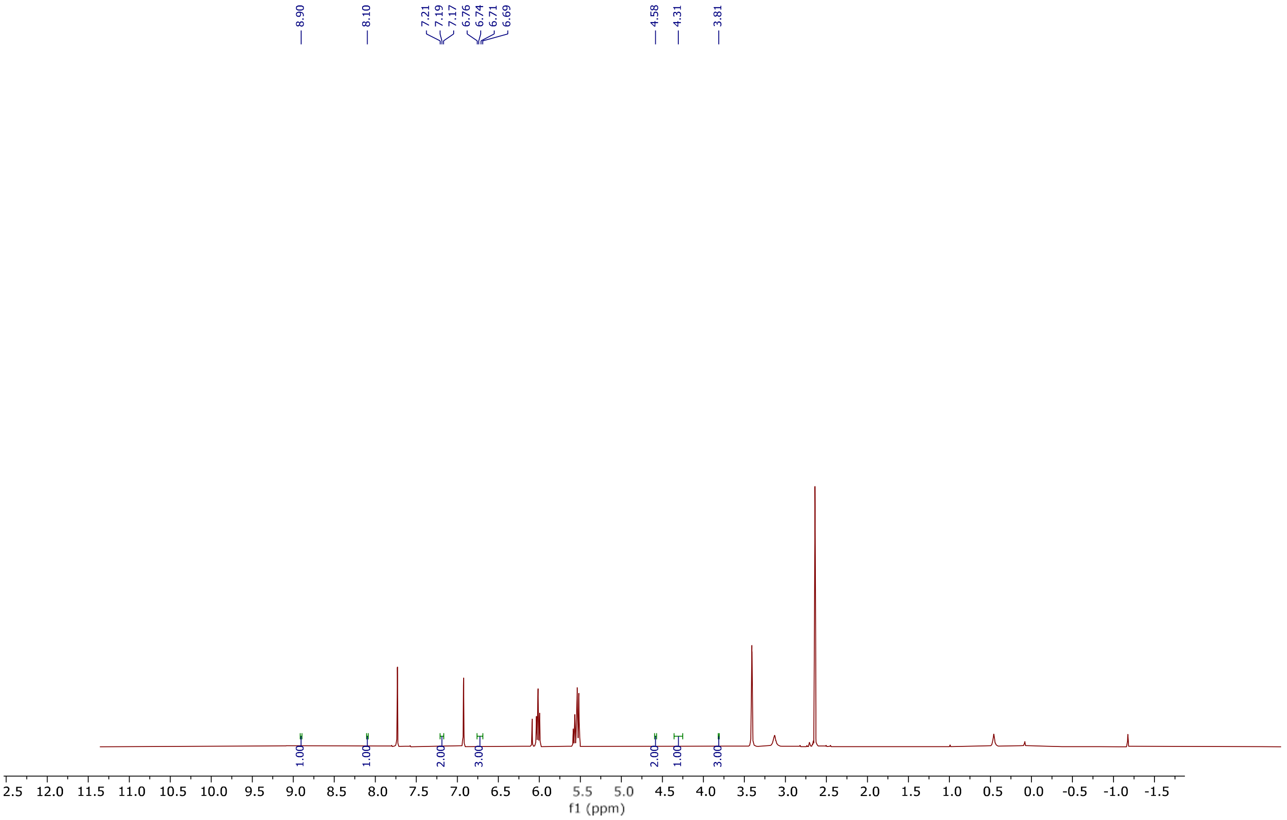

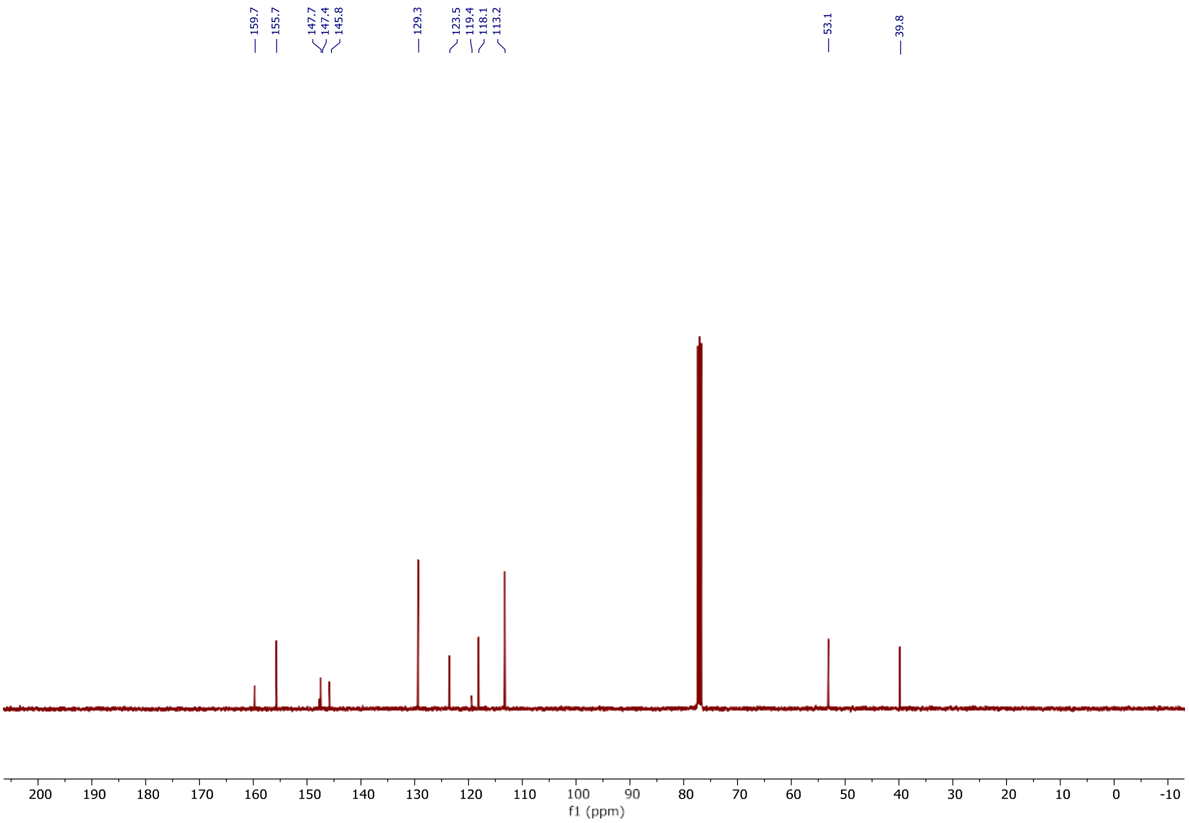
159.7
155.7
147.7
147.4
145.8
129.3
123.5
119.4
118.1
113.2
53.1
39.8
200
190
180
170
160
150
140
130
120
110
100
90
80
70
60
50
40
30
20
10
0
-10
f1 (ppm)

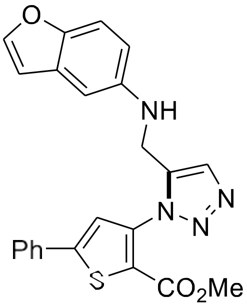

methyl 3-(5-((benzofuran-5-ylamino)methyl)-1H-1,2,3-triazol-1-yl)-5-phenylthiophene-2-carboxylate **11b**

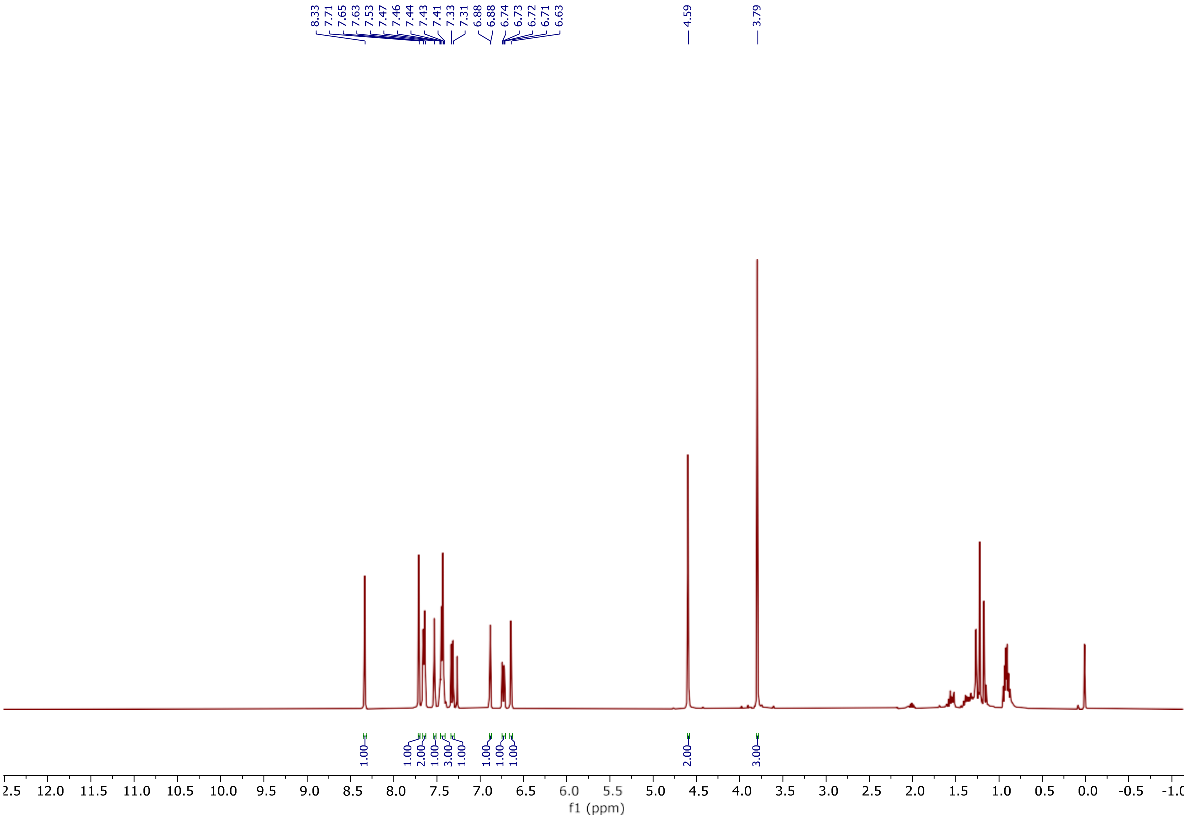

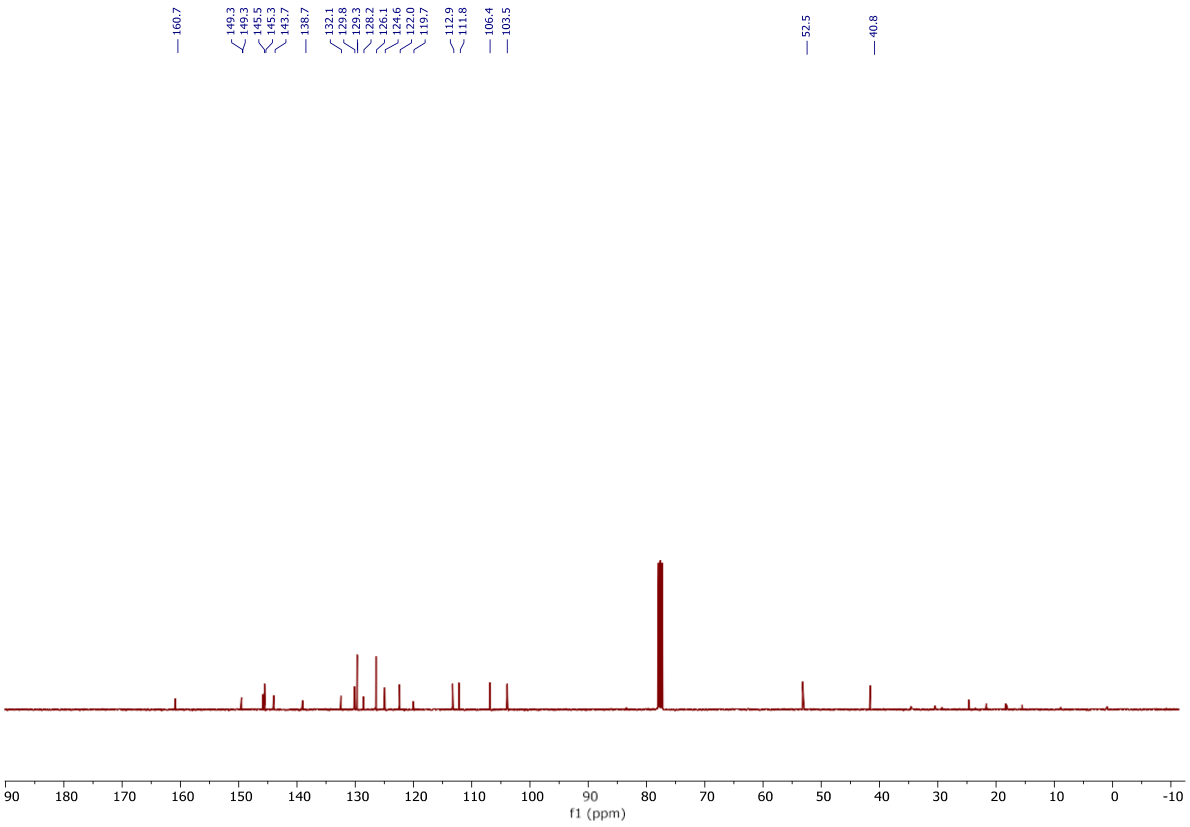
160.7
149.3
149.3
145.5
145.3
143.7
138.7
132.1
129.8
129.3
128.2
126.1
124.6
122.0
119.7
112.9
111.8
106.4
103.5
52.5
40.8
90
180
170
160
150
140
130
120
110
100
90
80
70
60
50
40
30
20
10
0
-10
f1 (ppm)
